# Temporal trends in the prevalence and death of ischemic heart disease in women of childbearing age from 1990 to 2019: a multilevel analysis based on the Global Burden of Disease Study 2019

**DOI:** 10.3389/fcvm.2024.1366832

**Published:** 2024-04-22

**Authors:** Ben Hu, Yan Wang, Dong Chen, Jun Feng, Yinguang Fan, Linlin Hou

**Affiliations:** ^1^ Department of Cardiology, Hefei Hospital Affiliated to Anhui Medical University, Hefei, Anhui, China; ^2^ The Fifth Clinical Medical School of Anhui Medical University, Hefei, Anhui, China; ^3^ Academy of Medical Sciences, Shanxi Medical University, Taiyuan, Shanxi, China; ^4^ Department of Epidemiology and Biostatistics, School of Public Health, Anhui Medical University, Hefei, Anhui, China

**Keywords:** ischemic heart disease, women of childbearing age, prevalence, death, joinpoint regression, Global Burden of Disease Study

## Abstract

**Background:**

Our objective is to describe the current prevalence and death of ischemic heart disease (IHD) in women of childbearing age (WCBA) at the global, regional, and national levels and to analyze its temporal trends from 1990 to 2019.

**Methods:**

WCBA was defined as women aged 15–49 years. Estimates and 95% Uncertainty Intervals (UI) of IHD prevalence and death numbers for seven age groups were extracted from the 2019 Global Burden of Disease Study. The age-standardized prevalence and death rate (ASPR and ASDR) of IHD in WCBA was estimated using the direct age-standardization method. Joinpoint regression analysis was used to calculate average annual percent change (AAPC) to represent the temporal trends from 1990 to 2019.

**Results:**

Between 1990 and 2019, the global ASPR of IHD experienced a 3.21% increase, culminating in 367.21 (95% UI, 295.74–430.16) cases per 100,000 individuals. Conversely, the ASDR decreased to 11.11 (95% UI, 10.10–12.30) per 100,000 individuals. In 2019, among the five sociodemographic index (SDI) regions, the highest ASPR was observed in the high-middle SDI region, whereas the highest ASDR was found in the low-middle SDI region. Regionally, the Caribbean reported the highest ASPR (563.11 per 100,000 individuals; 95% UI, 493.13–643.03), and Oceania reported the highest ASDR (20.20 per 100,000 individuals; 95% UI, 13.01–31.03). At the national level, Trinidad and Tobago exhibited the highest ASPR (730.15 per 100,000 individuals; 95% UI, 633.96–840.13), and the Solomon Islands had the highest ASDR (77.77 per 100,000 individuals; 95% UI, 47.80–121.19). Importantly, over the past three decades, the global ASPR has seen a significant increase [AAPC = 0.11%, 95% Confidence Interval (CI): 0.09–0.13; *P* < 0.001], while the ASDR has demonstrated a significant decreasing trend (AAPC = −0.86%, 95% CI: −1.11 to −0.61; *P* < 0.001). Air pollution, tobacco use, high systolic blood pressure, elevated body mass index, dietary risks, and high LDL cholesterol have been identified as the leading six risk factors for IHD-related deaths among WCBA in 2019.

**Conclusions:**

Despite the significant decline in the global ASDR for IHD among WCBA over the last thirty years, the ASPR continues to escalate. We need to remain vigilant about the increased burden of IHD in WCBA. It calls for aggressive prevention strategies, rigorous control of risk factors, and the enhancement of healthcare coverage to mitigate the disease burden of IHD among WCBA in forthcoming years.

## 1 Introduction

In recent years, the global demographic shift towards an aging population and overall population growth has led to a decline in mortality from infectious diseases and a concurrent rise in the prevalence of chronic diseases (1). Ischemic heart disease (IHD), also referred to as coronary atherosclerotic heart disease, remains the predominant cardiovascular ailment. As a chronic condition, it significantly burdens patients and their families (2). By 2020, IHD and non-communicable diseases had become the leading causes of death worldwide (3). Compared to men, women have a higher incidence of IHD, experience more chronic angina symptoms, undergo more hospital treatments, and have an overall poorer health status (4, 5). Specific conditions unique to females, such as adverse pregnancy outcomes—encompassing hypertensive pregnancy disorders, gestational diabetes, premature births, early menopause, and polycystic ovary syndrome—and the combined use of smoking and oral contraceptives, notably heighten the risk of IHD (6, 7). Despite the decreased prevalence and mortality due to early detection and guided treatment of IHD, understanding of IHD in women remains substantially inadequate. In addition, the United Nations has set goals to reduce the maternal mortality rate by 2030 (8). Therefore, a thorough investigation of epidemiological trends, particularly in Women of Childbearing Age (WCBA) with IHD, is important.

In epidemiological studies, age standardization neutralizes the impact of differences in population age structures, facilitating accurate comparisons of disease burdens across various years and regions. However, the prevalence and death of IHD in WCBA have been surveyed in very limited geographical areas, primarily in specific countries using national health system data, lacking an age-standardization process (9, 10). This kind of survey limits further cross-regional and international comparisons. A global estimation of age-standardized prevalence and death rates of IHD in WCBA over the past decades can fill gaps in disease statistics. A detailed analysis of its changing trends can aid in better understanding the disease epidemiology and promoting medical practice. In this study, we utilized the 2019 Global Burden of Disease Study (GBD) to describe age-standardized prevalence and death rate (ASPR and ASDR) and further analyzed their changes over the past thirty years at global, regional, and national levels.

## 2 Methods

### 2.1 Sociodemographic index

Ranging from 0 to 1, the Sociodemographic Index (SDI) quantifies a country or region's social and demographic development level. A higher level indicates better socio-economic development (11, 12). SDI is associated with disease incidence and mortality rates (13). This study categorizes countries and geographical areas into five distinct SDI regions (high, high-middle, middle, low-middle, and low) to investigate the association between IHD and varying levels of socioeconomic development.

### 2.2 Data sources

The 2019 Global Burden of Disease Study (GBD 2019) estimated the health losses caused by diseases, injuries, and risk factors in 204 countries and regions (14). Detailed information has been previously published (15). To explore the current status and temporal trends of IHD in WCBA, we extracted estimates and 95% Uncertainty Intervals (UI) for IHD prevalence and death numbers across seven age groups (from 15 to 49 years) at the global level, five SDI regions, and 204 countries and territories from GBD 2019. The definition of IHD in this study used the codes from the ninth and tenth editions of the International Classification of Diseases (ICD-9 and ICD-10). Specifically, ICD-9 codes for IHD range from 410 to 414.9 and include V17.3, while ICD-10 codes span I20 to I21.6, I21.9 to I25.9, and Z82.4 to Z82.49 (1). Further details regarding the GBD 2019 data sources are elaborated in the Supplementary Methods.

### 2.3 Temporal trend analysis and statistics

The direct method was used for age standardization, assuming the rate distribution to estimate the ASPR and ASDR of IHD in WCBA, which assumes that the rates are distributed as a weighted sum of independent Poisson random variables (16). Joinpoint regression analysis was employed to analyze the trends in the estimated ASPR and ASDR. This analytical approach delineates points of significant trend alteration, segments the overall trend based on identified junctures, and assesses the epidemiological trend within each segment by calculating Annual Percentage Changes (APCs) and their 95% Confidence Intervals (CIs). Furthermore, epidemiological trends over pre-specified fixed intervals (1990–1999, 2000–2009, 2010–2019, 1990–2019) should be assessed by calculating the Average Annual Percent Change (AAPC) (12). An upward trend is deduced if the AAPC estimate and the lower bound of its 95% CI exceed zero. Conversely, a decreasing trend is indicated if the AAPC estimate and the upper limit of its 95% CI are below zero. Otherwise, the trend is deemed stable over time (17, 18).

### 2.4 Statistical analysis

The environment for joinpoint regression analysis was established using the “configr” package in R version 4.3.0. Data cleaning and computation were facilitated by the “dplyr,” “tidyr,” and “purrr” packages, while the “ggplot2” package was employed for data visualization and the “epitools” package for calculating age-standardized rates. All analyses were conducted using R software (V.4.3.0) (http://www.r-project.org).

## 3 Results

### 3.1 IHD in WCBA: global

From 1990 to 2019, there was a 3.21% increase in the global ASPR of IHD among WCBA, rising from 355.79 per 100,000 individuals (95% UI, 295.74–430.16) in 1990 to 367.21 per 100,000 individuals (95% UI, 306.48–442.43) in 2019 (Table 1). the global ASDR of IHD in WCBA saw a 22.95% decrease, dropping from 11.11 per 100,000 individuals (95% UI, 10.10–12.30) in 1990 to 8.56 per 100,000 individuals (95% UI, 7.55–9.57) in 2019 (Table 1).

**Table 1:** ASPR and ASDR of IHD in WCBA in 1990 and 2019, and change from 1990 to 2019 at the global and regional level.

Location	ASPR, per 100,000 (95% UI)		AAPC (95% CI)	ASDR, per 100,000 (95% UI)		AAPC (95% CI)
	1990	2019		1990	2019	
Global	355.79 (295.74 to 430.16)	367.21 (306.48 to 442.43)	0.11 (0.09 to 0.13)	11.11 (10.1 to 12.3)	8.56 (7.55 to 9.57)	−0.86 (−1.11 to −0.61)
High SDI region	315.18 (268.61 to 372.51)	317.95 (273.96 to 371.08)	0.04 (−0.04 to 0.13)	4.77 (4.58 to 4.98)	3.09 (2.81 to 3.43)	−1.49 (−1.65 to −1.32)
High-middle SDI region	388.14 (318.26 to 475.65)	403.72 (330.23 to 496.02)	0.14 (0.12 to 0.16)	8.78 (7.95 to 9.74)	5.22 (4.61 to 5.87)	−1.7 (−2.05 to −1.34)
Middle SDI region	384.66 (313.55 to 472.74)	397.41 (327.32 to 484.8)	0.12 (0.1 to 0.13)	12.58 (11.29 to 14.03)	8.72 (7.59 to 9.84)	−1.21 (−1.5 to −0.92)
Low-middle SDI region	341.51 (284.25 to 413.68)	353.63 (294.67 to 428.32)	0.13 (0.1 to 0.15)	16.73 (14.24 to 19.8)	13.45 (11.09 to 15.97)	−0.65 (−0.79 to −0.51)
Low SDI region	294.9 (248.18 to 350.23)	305.55 (258.15 to 363.56)	0.12 (0.11 to 0.14)	14.13 (11.41 to 17.66)	11.84 (9.73 to 14.27)	−0.59 (−0.74 to −0.45)
Andean Latin America	148.55 (116.6 to 188.89)	149.57 (117.59 to 190.05)	0.03 (−0.05 to 0.11)	8.68 (6.85 to 10.91)	3.98 (2.85 to 5.4)	−2.78 (−3.41 to −2.15)
Australasia	291.44 (250.86 to 339.68)	242.96 (208.93 to 284.62)	−0.6 (−0.71 to −0.49)	3.91 (3.51 to 4.37)	1.65 (1.4 to 1.93)	−2.86 (−3.15 to −2.57)
Caribbean	578.53 (505.94 to 660.77)	563.11 (493.13 to 643.03)	−0.09 (−0.1 to −0.07)	14.11 (12.01 to 16.83)	10.89 (7.46 to 15.14)	−0.84 (−1.28 to −0.4)
Central Asia	429.18 (365.9 to 504.15)	407.66 (349.01 to 478.38)	−0.16 (−0.22 to −0.1)	13.8 (12.88 to 14.69)	13.46 (11.41 to 15.92)	−0.14 (−0.45 to 0.17)
Central Europe	365.07 (296.76 to 452.13)	334.03 (275.22 to 407.46)	−0.3 (−0.36 to −0.24)	9.05 (8.65 to 9.46)	3.5 (2.91 to 4.22)	−3.3 (−3.64 to −2.95)
Central Latin America	333.55 (279.24 to 400.72)	294.99 (246.39 to 354.2)	−0.42 (−0.45 to −0.39)	8.81 (8.37 to 9.27)	5.35 (4.39 to 6.51)	−1.46 (−1.84 to −1.07)
Central Sub-Saharan Africa	248.19 (212.5 to 291.03)	240.66 (205.51 to 281.46)	−0.1 (−0.12 to −0.08)	8.03 (4.92 to 12.99)	6.86 (4.06 to 11.1)	−0.53 (−0.67 to −0.39)
East Asia	449.92 (349.31 to 577.75)	495.32 (385.1 to 633.68)	0.32 (0.3 to 0.35)	8.45 (6.65 to 10.66)	4.53 (3.51 to 5.7)	−2.1 (−2.88 to −1.32)
Eastern Europe	403.67 (338.74 to 484.39)	439.81 (368.49 to 528.01)	0.33 (0.1 to 0.56)	8.4 (7.36 to 9.03)	8.13 (6.57 to 10.05)	−0.11 (−1.07 to 0.87)
Eastern Sub-Saharan Africa	231.91 (194.26 to 279.32)	242.27 (202.49 to 291.55)	0.15 (0.14 to 0.17)	7.34 (5.46 to 10.46)	5.4 (4.05 to 7)	−1.06 (−1.2 to −0.91)
High-income Asia Pacific	212.91 (179.19 to 253.89)	197.99 (166.82 to 234.97)	−0.22 (−0.29 to −0.16)	3.7 (3.25 to 4.17)	1.04 (0.94 to 1.15)	−4.35 (−4.59 to −4.11)
High-income North America	335.94 (283.8 to 396.21)	335.94 (283.8 to 396.21)	−0.02 (−0.11 to 0.08)	5.7 (5.46 to 5.91)	4.34 (4.09 to 4.74)	−0.9 (−1.18 to −0.63)
North Africa and Middle East	537.35 (465.79 to 621.67)	527.41 (456.12 to 609.37)	−0.05 (−0.11 to 0.02)	27.69 (24.06 to 32.21)	15.97 (12.71 to 19.83)	−1.85 (−1.96 to −1.74)
Oceania	414 (330.66 to 523.01)	422.63 (334.29 to 531.53)	0.08 (0.04 to 0.12)	17.89 (11.41 to 27.37)	20.2 (13.01 to 31.03)	0.4 (0.27 to 0.53)
South Asia	352.48 (293.12 to 426.4)	368.23 (306.01 to 445.93)	0.15 (0.14 to 0.17)	18.87 (15.78 to 22.42)	15.95 (12.77 to 19.55)	−0.56 (−0.79 to −0.33)
Southeast Asia	262.04 (215.18 to 319.12)	276.05 (226.65 to 335.98)	0.19 (0.12 to 0.25)	12.86 (10.61 to 15.86)	9.73 (7.97 to 11.88)	−0.96 (−1.11 to −0.81)
Southern Latin America	157.75 (133.91 to 186.18)	145.83 (124.49 to 172.63)	−0.26 (−0.3 to −0.22)	6.08 (5.4 to 6.85)	2.7 (2.24 to 3.2)	−2.73 (−3 to −2.46)
Southern Sub-Saharan Africa	290.57 (243.87 to 346.87)	261.92 (220.19 to 312.35)	−0.35 (−0.39 to −0.31)	8.48 (7 to 10.12)	4.7 (3.28 to 6.41)	−2.14 (−3.1 to −1.17)
Tropical Latin America	207.33 (166.81 to 257.87)	197.42 (159.68 to 244.62)	−0.16 (−0.19 to −0.14)	12.22 (11.51 to 12.95)	6 (5.5 to 6.54)	−2.4 (−2.64 to −2.16)
Western Europe	326.32 (278.73 to 384.27)	310.6 (268.03 to 361.56)	−0.14 (−0.22 to −0.06)	3.62 (3.45 to 3.79)	1.46 (1.35 to 1.58)	−3.08 (−3.27 to −2.88)
Western Sub-Saharan Africa	228.9 (194.35 to 270.79)	248.68 (210.45 to 295.94)	0.3 (0.26 to 0.34)	7.89 (5.84 to 10.62)	6.36 (4.73 to 8.52)	−0.74 (−0.96 to −0.52)

ASPR, age-standardized prevalence rate; ASDR, age-standardized death rate; IHD, ischemic heart disease; WCBA, women of childbearing age; SDI, sociodemographic index; UI, uncertainty interval; AAPC, average annual percent change; CI, confidence interval.

### 3.2 IHD in WCBA: SDI regional analysis

The high-middle SDI region exhibited the most significant increase in ASPR, at 4.01%, with the highest ASPR recorded at 403.72 cases per 100,000 individuals (95% UI, 330.23–496.02) (Table 1). This region also witnessed the largest decline in IHD-related deaths (40.55%) across the five SDI regions. In 2019, the highest IHD-related ASDR was in the low-middle SDI region, standing at 13.45 per 100,000 individuals (95% UI, 11.09–15.97) (Table 1).

### 3.3 IHD in WCBA: geographic regional analysis

Among the 21 geographic regions assessed, East Asia reported the highest ASPR of IHD in WCBA in 2019 (563.11 per 100,000; 95% UI, 493.13–643.03), while Southern Latin America had the lowest (145.83 per 100,000; 95% UI, 124.49–172.63). Seven regions (South Asia, Oceania, North Africa and the Middle East, Eastern Europe, East Asia, Central Asia, and the Caribbean) reported an ASPR above the global average (367.21 per 100,000), whereas fourteen regions were below this benchmark. In addition, Oceania recorded the highest ASDR due to IHD in WCBA (20.2 per 100,000; 95% UI, 13.01–31.03), and the high-income Asia Pacific region had the lowest (1.04 per 100,000; 95% UI, 0.94–1.15). Six regions (Southeast Asia, South Asia, Oceania, North Africa and Middle East, Central Asia, and the Caribbean) reported death rates exceeding the global average, while fifteen had lower rates (8.56 per 100,000) (Table 1).

### 3.4 National analysis of IHD Among WCBA

**Figure 1:**
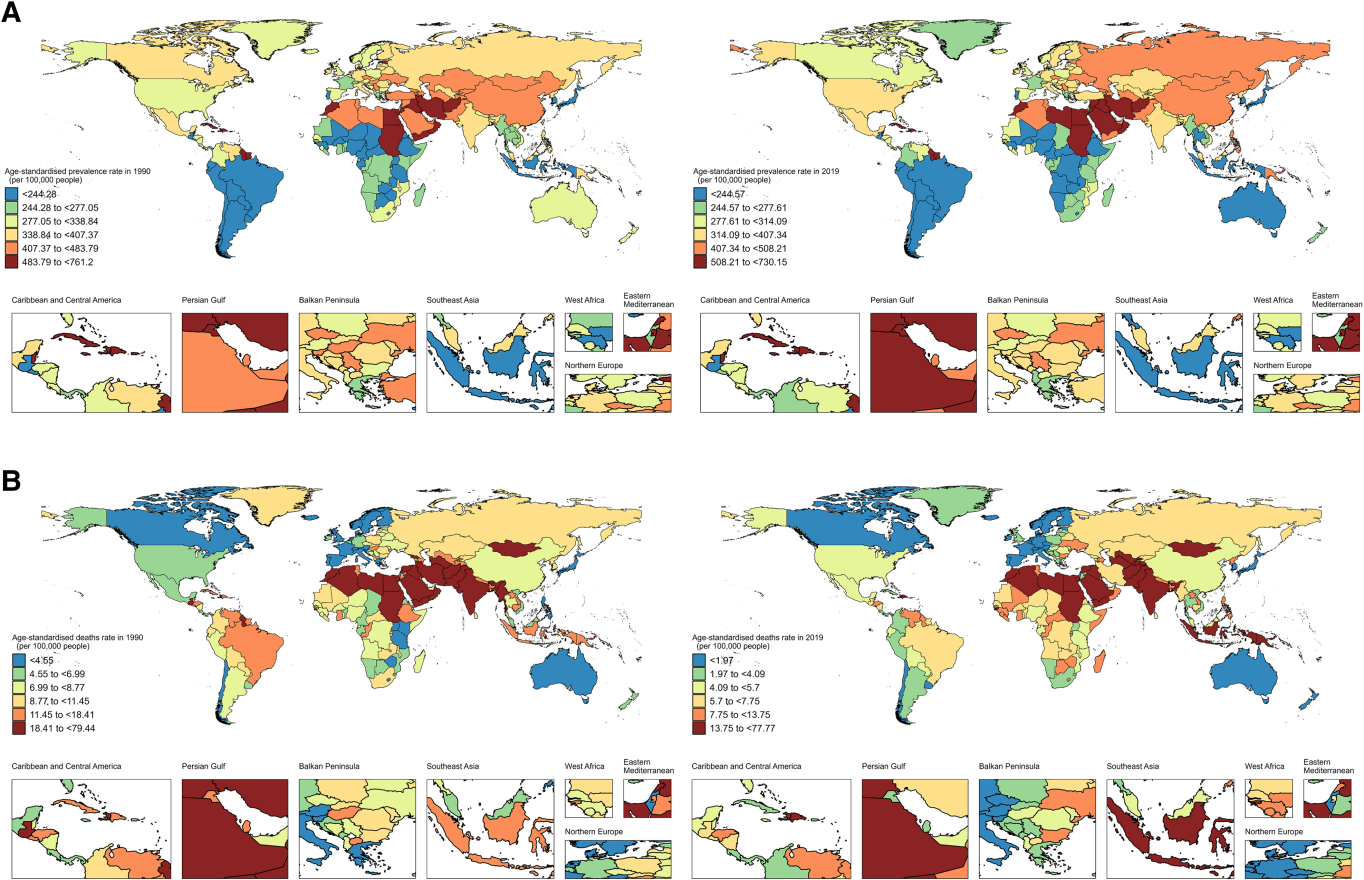
World map of ASPR (**A**) and ASDR (**B**) of IHD in WCBA in 1990 and 2019. ASPR, age-standardized prevalence rate; ASDR, age-standardized death rate; IHD, ischemic heart disease; WCBA, women of childbearing age.

In 2019, the global average of ASPR was 367.21 per 100,000 individuals; across 204 countries, Trinidad and Tobago reported the highest ASPR of IHD among WCBA, at 730.15 per 100,000 individuals (95% UI, 633.96–840.13). With ASPR of 79 countries exceeding the global average and ASPR of 125 countries below the global average. (Supplementary Table S1 and Figure 1A). Additionally, the global average of ASDR was 8.56 per 100,000 individuals; the Solomon Islands had the highest IHD-related ASDR in WCBA (77.76 per 100,000; 95% UI, 47.80–121.19). Fifty-nine countries experienced an ASDR above the global average, whereas 145 countries had ASDR below the global average (Supplementary Table S5 and Figure 1B).

### 3.5 Temporal trends of IHD Among WCBA

**Figure 2:**
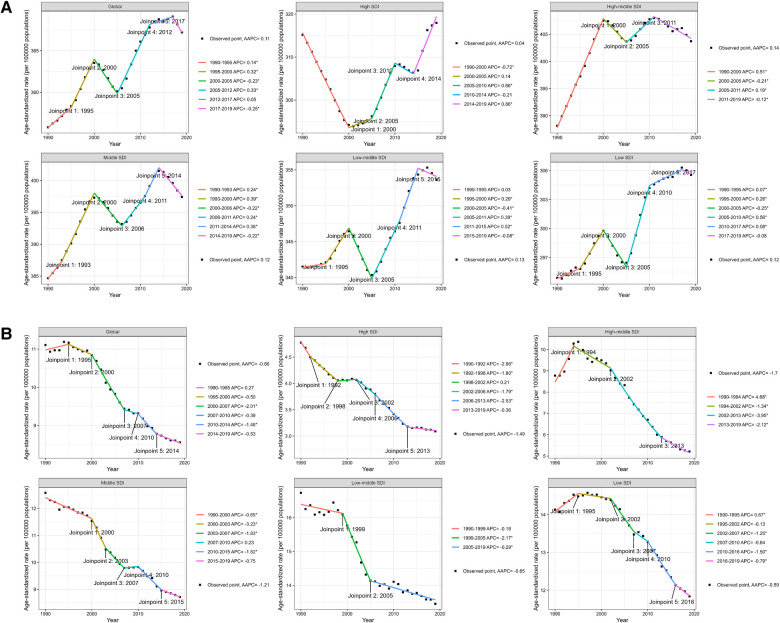
Joinpoint regression analysis of ASPR (**A**) and ASDR (**B**) of IHD in WCBA at the global and five SDI levels from 1990 to 2019. ASPR, age-standardized prevalence rate; ASDR, age-standardized death rate; IHD, ischemic heart disease; WCBA, women of childbearing age. *P*-value *P < 0.05.

**Figure 3:**
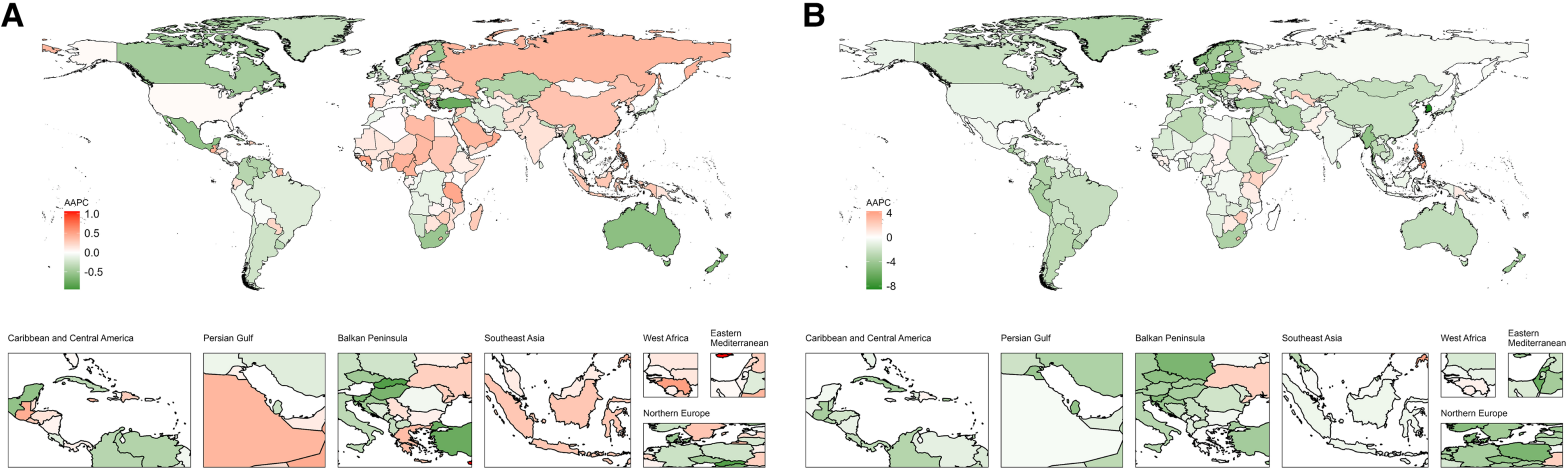
World map of AAPCs in ASPR (**A**) and ASDR (**B**) of IHD in WCBA from 1990 to 2019. AAPC, average annual percentage change; IHD, ischemic heart disease; WCBA, women of childbearing age.

From 1990 to 2019, the global ASPR of IHD in WCBA showed a significant increasing trend (AAPC = 0.11%, 95% CI: 0.09–0.13; *P* < 0.001) (Table 2 and Figure 2A). The most pronounced change occurred between 2005 and 2012 (APC = 0.33%, 95% CI: 0.30–0.36; *P* < 0.001) (Figure 2A and Supplementary Table S3). At the level of SDI regions, the ASPR of IHD in WCBA significantly increased in the high-middle, middle, low-middle, and low SDI regions, with no significant trend observed in the high SDI regions (Figure 2A and Supplementary Table S3). In the high SDI regions, a rapid decline in ASPR was noted before 2000 (APC = −0.72%, 95% CI: −0.79 to −0.66; *P* < 0.001), followed by a stepped increase, particularly between 2005 and 2010 (APC = 0.86%, 95% CI: 0.60–1.11; *P* < 0.001) and 2014–2019 (APC = 0.86%, 95% CI: 0.68–1.03; *P* < 0.001) (Figure 2A and Supplementary Table S3). Eastern Europe saw the largest increase in ASPR (AAPC = 0.33%, 95% CI: 0.10–0.56; *P* < 0.001), while Australasia experienced the most significant decrease (AAPC = −0.60%, 95% CI: −0.71 to −0.49; *P* < 0.001) (Table 1). At the country level, from 1990 to 2019, Cyprus had the largest increase in ASPR (AAPC = 1.06%, 95% CI: 0.36–1.77; *P* < 0.001), and Singapore experienced the most notable decrease (AAPC = −0.95%, 95% CI: −0.99 to −0.90; *P* < 0.001). Ninety-two countries showed increasing trends in ASPR of IHD in WCBA, while 76 countries exhibited a decrease, and 36 remained relatively stable (Figure 3A and Supplementary Tables S2).

**Table 2:** AAPCs of global and SDI region in ASPR and ASDR of IHD in WCBA.

		IHD			
		ASPR		ASDR	
		AAPC (95% CI)	*p*-value	AAPC (95% CI)	*p*-value
	1990–1999	0.22 (0.19 to 0.25)	<0.001	−0.07 (−0.43 to 0.29)	0.694
Global	2000–2009	0.02 (−0.01 to 0.05)	0.271	−1.65 (−2.05 to −1.25)	<0.001
	2010–2019	0.04 (0 to 0.09)	0.072	−0.94 (−1.38 to −0.50)	<0.001
	1990–2019	0.11 (0.09 to 0.13)	<0.001	−0.86 (−1.11 to −0.61)	<0.001
	1990–1999	−0.72 (−0.79 to −0.65)	<0.001	−1.84 (−2.12 to −1.55)	<0.001
High SDI	2000–2009	0.46 (0.29 to 0.63)	<0.001	−1.60 (−1.87 to −1.33)	<0.001
	2010–2019	0.38 (0.19 to 0.57)	<0.001	−1.09 (−1.34 to −0.83)	<0.001
	1990–2019	0.04 (−0.04 to 0.13)	0.340	−1.49 (−1.65 to −1.32)	<0.001
	1990–1999	0.51 (0.48 to 0.53)	<0.001	1.29 (0.46 to 2.13)	<0.001
High-middle SDI	2000–2009	−0.03 (−0.08 to 0.02)	0.205	−3.38 (−3.64 to −3.11)	<0.001
	2010–2019	−0.09 (−0.11 to −0.06)	<0.001	−2.74 (−3.34 to −2.13)	<0.001
	1990–2019	0.14 (0.12 to 0.16)	<0.001	−1.7 (−2.05 to −1.34)	<0.001
	1990–1999	0.34 (0.31 to 0.36)	<0.001	−0.65 (−0.82 to −0.47)	<0.001
Middle SDI	2000–2009	−0.07 (−0.09 to −0.04)	<0.001	−1.84 (−2.55 to −1.13)	<0.001
	2010–2019	0.03 (−0.02 to 0.07)	0.206	−1.35 (−1.78 to −0.91)	<0.001
	1990–2019	0.12 (0.10 to 0.13)	<0.001	−1.21 (−1.5 to −0.92)	<0.001
	1990–1999	0.14 (0.11 to 0.18)	<0.001	−0.18 (−0.48 to 0.07)	0.153
Low-middle SDI	2000–2009	−0.05 (−0.09 to −0.02)	0.005	−1.34 (−1.62 to −1.06)	<0.001
	2010–2019	0.24 (0.19 to 0.29)	<0.001	−0.29 (−0.41 to −0.18)	<0.001
	1990–2019	0.13 (0.10 to 0.15)	<0.001	−0.65 (−0.79 to −0.51)	<0.001
	1990–1999	0.16 (0.13 to 0.18)	<0.001	0.31 (0.15 to 0.48)	<0.001
Low SDI	2000–2009	0.11 (0.08 to 0.13)	<0.001	−0.86 (−1.14 to −0.59)	<0.001
	2010–2019	0.04 (0.01 to 0.07)	0.007	−1.26 (−1.48 to −1.05)	<0.001
	1990–2019	0.12 (0.11 to 0.14)	<0.001	−0.59 (−0.74 to −0.45)	<0.001

ASPR, age-standardized prevalence rate; ASDR, age-standardized death rate; IHD, ischemic heart disease; WCBA, women of childbearing age; SDI, sociodemographic index; AAPC, average annual percentage change; CI, confidence interval.

Regarding the ASDR of IHD in WCBA, a significant global downward trend was observed from 1990 to 2019 (AAPC = −0.86%, 95% CI: −1.11 to −0.61; *P* < 0.001) (Table 2 and Figure 2B), with the most significant change between 2000 and 2007 (APC = −2.01%, 95%CI: −2.31 to −1.72; *P* < 0.001) (Figure 2B and Supplementary Table S7). This declining trend was consistent across SDI regions, with significant decreases occurring at various intervals (Figure 2B and Supplementary Table S7). Oceania recorded the largest increase in ASDR (AAPC = 0.40%, 95% CI: 0.27–0.53; *P* < 0.001), whereas the high-income Asia Pacific region saw the most substantial decrease (AAPC = −4.35%, 95% CI: −4.59 to −4.11; *P* < 0.001) (Table 1). At the national level, between 1990 and 2019, the Philippines experienced the most significant increase in the ASDR for IHD among WCBA, with an AAPC of 4.25% (95% CI, 3.76–4.75; *P* < 0.001). Conversely, the Republic of Korea witnessed the most substantial decrease in ASDR, with an AAPC of −8.52% (95% CI, −8.82 to −8.21; *P* < 0.001). Thirty countries showed an increasing trend in the ASDR of IHD in WCBA, whereas 149 countries exhibited a decrease, and 25 remained relatively stable (Figure 3B and Supplementary Tables S6). Additionally, over the past three decades, significant shifts in ASPR and ASDR occurred during different periods across various countries (Supplementary Tables S4, S8).

### 3.6 Risk factors for IHD Among WCBA

**Figure 4:**
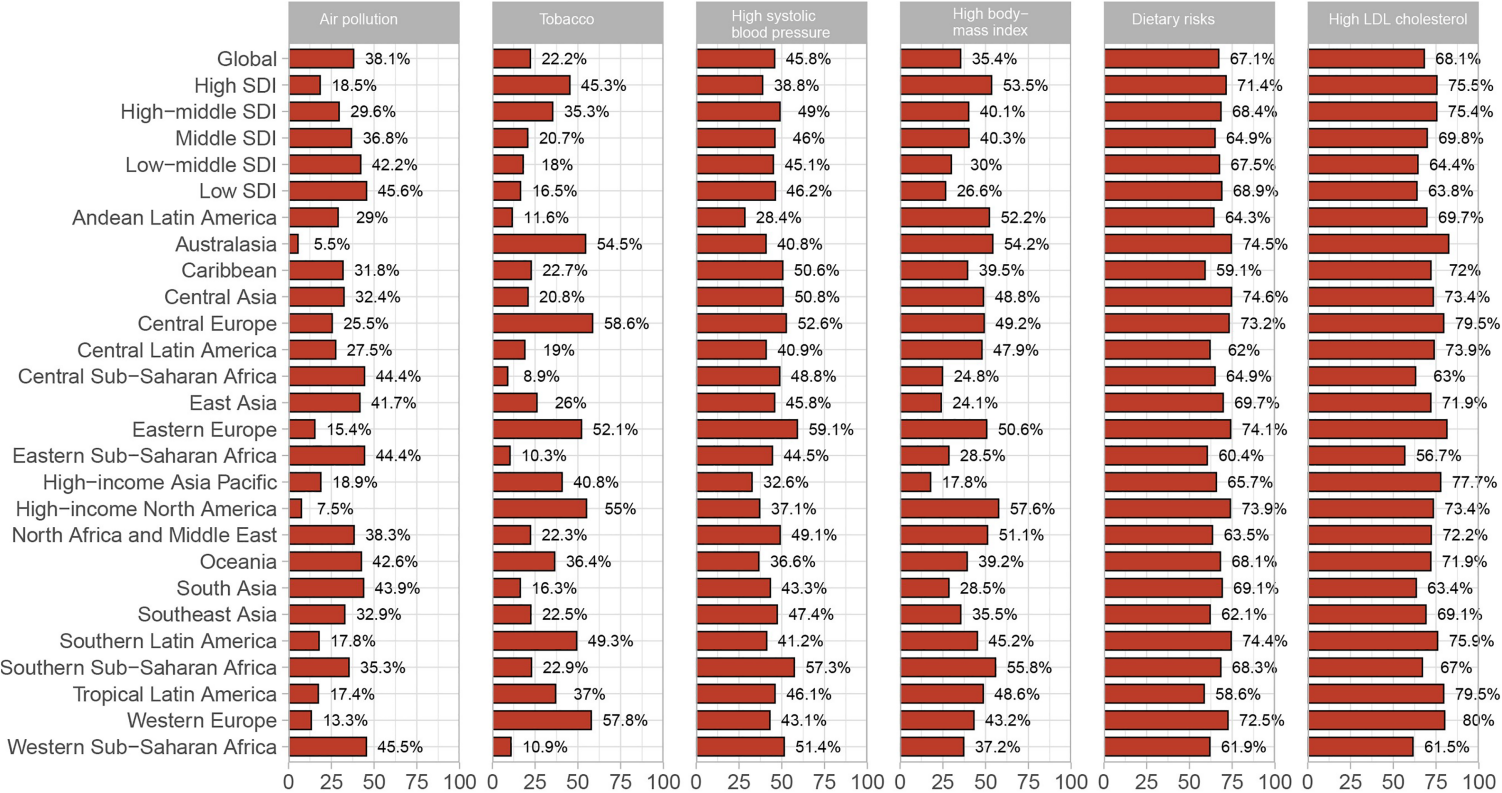
The proportion of IHD in WCBA deaths is attributable to the top six risk factors in 2019. IHD, ischemic heart disease; WCBA, women of childbearing age.

The GBD database identified twelve risk factors contributing to IHD-related deaths in WCBA in 2019. The six primary risk factors, each accounting for more than 10% of global IHD-related deaths, include high LDL cholesterol, dietary risks, high systolic blood pressure, air pollution, high body mass index, and tobacco use. Specifically, high LDL cholesterol, dietary risks, and high systolic blood pressure were attributed to 68.1%, 67.1%, and 45.8% of global IHD-related deaths in WCBA, respectively. Air pollution was responsible for 38.1% of IHD-related deaths worldwide, with the highest impact observed in Western Sub-Saharan Africa (45.5%) and the lowest in Australasia (5.2%). Globally, 35.4% of IHD-related deaths in WCBA were linked to high body mass index, with the highest percentage in High-income North America (57.6%) and the lowest in High-income Asia Pacific (17.8%). Additionally, tobacco use accounted for 22.2% of global IHD-related deaths, with the highest occurrence in Central Europe (58.6%) and the lowest in Central Sub-Saharan Africa (8.9%) (Figure 4).

## 4 Discussion

This study presents the ASPR and ASDR for IHD in WCBA across 204 countries and territories, analyzing temporal trends and uncovering disparities across socioeconomic levels, regions, and countries. A major strength of this study is the accurate estimation of age-standardized indicators for IHD in WCBA, which compensates for age structure heterogeneity and removes the confounding effects of age across different geographical areas. This finding enables valid comparisons and lays a foundation for further scientific inquiry. The findings underscore the urgency of implementing aggressive prevention measures, controlling risk factors, and enhancing healthcare coverage in various regions and countries to tackle the disease burden of IHD in WCBA. A global assessment of IHD aids governments and healthcare professionals in developing targeted prevention and management strategies.

High-income countries have experienced a long-term decline in IHD mortality over the past decades, but there are now signs that the rate of decline is slowing (19). The IHD trends in high-income countries are likely due to pharmacological treatments and improvements in healthcare, such as reduced time to hospital arrival (20). This trend highlights progress in the long-term management and treatment of IHD across different regions globally over the past several decades. However, key behavioral risk factors, such as smoking, unhealthy diets, and excessive alcohol, along with additional physiological risk factors like elevated blood pressure and cholesterol (21), may have contributed to the rising prevalence over the last two decades and the slowing down of the mortality rate decline. In addition, especially in developed countries, there has been a general increase in women's Body Mass Index (BMI), partly due to the expansion of urbanization (22). Obesity and its associated metabolic syndrome have also been linked to adverse pregnancy outcomes, such as gestational hypertension and gestational diabetes (23, 24). Another study indicates that women with diabetes are at higher risk than men with the same condition. Gender differences in healthcare access or inherent biological distinctions between men and women may explain these findings (25). Additionally, research indicates that in some developed countries and regions, especially in Western and Central Europe, the smoking rates among women greatly exceed the global average (26). Notably, in Central and Western Europe, the proportion of smoking-related IHD deaths ranked the highest and second highest among the 21 regions, at 58.6% and 57.8%, respectively. Notably, the prevalence of smoking among women aged 15–19 years is particularly high (26). Despite extensive tobacco control efforts in France, Italy, Spain, etc, women's smoking rates have remained largely unchanged and have even risen in those countries (27–29). This fact may explain the increase in the prevalence of IHD in WCBA in high SDI regions in Europe over the past 30 years. Moreover, over the last 30 years, the continual urbanization and modernization in most countries have increased exposure to harmful substances and deteriorating air quality, exacerbating the rise in IHD prevalence among WCBA globally and in various regions (30, 31). However, outside of the high SDI regions, the anticipated rise in the IHD prevalence burden has led national healthcare policymakers to prioritize primary care and the primary prevention of IHD. For instance, China, representing the middle SDI region, launched the “Healthy China 2030” initiative in 2016, aiming to improve overall population health and reduce disease incidence and mortality rates (32).

Compared to men, women are more susceptible to socio-cultural factors such as gender discrimination, socioeconomic burdens, and especially the care associated with IHD (33). Women have a higher incidence of IHD-related morbidity, experience more symptoms, frequent hospitalizations, poorer health status, and more postoperative complications (34). The disparities observed in women concerning IHD can be partly attributed to a greater prevalence of non-obstructive coronary artery disease (35). Additionally, emerging cardiac risk factors, including psychosocial elements such as stress, low socioeconomic status, and pressures related to work and marital situations, significantly influence IHD in women (36). Significant disparities in the trends of ASPR and ASDR among the 204 countries and regions at the national level indicate notable global variations in the prevention, management, and treatment of IHD in WCBA. Countries must implement gender-specific treatments and intervention measures to meet the distinct medical needs of WCBA with IHD.

The notable increase in the IHD burden within Eastern Europe could be linked to regional dietary habits. Beyond dietary risks, factors such as hypertension, smoking, and a high body mass index are key contributors to the escalating IHD burden among WCBA in this region (37). The dissolution of the former Soviet Union also emerges as a potential factor for this upward trend (38). Despite a global rise in the ASPR of IHD among WCBA, regions and countries are witnessing a declining trend. From 1990 to 2019, Australasia experienced the most significant decrease in prevalence across the 21 regions examined. The 2019 national audit of acute cardiovascular services in Australasia revealed that 82% of hospitals offer thrombolysis services, 72% utilize telemedicine for acute assessments and treatments, and 67% of patients have access to ward-based care (39). The implementation of existing cardiac rehabilitation (CR) programs, along with effective primary and secondary prevention strategies and widespread awareness of IHD and heart failure management, have contributed to this decrease (40). Nationally, Singapore has seen the most considerable reduction in ASPR. The range of initiatives undertaken by local authorities, such as tobacco control interventions, the observed decline in female smoking prevalence (41), and the significant enhancement of healthcare expenditure—including the upgrading of public hospital facilities and expansion of long-term care services (42)—offers valuable insights for policymakers in other countries.

Several limitations should be noted. Firstly, due to the imperfection of healthcare systems in underdeveloped countries, the diagnostic capacity for IHD may evolve with social and technological progress, leading to potential misdiagnosis and underdiagnosis in the GBD study. Secondly, although the GBD database contains 87 risk factors, information on other risk factors (i.e., pregnancy disorders, gestational diabetes, premature births, etc…) unique to females was limited; future studies of IHD in WCBA should include information that could facilitate such classification. Thirdly, to overcome the imbalance in data quality from a vast amount of raw data, GBD collaborators might lead to an over-reliance on the GBD study. Additionally, the absence of data on various macro and micro social, economic, political, and socio-psychological factors, particularly important for women in the GBD, is also a limitation of the study.

## 5 Conclusions

Our study findings reveal significant global disparities in the burden of morbidity and mortality due to IHD in WCBA, with a need for vigilance towards a possible future rise in ASPR in high SDI regions. Healthcare professionals and policymakers need to strengthen early detection of IHD and improve healthcare standards, and WCBA individuals should enhance their self-management awareness. Together, these efforts are essential to reduce the global prevalence and mortality rates of IHD in pregnant women before 2030 and to achieve a healthier future.

## Data Availability

The original contributions presented in the study are included in the article/Supplementary Material, further inquiries can be directed to the corresponding author.
