# Supplementary material for: Temporal trends in the prevalence and death of ischemic heart disease in women of childbearing age from 1990 to 2019: a multilevel analysis based on the Global Burden of Disease Study 2019
Source: Front Cardiovasc Med. 2024 Apr 22;11:1366832. doi: 10.3389/fcvm.2024.1366832 (PMC11070499; doi:10.3389/fcvm.2024.1366832)
Supplement: Supplementary file 1 [file Table1.docx]

**Supplementary Material**

**Table S1.** ASPR of IHD in 1990 and 2019 for WCBA in 204 countries, with AAPC from 1990 and 2019. Abbreviations: ASPR, age-standardized prevalence rate; IHD, ischemic heart disease; WCBA, women of childbearing age; UI, uncertainty interval; AAPC, average annual percent change; CI, confidence interval.

**Table S2.** AAPCs of 204 countries in ASPR of IHD in WCBA. Abbreviations: ASPR, age-standardized prevalence rate; IHD, ischemic heart disease; WCBA, women of childbearing age; AAPC, average annual percent change; CI, confidence interval.

**Table S3.** APCs of global and SDI region in ASPR of IHD in WCBA. Abbreviations: IHD, ischemic heart disease; WCBA, women of childbearing age; APC, annual percentage change; SDI, sociodemographic index; CI, confidence interval.

**Table S4.** APCs of 204 countries in ASPR of IHD in WCBA. Abbreviations: IHD, ischemic heart disease; WCBA, women of childbearing age; APC, annual percentage change; CI, confidence interval.

**Table S5.** ASDR of IHD in 1990 and 2019 for WCBA in 204 countries, with AAPC from 1990 and 2019. Abbreviations: ASDR, age-standardized death rate; IHD, ischemic heart disease; WCBA, women of childbearing age; UI, uncertainty interval; AAPC, average annual percent change; CI, confidence interval.

**Table S6.** AAPCs of 204 countries in ASDR of IHD in WCBA. Abbreviations: ASPR, age-standardized prevalence rate; IHD, ischemic heart disease; WCBA, women of childbearing age; AAPC, average annual percent change; CI, confidence interval.

**Table S7.** APCs of global and SDI region in ASDR of IHD in WCBA. Abbreviations: APC, annual percentage change; IHD, ischemic heart disease; WCBA, women of childbearing age; SDI, sociodemographic index; CI, confidence interval.

**Table S8.** APCs of 204 countries in ASDR of IHD in WCBA. Abbreviations: APC, annual percentage change; IHD, ischemic heart disease; WCBA, women of childbearing age; CI, confidence interval.

**Table S1.** ASPR of IHD in 1990 and 2019 for WCBA in 204 countries, with AAPC from 1990 and 2019. Abbreviations: ASPR, age-standardized prevalence rate; IHD, ischemic heart disease; WCBA, women of childbearing age; UI, uncertainty interval; AAPC, average annual percent change; CI, confidence interval.

| location | ASPR, per 100,000 (95% UI) | | AAPC (95% CI) |
| --- | --- | --- | --- |
|  | 1990 | 2019 |  |
| Afghanistan | 537.65 (460.53 to 625.96) | 561.84 (480.14 to 655.42) | 0.18 (0.06 to 0.3) |
| Albania | 291.3 (236.88 to 359.39) | 266.83 (219.17 to 325.67) | -0.29 (-0.32 to -0.27) |
| Algeria | 474.34 (407.41 to 550.29) | 472.09 (404.08 to 547.87) | 0.01 (-0.09 to 0.1) |
| American Samoa | 439.42 (347.77 to 552.82) | 469.27 (372.62 to 593.86) | 0.22 (0.21 to 0.24) |
| Andorra | 264.51 (226.04 to 311.18) | 275.7 (237.83 to 321.59) | 0.15 (0.11 to 0.19) |
| Angola | 252.22 (215.53 to 295.09) | 244.35 (207.52 to 286.78) | -0.11 (-0.11 to -0.11) |
| Antigua and Barbuda | 620.04 (540.59 to 712.92) | 583.24 (507.7 to 672.62) | -0.23 (-0.29 to -0.16) |
| Argentina | 138.48 (116.38 to 164.92) | 128.36 (108.39 to 152.75) | -0.25 (-0.27 to -0.23) |
| Armenia | 467.12 (396.09 to 553.21) | 444.58 (378.61 to 525.36) | -0.17 (-0.18 to -0.16) |
| Australia | 285.33 (245.95 to 332.35) | 238.73 (205.17 to 280.13) | -0.6 (-0.7 to -0.5) |
| Austria | 307.38 (262.27 to 359.36) | 306.63 (265.09 to 353.68) | -0.01 (-0.04 to 0.02) |
| Azerbaijan | 470.65 (400.73 to 558.59) | 432.26 (367.77 to 508.79) | -0.29 (-0.31 to -0.26) |
| Bahamas | 586.72 (506.92 to 677.58) | 579.42 (502.99 to 666.36) | -0.03 (-0.05 to -0.01) |
| Bahrain | 592.14 (510.77 to 685.77) | 531.73 (454.94 to 614.25) | -0.36 (-0.39 to -0.33) |
| Bangladesh | 340.28 (281.07 to 414.4) | 353.4 (292.09 to 431.45) | 0.13 (0.09 to 0.16) |
| Barbados | 623.13 (542.16 to 715.17) | 622.9 (537.63 to 716.47) | 0 (-0.02 to 0.02) |
| Belarus | 380.6 (322.16 to 452.47) | 387.35 (328.81 to 459.96) | 0.07 (0.04 to 0.11) |
| Belgium | 288.41 (243.19 to 339.34) | 309.65 (267.11 to 360.23) | 0.25 (0.2 to 0.29) |
| Belize | 608.53 (527.67 to 701.55) | 641.26 (557.25 to 740.95) | 0.19 (0.14 to 0.23) |
| Benin | 246.07 (208.15 to 289.19) | 258.04 (216.9 to 307.96) | 0.18 (0.13 to 0.24) |
| Bermuda | 601.72 (519.72 to 691.88) | 548.35 (475.7 to 628.02) | -0.35 (-0.45 to -0.24) |
| Bhutan | 360.63 (301.1 to 435.64) | 350.85 (292.06 to 423.56) | -0.09 (-0.12 to -0.06) |
| Bolivia (Plurinational State of) | 158.23 (123.76 to 202.21) | 156.38 (123.17 to 199.14) | -0.03 (-0.1 to 0.05) |
| Bosnia and Herzegovina | 363.65 (297.96 to 446.33) | 346.11 (287.64 to 418.49) | -0.16 (-0.22 to -0.09) |
| Botswana | 237.64 (201.2 to 281.79) | 248.64 (209.89 to 295.17) | 0.15 (0.12 to 0.18) |
| Brazil | 207.66 (167 to 258.29) | 197.18 (159.38 to 244.48) | -0.17 (-0.2 to -0.15) |
| Brunei Darussalam | 238.68 (203.36 to 280.41) | 245.67 (206.3 to 294.17) | 0.16 (-0.16 to 0.48) |
| Bulgaria | 323.64 (263.31 to 402.99) | 330.33 (272.36 to 402.59) | 0.09 (-0.01 to 0.19) |
| Burkina Faso | 236.15 (198.3 to 282.74) | 242.53 (201.66 to 293.64) | 0.1 (0.07 to 0.13) |
| Burundi | 260.14 (215.84 to 313.58) | 262.89 (219.27 to 318.46) | 0.04 (0.03 to 0.05) |
| Cabo Verde | 271.34 (229.88 to 319.85) | 290.36 (245.82 to 343.52) | 0.24 (0.22 to 0.25) |
| Cambodia | 270.65 (222.65 to 329.63) | 254.47 (209.64 to 311.05) | -0.19 (-0.25 to -0.13) |
| Cameroon | 200.6 (168.32 to 238.2) | 226.14 (188.81 to 269.99) | 0.42 (0.39 to 0.46) |
| Canada | 344.99 (289.65 to 403.71) | 301.67 (260.52 to 346.83) | -0.47 (-0.51 to -0.44) |
| Central African Republic | 235.4 (199.53 to 277.28) | 236.76 (200.48 to 278.36) | 0.04 (-0.04 to 0.12) |
| Chad | 237.53 (198.44 to 284.4) | 264.78 (221.24 to 319.08) | 0.39 (0.3 to 0.48) |
| Chile | 195.11 (165.38 to 230.47) | 183.01 (155.47 to 216.81) | -0.19 (-0.28 to -0.09) |
| China | 451.68 (350.53 to 579.85) | 498.26 (386.64 to 638.16) | 0.33 (0.31 to 0.35) |
| Colombia | 296.08 (246.56 to 356.43) | 265.46 (222.16 to 320.16) | -0.37 (-0.38 to -0.36) |
| Comoros | 289.89 (241.72 to 347.56) | 284.42 (238.43 to 342.16) | -0.07 (-0.08 to -0.06) |
| Congo | 261.63 (222.02 to 309.31) | 255.2 (216.51 to 300.24) | -0.07 (-0.13 to -0.01) |
| Cook Islands | 432.72 (342.32 to 545.15) | 449.24 (356.69 to 563.97) | 0.13 (0.12 to 0.14) |
| Costa Rica | 307.23 (256.5 to 368.87) | 293.28 (245.31 to 352.24) | -0.16 (-0.17 to -0.15) |
| Coted'Ivoire | 251.38 (212.66 to 297.74) | 265.93 (225.42 to 315.36) | 0.21 (0.17 to 0.26) |
| Croatia | 382.77 (306.52 to 479.18) | 329.6 (264.94 to 410.27) | -0.49 (-0.63 to -0.36) |
| Cuba | 586.43 (509.47 to 673.9) | 530.44 (461.63 to 610.37) | -0.34 (-0.38 to -0.31) |
| Cyprus | 217.42 (180.58 to 260.38) | 277.18 (227.42 to 337.25) | 1.06 (0.36 to 1.77) |
| Czechia | 469.97 (385.88 to 575.55) | 407.42 (341.03 to 491.57) | -0.49 (-0.51 to -0.47) |
| Democratic People's Republic of Korea | 366.32 (283.09 to 474.61) | 397.83 (304.93 to 511.65) | 0.29 (0.27 to 0.3) |
| Democratic Republic of the Congo | 247.33 (210.3 to 291.43) | 238.47 (201.9 to 279.89) | -0.12 (-0.14 to -0.1) |
| Denmark | 368.81 (320.2 to 427.08) | 318.62 (273.77 to 368.96) | -0.5 (-0.55 to -0.46) |
| Djibouti | 266.55 (223.01 to 321.77) | 286.11 (238.07 to 345.39) | 0.24 (0.22 to 0.26) |
| Dominica | 574.78 (498.12 to 661.04) | 574.05 (496.36 to 663.72) | 0 (-0.01 to 0.01) |
| Dominican Republic | 518.54 (451.65 to 596.53) | 562.48 (489.52 to 648.03) | 0.28 (0.26 to 0.29) |
| Ecuador | 162.45 (126.52 to 207.11) | 170.86 (133.2 to 219.35) | 0.18 (0.1 to 0.27) |
| Egypt | 561.61 (485.79 to 648.53) | 564.13 (487.91 to 652.04) | 0.03 (-0.06 to 0.13) |
| El Salvador | 238.17 (197.54 to 287.35) | 258.87 (215.35 to 311.64) | 0.29 (0.25 to 0.33) |
| Equatorial Guinea | 249.88 (212.58 to 293.37) | 229.05 (194.61 to 269.51) | -0.3 (-0.32 to -0.27) |
| Eritrea | 239.35 (199.2 to 287.58) | 233.61 (194.12 to 281.06) | -0.09 (-0.1 to -0.07) |
| Estonia | 496.49 (425.85 to 581.96) | 494.57 (423.39 to 579.25) | -0.04 (-0.1 to 0.02) |
| Eswatini | 253.18 (212.2 to 301.92) | 264 (222.68 to 314.21) | 0.15 (0.13 to 0.16) |
| Ethiopia | 201.33 (165.44 to 246.05) | 206.71 (169.4 to 252.83) | 0.09 (0.07 to 0.12) |
| Fiji | 466.87 (370 to 591.04) | 472.76 (374.08 to 601.96) | 0.05 (0.01 to 0.09) |
| Finland | 406.75 (348.08 to 477.79) | 349.66 (301.94 to 405.34) | -0.51 (-0.55 to -0.48) |
| France | 273.88 (232.9 to 320.28) | 277.48 (238.02 to 323.82) | 0.07 (-0.04 to 0.17) |
| Gabon | 243.07 (207.12 to 284.71) | 246.79 (210.8 to 290.15) | 0.05 (0.02 to 0.08) |
| Gambia | 290.08 (245.4 to 343.29) | 316.34 (266.43 to 376.01) | 0.32 (0.25 to 0.4) |
| Georgia | 453.3 (382.8 to 537.34) | 468.14 (394.96 to 557.26) | 0.13 (0.06 to 0.21) |
| Germany | 371.59 (310.06 to 442.74) | 341.49 (294.59 to 396.41) | -0.27 (-0.33 to -0.21) |
| Ghana | 262.89 (222.99 to 311.86) | 280.93 (237.41 to 333.21) | 0.25 (0.2 to 0.3) |
| Greece | 249.1 (213.04 to 293.32) | 276.85 (237.95 to 323.19) | 0.37 (0.33 to 0.42) |
| Greenland | 301.44 (257.16 to 352.53) | 275.04 (236.01 to 317.86) | -0.32 (-0.35 to -0.28) |
| Grenada | 611.09 (529.22 to 706.83) | 623.96 (541.28 to 717.66) | 0.08 (0.05 to 0.12) |
| Guam | 377.49 (299.53 to 471.01) | 450.32 (355.84 to 567.81) | 0.61 (0.56 to 0.65) |
| Guatemala | 201.37 (166.47 to 244.15) | 229.11 (189.84 to 276.02) | 0.45 (0.42 to 0.48) |
| Guinea | 204.93 (172.76 to 243.7) | 237.7 (199.86 to 284.64) | 0.53 (0.49 to 0.56) |
| Guinea-Bissau | 260.89 (219.7 to 308.98) | 279.09 (234.02 to 330.97) | 0.26 (0.17 to 0.35) |
| Guyana | 616.18 (532.5 to 711.86) | 599.83 (517.7 to 695.84) | -0.07 (-0.12 to -0.02) |
| Haiti | 526.93 (453.7 to 611.02) | 520.21 (444.79 to 602.32) | -0.02 (-0.12 to 0.08) |
| Honduras | 277.4 (229.11 to 335.88) | 289.45 (240.03 to 352.88) | 0.16 (0.07 to 0.25) |
| Hungary | 465.67 (384.1 to 565.82) | 378.42 (314.2 to 457.19) | -0.69 (-0.74 to -0.64) |
| Iceland | 296.19 (250.02 to 346.26) | 290.99 (251.55 to 338.78) | -0.06 (-0.09 to -0.03) |
| India | 345.88 (286.93 to 420.41) | 361.34 (299.67 to 437.75) | 0.16 (0.14 to 0.17) |
| Indonesia | 199.86 (153.95 to 258.56) | 217.54 (168.07 to 279.98) | 0.31 (0.23 to 0.39) |
| Iran (Islamic Republic of) | 682.08 (576.43 to 810.27) | 650.86 (551.24 to 772) | -0.16 (-0.18 to -0.14) |
| Iraq | 606.4 (522.16 to 707.04) | 587.65 (504.75 to 681.29) | -0.09 (-0.18 to 0) |
| Ireland | 340.24 (289.27 to 398.46) | 314.4 (269.99 to 366.72) | -0.24 (-0.32 to -0.15) |
| Israel | 268.87 (225.85 to 319.92) | 271.87 (231.8 to 317.89) | 0.05 (-0.02 to 0.12) |
| Italy | 374.71 (312.54 to 447.78) | 338.14 (284.05 to 403.26) | -0.35 (-0.4 to -0.3) |
| Jamaica | 576.24 (496.82 to 665.52) | 644.14 (560.3 to 740.29) | 0.4 (0.37 to 0.42) |
| Japan | 224.81 (187.82 to 269.38) | 209.25 (175.01 to 250.2) | -0.24 (-0.27 to -0.21) |
| Jordan | 573.48 (496.66 to 666.03) | 543.12 (467.49 to 628.52) | -0.17 (-0.2 to -0.14) |
| Kazakhstan | 452.73 (382.33 to 535.94) | 401.99 (341.52 to 474.57) | -0.41 (-0.44 to -0.38) |
| Kenya | 258.91 (211.73 to 317.64) | 259.87 (212.66 to 318.54) | 0.01 (-0.02 to 0.04) |
| Kiribati | 417.68 (329 to 531.28) | 439.3 (346.74 to 555.16) | 0.19 (0.13 to 0.24) |
| Kuwait | 566.01 (484.38 to 656.23) | 576.79 (497.17 to 670.74) | 0.08 (0.02 to 0.14) |
| Kyrgyzstan | 408.63 (347.04 to 482.74) | 370.08 (312.2 to 441.57) | -0.34 (-0.36 to -0.32) |
| Lao People's Democratic Republic | 268.09 (221.28 to 327.53) | 244.61 (202.44 to 295.48) | -0.3 (-0.41 to -0.19) |
| Latvia | 376.28 (313.85 to 455.47) | 399.54 (332.85 to 485.35) | 0.21 (0.1 to 0.32) |
| Lebanon | 500.96 (432.73 to 580.61) | 528 (454.99 to 613.68) | 0.2 (0.11 to 0.29) |
| Lesotho | 199.14 (167.66 to 237.05) | 223.15 (186.66 to 266.73) | 0.38 (0.34 to 0.43) |
| Liberia | 272.69 (231.15 to 323.47) | 293.68 (245.78 to 347.74) | 0.29 (0.18 to 0.4) |
| Libya | 463.56 (397.67 to 538.6) | 516.04 (442.51 to 601.46) | 0.38 (0.31 to 0.44) |
| Lithuania | 396.65 (332.64 to 477.14) | 370.77 (310.73 to 447.22) | -0.21 (-0.33 to -0.1) |
| Luxembourg | 258.56 (217.79 to 307.76) | 264.48 (222.62 to 314.71) | 0.1 (-0.11 to 0.32) |
| Madagascar | 246.74 (206.45 to 296.44) | 268.48 (224.2 to 324.58) | 0.29 (0.28 to 0.3) |
| Malawi | 283.37 (233.44 to 346.07) | 287.8 (237.24 to 350.81) | 0.07 (0.02 to 0.12) |
| Malaysia | 360.41 (297.86 to 438.43) | 370.11 (305.77 to 450.94) | 0.11 (0.03 to 0.18) |
| Maldives | 295.74 (243.26 to 361.3) | 273.29 (226.33 to 332.97) | -0.26 (-0.31 to -0.21) |
| Mali | 215.42 (182.61 to 252.95) | 223.22 (188.48 to 263.43) | 0.14 (0.08 to 0.2) |
| Malta | 253.39 (213.69 to 298.72) | 243.26 (205.82 to 288.57) | -0.1 (-0.35 to 0.16) |
| Marshall Islands | 377.33 (298.21 to 474.62) | 393.67 (311.82 to 498.8) | 0.15 (0.13 to 0.17) |
| Mauritania | 272.69 (231.27 to 321.99) | 281.98 (238.85 to 334.17) | 0.13 (0.07 to 0.18) |
| Mauritius | 311.9 (258.79 to 376.01) | 284.41 (235.57 to 344.12) | -0.31 (-0.35 to -0.26) |
| Mexico | 370.53 (307.99 to 448.26) | 314.32 (260.5 to 379.74) | -0.57 (-0.63 to -0.5) |
| Micronesia (Federated States of) | 395.58 (314.5 to 501.33) | 374.32 (296.05 to 475.84) | -0.19 (-0.22 to -0.16) |
| Monaco | 283.61 (240.95 to 334.04) | 277.67 (238.12 to 325.29) | -0.06 (-0.1 to -0.01) |
| Mongolia | 441.85 (373.92 to 527.15) | 441.64 (374.49 to 522.13) | 0.02 (-0.06 to 0.11) |
| Montenegro | 405.09 (331.12 to 501.59) | 372.77 (307.11 to 455.51) | -0.27 (-0.31 to -0.24) |
| Morocco | 558.51 (477.12 to 656.77) | 539.22 (462.46 to 632.37) | -0.11 (-0.17 to -0.05) |
| Mozambique | 279.57 (230.89 to 338.18) | 291.69 (242.7 to 351.68) | 0.17 (0.09 to 0.24) |
| Myanmar | 276.37 (230.53 to 332.62) | 247.99 (206.85 to 298.04) | -0.34 (-0.44 to -0.24) |
| Namibia | 259.33 (218.46 to 306.95) | 243.34 (204.93 to 287.98) | -0.22 (-0.27 to -0.18) |
| Nauru | 404.61 (320.47 to 511.63) | 417.94 (330.21 to 527.89) | 0.12 (0.09 to 0.15) |
| Nepal | 338.72 (281.64 to 410.56) | 310.91 (259.44 to 374.76) | -0.3 (-0.32 to -0.27) |
| Netherlands | 382.41 (327.15 to 445.68) | 372.87 (321.41 to 431.16) | -0.09 (-0.12 to -0.07) |
| New Zealand | 322.79 (267.8 to 388.61) | 266.78 (221.53 to 321.27) | -0.64 (-0.71 to -0.56) |
| Nicaragua | 295.6 (247.03 to 354.66) | 300.83 (250.18 to 363.87) | 0.06 (0.05 to 0.07) |
| Niger | 232.3 (196.14 to 275.57) | 243.62 (205.09 to 288.79) | 0.18 (0.14 to 0.22) |
| Nigeria | 209.69 (174.46 to 253.01) | 236.65 (196.94 to 286.43) | 0.43 (0.37 to 0.48) |
| Niue | 426.59 (338.93 to 540) | 438.99 (348.7 to 554.46) | 0.1 (0.08 to 0.12) |
| North Macedonia | 338.96 (275.18 to 417.8) | 313.86 (258.61 to 383.38) | -0.26 (-0.3 to -0.23) |
| Northern Mariana Islands | 379.72 (301.46 to 475.11) | 423.38 (335.19 to 533.61) | 0.38 (0.36 to 0.39) |
| Norway | 325.72 (271.45 to 390.38) | 298.37 (250.96 to 354.98) | -0.28 (-0.36 to -0.2) |
| Oman | 512.17 (442.77 to 593.45) | 581.26 (499.91 to 677.24) | 0.46 (0.37 to 0.55) |
| Pakistan | 424.17 (350.8 to 513.97) | 444.26 (366.91 to 538.84) | 0.17 (0.14 to 0.2) |
| Palau | 414.5 (327.19 to 525.27) | 428.83 (337.69 to 543.55) | 0.12 (0.1 to 0.14) |
| Palestine | 544.89 (468.14 to 635.73) | 548.27 (471.07 to 637.11) | 0.03 (-0.01 to 0.07) |
| Panama | 267.52 (222.95 to 322.74) | 274.65 (228.68 to 330.13) | 0.09 (0.08 to 0.1) |
| Papua New Guinea | 400.87 (318.2 to 507.24) | 416.81 (327.55 to 524.76) | 0.15 (0.12 to 0.17) |
| Paraguay | 193 (156.37 to 239.66) | 205.74 (167.7 to 254.54) | 0.22 (0.21 to 0.24) |
| Peru | 139.34 (108.97 to 177.67) | 137.29 (106.69 to 175.49) | -0.04 (-0.09 to 0) |
| Philippines | 399.53 (335.75 to 477.37) | 454.36 (381.98 to 543.61) | 0.45 (0.41 to 0.48) |
| Poland | 309.35 (240.88 to 399.59) | 287.31 (227.18 to 363.11) | -0.25 (-0.34 to -0.16) |
| Portugal | 205.73 (166.96 to 254.2) | 240.78 (195.23 to 294.3) | 0.61 (0.37 to 0.84) |
| Puerto Rico | 647.18 (561.35 to 743.6) | 650.28 (565.73 to 748.56) | 0.01 (-0.01 to 0.03) |
| Qatar | 473.03 (405.99 to 548.35) | 437.27 (378.45 to 504.43) | -0.27 (-0.3 to -0.25) |
| Republic of Korea | 167.66 (141.65 to 199.12) | 171.06 (142.49 to 206.47) | 0.14 (-0.05 to 0.33) |
| Republic of Moldova | 361.82 (305.95 to 431.2) | 390.68 (331.2 to 465.37) | 0.27 (0.23 to 0.32) |
| Romania | 341.94 (278.11 to 423) | 333.33 (275.44 to 406.73) | -0.07 (-0.24 to 0.11) |
| Russian Federation | 397.3 (332.25 to 477.29) | 440.11 (367.82 to 528.97) | 0.4 (0.18 to 0.62) |
| Rwanda | 212.38 (176.27 to 257.05) | 206.91 (171.05 to 250.18) | -0.1 (-0.14 to -0.06) |
| Saint Kitts and Nevis | 589.35 (509.49 to 680.29) | 591.12 (511.98 to 682.4) | 0.02 (-0.01 to 0.04) |
| Saint Lucia | 647.57 (561.78 to 743.97) | 636.66 (554.81 to 734.58) | -0.06 (-0.07 to -0.05) |
| Saint Vincent and the Grenadines | 589.44 (511.44 to 679.62) | 580.53 (503.57 to 668.05) | -0.05 (-0.08 to -0.02) |
| Samoa | 453.89 (358.54 to 573.07) | 450.96 (357.24 to 575.98) | -0.01 (-0.03 to 0.01) |
| San Marino | 289.25 (246.91 to 340.37) | 282.86 (242.38 to 330.18) | -0.06 (-0.12 to -0.01) |
| Sao Tome and Principe | 268.35 (227.38 to 317.04) | 291 (246.47 to 343.06) | 0.29 (0.24 to 0.34) |
| Saudi Arabia | 462.66 (400.4 to 535.8) | 518.1 (445.13 to 600.39) | 0.4 (0.35 to 0.45) |
| Senegal | 291.64 (247.53 to 343.92) | 292.94 (245.95 to 346.53) | 0.03 (0 to 0.06) |
| Serbia | 423.6 (353.98 to 512.94) | 445.36 (373.62 to 532.92) | 0.17 (0.13 to 0.2) |
| Seychelles | 304.21 (253.08 to 367.34) | 290.19 (239.5 to 353.12) | -0.15 (-0.19 to -0.1) |
| Sierra Leone | 300.63 (254.85 to 356.37) | 305.1 (255.96 to 364.03) | 0.08 (-0.01 to 0.16) |
| Singapore | 288.79 (248.15 to 334.86) | 219.12 (189.9 to 251.81) | -0.95 (-0.99 to -0.9) |
| Slovakia | 399.98 (326.04 to 495.32) | 309.49 (254.28 to 376.75) | -0.88 (-0.91 to -0.85) |
| Slovenia | 412.48 (333.76 to 514.51) | 336.88 (274.37 to 412.76) | -0.69 (-0.72 to -0.66) |
| Solomon Islands | 386.35 (306.13 to 491.87) | 387.7 (305.74 to 488.59) | 0.02 (-0.03 to 0.07) |
| Somalia | 251.3 (209.62 to 302.8) | 265.94 (219.83 to 322.67) | 0.2 (0.18 to 0.21) |
| South Africa | 309.45 (257.9 to 371.88) | 265.72 (221.51 to 320.16) | -0.52 (-0.58 to -0.45) |
| South Sudan | 246.99 (206.85 to 298.15) | 276.41 (230.98 to 333.55) | 0.38 (0.35 to 0.41) |
| Spain | 294.81 (250.73 to 347.31) | 302.51 (259.9 to 351.85) | 0.1 (-0.02 to 0.22) |
| Sri Lanka | 302.84 (250.91 to 365.44) | 290.98 (241.01 to 351.32) | -0.13 (-0.17 to -0.1) |
| Sudan | 485.29 (417.5 to 564.94) | 525.92 (451.6 to 613.12) | 0.3 (0.19 to 0.41) |
| Suriname | 589.16 (510.09 to 679.23) | 654.97 (566.74 to 755.37) | 0.38 (0.31 to 0.45) |
| Sweden | 338.65 (281.96 to 406.64) | 370.25 (310.87 to 444.36) | 0.32 (0.26 to 0.37) |
| Switzerland | 260.47 (221.25 to 304.76) | 255.18 (218.48 to 298.36) | -0.06 (-0.12 to 0.01) |
| Syrian Arab Republic | 483.62 (416.81 to 562.7) | 517.73 (446.76 to 599.81) | 0.27 (0.13 to 0.4) |
| Taiwan (Province of China) | 449.4 (345.7 to 581.98) | 422.88 (346.26 to 516.13) | -0.2 (-0.27 to -0.14) |
| Tajikistan | 425.57 (360.9 to 508.11) | 407.3 (345 to 481.03) | -0.13 (-0.2 to -0.07) |
| Thailand | 244.52 (201.44 to 294.15) | 242.48 (200.48 to 292.94) | -0.01 (-0.07 to 0.04) |
| Timor-Leste | 257.1 (212.23 to 314.08) | 261.09 (214.55 to 318.56) | 0.08 (-0.05 to 0.21) |
| Togo | 263.01 (221.39 to 312.05) | 280.61 (236.78 to 333.54) | 0.25 (0.16 to 0.33) |
| Tokelau | 414.31 (327.07 to 524.48) | 428.09 (336.34 to 544.79) | 0.12 (0.1 to 0.14) |
| Tonga | 469.72 (371.24 to 595.31) | 478.84 (377.73 to 606.73) | 0.07 (0.03 to 0.1) |
| Trinidad and Tobago | 761.2 (660.83 to 878.87) | 730.15 (633.96 to 840.13) | -0.12 (-0.2 to -0.05) |
| Tunisia | 451.64 (387.31 to 525.16) | 464.24 (397.26 to 536.86) | 0.1 (0.07 to 0.13) |
| Turkey | 463.84 (393.23 to 547.17) | 369.21 (314.57 to 434.88) | -0.77 (-0.83 to -0.71) |
| Turkmenistan | 416.9 (352.86 to 492.95) | 423.21 (358.19 to 499.54) | 0.06 (-0.01 to 0.13) |
| Tuvalu | 409.02 (323.41 to 521.55) | 423.66 (334.09 to 536.06) | 0.13 (0.03 to 0.22) |
| Uganda | 208.26 (172.89 to 250.69) | 210.2 (174.46 to 254.5) | 0.03 (0.01 to 0.05) |
| Ukraine | 428.06 (356.53 to 517.02) | 457.69 (378.64 to 554.86) | 0.26 (0.17 to 0.35) |
| United Arab Emirates | 482.5 (414.56 to 559.7) | 498.3 (429 to 578.25) | 0.11 (0.09 to 0.14) |
| United Kingdom | 315.73 (264.69 to 376.35) | 292.98 (248.1 to 346.7) | -0.25 (-0.36 to -0.15) |
| United Republic of Tanzania | 221.51 (185.18 to 265.62) | 254.18 (212.72 to 306) | 0.48 (0.45 to 0.51) |
| United States of America | 336.98 (281.75 to 404.88) | 339.81 (286.1 to 402.63) | 0.03 (-0.08 to 0.14) |
| United States Virgin Islands | 578.78 (504.17 to 664.04) | 579.15 (502.93 to 664.73) | 0 (-0.01 to 0.01) |
| Uruguay | 197.51 (167.39 to 232.66) | 177.31 (150.43 to 209.8) | -0.35 (-0.47 to -0.23) |
| Uzbekistan | 376.21 (319.5 to 444.89) | 393.55 (333.46 to 462.89) | 0.16 (0.12 to 0.2) |
| Vanuatu | 490.66 (388.68 to 621.61) | 483.58 (380.17 to 615.26) | -0.04 (-0.09 to 0) |
| Venezuela (Bolivarian Republic of) | 343.51 (285.79 to 413.09) | 304.09 (255.06 to 366.88) | -0.4 (-0.5 to -0.3) |
| Viet Nam | 286.7 (236.44 to 350.13) | 282.61 (233.14 to 343.43) | -0.04 (-0.1 to 0.02) |
| Yemen | 484.61 (417.35 to 562.84) | 506.64 (436.52 to 589.58) | 0.18 (0.06 to 0.3) |
| Zambia | 218.27 (183.13 to 261.98) | 221.78 (184.78 to 265.95) | 0.05 (0.03 to 0.08) |
| Zimbabwe | 234.86 (198.21 to 277.8) | 255.06 (214.94 to 301.99) | 0.28 (0.27 to 0.3) |

**Table S2.** AAPCs of 204 countries in ASPR of IHD in WCBA. Abbreviations: ASPR, age-standardized prevalence rate; IHD, ischemic heart disease; WCBA, women of childbearing age; AAPC, average annual percent change; CI, confidence interval.

| In ASPR | AAPC (95% CI) |  | AAPC (95% CI) |  | AAPC (95% CI) |  |
| --- | --- | --- | --- | --- | --- | --- |
| location | 1990-1999 | p value | 2000-2009 | p value | 2010-2019 | p value |
| Afghanistan | 0.25 (0.07 to 0.42) | 0.005 | 0.28 (0.08 to 0.49) | 0.008 | -0.21 (-0.44 to 0.03) | 0.089 |
| Albania | -0.59 (-0.61 to -0.56) | 0.000 | -0.22 (-0.24 to -0.19) | 0.000 | -0.07 (-0.12 to -0.02) | 0.004 |
| Algeria | 0.04 (-0.09 to 0.17) | 0.550 | -0.03 (-0.19 to 0.13) | 0.724 | -0.13 (-0.31 to 0.05) | 0.163 |
| American Samoa | 0.55 (0.54 to 0.56) | 0.000 | 0.11 (0.09 to 0.13) | 0.000 | 0.04 (0.01 to 0.07) | 0.020 |
| Andorra | 0.51 (0.47 to 0.56) | 0.000 | -0.43 (-0.52 to -0.34) | 0.000 | 0.35 (0.27 to 0.42) | 0.000 |
| Angola | 0.04 (0.03 to 0.04) | 0.000 | -0.22 (-0.22 to -0.21) | 0.000 | -0.17 (-0.18 to -0.16) | 0.000 |
| Antigua and Barbuda | -0.25 (-0.4 to -0.1) | 0.001 | -0.32 (-0.4 to -0.23) | 0.000 | -0.09 (-0.2 to 0.03) | 0.136 |
| Argentina | -0.31 (-0.34 to -0.29) | 0.000 | 0.07 (0.04 to 0.1) | 0.000 | -0.58 (-0.63 to -0.53) | 0.000 |
| Armenia | -0.32 (-0.33 to -0.3) | 0.000 | -0.22 (-0.23 to -0.21) | 0.000 | 0.04 (0.02 to 0.05) | 0.000 |
| Australia | -0.98 (-1.1 to -0.87) | 0.000 | -0.62 (-0.71 to -0.54) | 0.000 | -0.18 (-0.45 to 0.1) | 0.209 |
| Austria | -0.12 (-0.15 to -0.1) | 0.000 | -0.01 (-0.03 to 0) | 0.081 | 0.12 (0.04 to 0.19) | 0.004 |
| Azerbaijan | -0.44 (-0.48 to -0.41) | 0.000 | -0.21 (-0.26 to -0.17) | 0.000 | -0.24 (-0.29 to -0.2) | 0.000 |
| Bahamas | -0.34 (-0.37 to -0.31) | 0.000 | 0.15 (0.12 to 0.19) | 0.000 | 0.07 (0.03 to 0.1) | 0.000 |
| Bahrain | -0.44 (-0.49 to -0.39) | 0.000 | -0.51 (-0.57 to -0.46) | 0.000 | -0.18 (-0.24 to -0.13) | 0.000 |
| Bangladesh | 0.21 (0.12 to 0.3) | 0.000 | 0.08 (0.07 to 0.09) | 0.000 | 0.11 (0.04 to 0.18) | 0.002 |
| Barbados | 0.16 (0.13 to 0.18) | 0.000 | -0.18 (-0.2 to -0.15) | 0.000 | -0.01 (-0.05 to 0.04) | 0.707 |
| Belarus | 0.58 (0.53 to 0.64) | 0.000 | -0.02 (-0.08 to 0.05) | 0.607 | -0.49 (-0.55 to -0.43) | 0.000 |
| Belgium | 0.28 (0.26 to 0.3) | 0.000 | 0.47 (0.4 to 0.54) | 0.000 | -0.07 (-0.19 to 0.05) | 0.269 |
| Belize | 0.38 (0.32 to 0.45) | 0.000 | 0.19 (0.11 to 0.26) | 0.000 | -0.09 (-0.18 to 0) | 0.039 |
| Benin | 0.33 (0.25 to 0.41) | 0.000 | 0.04 (-0.06 to 0.14) | 0.404 | 0.1 (-0.02 to 0.21) | 0.098 |
| Bermuda | -0.29 (-0.45 to -0.13) | 0.000 | -0.37 (-0.55 to -0.18) | 0.000 | -0.27 (-0.48 to -0.05) | 0.014 |
| Bhutan | 0.06 (0.03 to 0.09) | 0.000 | -0.13 (-0.14 to -0.12) | 0.000 | -0.24 (-0.32 to -0.16) | 0.000 |
| Bolivia (Plurinational State of) | -0.18 (-0.29 to -0.07) | 0.001 | 0.08 (-0.06 to 0.21) | 0.257 | -0.05 (-0.2 to 0.1) | 0.533 |
| Bosnia and Herzegovina | -0.43 (-0.49 to -0.37) | 0.000 | 0.07 (-0.05 to 0.18) | 0.240 | -0.17 (-0.32 to -0.01) | 0.033 |
| Botswana | 0.38 (0.34 to 0.42) | 0.000 | 0.34 (0.27 to 0.41) | 0.000 | -0.28 (-0.3 to -0.26) | 0.000 |
| Brazil | -0.07 (-0.1 to -0.04) | 0.000 | -0.24 (-0.29 to -0.19) | 0.000 | -0.24 (-0.28 to -0.19) | 0.000 |
| Brunei Darussalam | 0.01 (-0.45 to 0.47) | 0.970 | 0.24 (-0.31 to 0.79) | 0.393 | -0.09 (-0.74 to 0.56) | 0.785 |
| Bulgaria | 0.4 (0.2 to 0.59) | 0.000 | -0.16 (-0.3 to -0.02) | 0.025 | 0.06 (-0.09 to 0.2) | 0.430 |
| Burkina Faso | 0.68 (0.63 to 0.74) | 0.000 | -0.39 (-0.45 to -0.33) | 0.000 | -0.05 (-0.08 to -0.02) | 0.003 |
| Burundi | 0.34 (0.33 to 0.36) | 0.000 | -0.09 (-0.11 to -0.07) | 0.000 | -0.2 (-0.22 to -0.18) | 0.000 |
| Cabo Verde | 0.73 (0.7 to 0.76) | 0.000 | 0.05 (0.04 to 0.07) | 0.000 | -0.13 (-0.15 to -0.12) | 0.000 |
| Cambodia | -0.09 (-0.18 to 0) | 0.063 | -0.52 (-0.62 to -0.41) | 0.000 | -0.09 (-0.2 to 0.02) | 0.092 |
| Cameroon | 0.84 (0.79 to 0.88) | 0.000 | 0.49 (0.41 to 0.58) | 0.000 | -0.18 (-0.23 to -0.13) | 0.000 |
| Canada | -0.93 (-0.99 to -0.88) | 0.000 | -0.37 (-0.43 to -0.31) | 0.000 | 0.03 (-0.03 to 0.09) | 0.345 |
| Central African Republic | -0.03 (-0.15 to 0.08) | 0.581 | 0.08 (-0.06 to 0.22) | 0.288 | -0.02 (-0.19 to 0.14) | 0.777 |
| Chad | 0.97 (0.73 to 1.2) | 0.000 | 0.09 (-0.02 to 0.21) | 0.104 | 0.14 (0.09 to 0.2) | 0.000 |
| Chile | -0.53 (-0.57 to -0.49) | 0.000 | -0.09 (-0.22 to 0.04) | 0.194 | 0.03 (-0.21 to 0.27) | 0.821 |
| China | 0.65 (0.63 to 0.67) | 0.000 | 0.05 (0 to 0.1) | 0.059 | 0.3 (0.25 to 0.35) | 0.000 |
| Colombia | -0.52 (-0.53 to -0.51) | 0.000 | -0.42 (-0.44 to -0.4) | 0.000 | -0.19 (-0.21 to -0.18) | 0.000 |
| Comoros | 0.02 (0.01 to 0.04) | 0.011 | -0.22 (-0.24 to -0.2) | 0.000 | -0.03 (-0.06 to 0) | 0.050 |
| Congo | -0.27 (-0.31 to -0.23) | 0.000 | 0.06 (-0.06 to 0.19) | 0.331 | -0.05 (-0.18 to 0.08) | 0.421 |
| Cook Islands | 0.43 (0.42 to 0.44) | 0.000 | 0.08 (0.07 to 0.09) | 0.000 | -0.11 (-0.13 to -0.09) | 0.000 |
| Costa Rica | 0.03 (0.03 to 0.04) | 0.000 | -0.11 (-0.11 to -0.1) | 0.000 | -0.41 (-0.44 to -0.38) | 0.000 |
| Coted'Ivoire | 0.76 (0.68 to 0.83) | 0.000 | -0.03 (-0.11 to 0.05) | 0.502 | -0.26 (-0.35 to -0.17) | 0.000 |
| Croatia | -0.28 (-0.49 to -0.08) | 0.006 | -0.89 (-1.12 to -0.66) | 0.000 | -0.43 (-0.7 to -0.16) | 0.002 |
| Cuba | -0.54 (-0.57 to -0.51) | 0.000 | -0.38 (-0.43 to -0.32) | 0.000 | -0.1 (-0.18 to -0.03) | 0.008 |
| Cyprus | 0.24 (-0.02 to 0.51) | 0.068 | 2.46 (1.35 to 3.6) | 0.000 | -0.06 (-1.7 to 1.61) | 0.943 |
| Czechia | -0.63 (-0.66 to -0.6) | 0.000 | -0.63 (-0.65 to -0.61) | 0.000 | -0.17 (-0.22 to -0.13) | 0.000 |
| Democratic People's Republic of Korea | 0.33 (0.31 to 0.36) | 0.000 | 0.56 (0.53 to 0.59) | 0.000 | -0.07 (-0.09 to -0.04) | 0.000 |
| Democratic Republic of the Congo | -0.03 (-0.06 to 0) | 0.037 | -0.28 (-0.32 to -0.25) | 0.000 | -0.08 (-0.11 to -0.04) | 0.000 |
| Denmark | -0.64 (-0.66 to -0.62) | 0.000 | -0.09 (-0.17 to -0.01) | 0.036 | -0.82 (-0.93 to -0.7) | 0.000 |
| Djibouti | 0.55 (0.53 to 0.57) | 0.000 | 0.24 (0.22 to 0.26) | 0.000 | -0.11 (-0.16 to -0.06) | 0.000 |
| Dominica | -0.11 (-0.12 to -0.11) | 0.000 | 0.19 (0.17 to 0.21) | 0.000 | -0.1 (-0.12 to -0.08) | 0.000 |
| Dominican Republic | 0.46 (0.44 to 0.49) | 0.000 | 0.42 (0.4 to 0.44) | 0.000 | -0.08 (-0.12 to -0.05) | 0.000 |
| Ecuador | -0.18 (-0.3 to -0.05) | 0.005 | 0.79 (0.65 to 0.94) | 0.000 | -0.13 (-0.3 to 0.05) | 0.151 |
| Egypt | -0.29 (-0.43 to -0.15) | 0.000 | 0.35 (0.18 to 0.51) | 0.000 | -0.12 (-0.31 to 0.07) | 0.224 |
| El Salvador | 0.35 (0.31 to 0.38) | 0.000 | 0.56 (0.48 to 0.65) | 0.000 | -0.07 (-0.13 to -0.01) | 0.023 |
| Equatorial Guinea | -0.29 (-0.32 to -0.26) | 0.000 | -0.51 (-0.52 to -0.49) | 0.000 | -0.08 (-0.16 to -0.01) | 0.020 |
| Eritrea | 0.03 (0.02 to 0.05) | 0.000 | -0.06 (-0.07 to -0.06) | 0.000 | -0.23 (-0.26 to -0.19) | 0.000 |
| Estonia | -0.07 (-0.19 to 0.04) | 0.220 | 0.26 (0.15 to 0.37) | 0.000 | -0.24 (-0.32 to -0.15) | 0.000 |
| Eswatini | 0.64 (0.59 to 0.68) | 0.000 | 0.25 (0.23 to 0.27) | 0.000 | -0.46 (-0.49 to -0.43) | 0.000 |
| Ethiopia | 0.25 (0.23 to 0.26) | 0.000 | 0.16 (0.14 to 0.17) | 0.000 | -0.15 (-0.23 to -0.08) | 0.000 |
| Fiji | -0.06 (-0.12 to 0) | 0.050 | 0.28 (0.21 to 0.35) | 0.000 | -0.13 (-0.21 to -0.05) | 0.001 |
| Finland | -1.69 (-1.77 to -1.61) | 0.000 | -0.01 (-0.04 to 0.02) | 0.426 | 0.22 (0.17 to 0.26) | 0.000 |
| France | -0.41 (-0.54 to -0.28) | 0.000 | 0.66 (0.52 to 0.8) | 0.000 | -0.2 (-0.45 to 0.04) | 0.098 |
| Gabon | 0.07 (0.04 to 0.09) | 0.000 | 0.18 (0.11 to 0.25) | 0.000 | -0.1 (-0.13 to -0.08) | 0.000 |
| Gambia | 0.54 (0.43 to 0.65) | 0.000 | 0.36 (0.23 to 0.49) | 0.000 | -0.11 (-0.26 to 0.04) | 0.160 |
| Georgia | 0 (-0.1 to 0.11) | 0.952 | 0.27 (0.14 to 0.4) | 0.000 | 0.04 (-0.1 to 0.19) | 0.563 |
| Germany | -0.77 (-0.88 to -0.65) | 0.000 | -0.43 (-0.48 to -0.38) | 0.000 | 0.45 (0.33 to 0.56) | 0.000 |
| Ghana | 0.51 (0.44 to 0.58) | 0.000 | 0.05 (-0.03 to 0.12) | 0.233 | -0.01 (-0.1 to 0.07) | 0.747 |
| Greece | -0.11 (-0.15 to -0.07) | 0.000 | 0.68 (0.64 to 0.72) | 0.000 | 0.47 (0.34 to 0.59) | 0.000 |
| Greenland | -0.36 (-0.45 to -0.26) | 0.000 | -0.41 (-0.45 to -0.36) | 0.000 | -0.12 (-0.14 to -0.1) | 0.000 |
| Grenada | 0.05 (-0.04 to 0.13) | 0.315 | 0.03 (0.01 to 0.04) | 0.001 | 0.19 (0.14 to 0.24) | 0.000 |
| Guam | 1.33 (1.25 to 1.4) | 0.000 | 0.6 (0.5 to 0.7) | 0.000 | -0.05 (-0.1 to 0.01) | 0.080 |
| Guatemala | 0.23 (0.19 to 0.28) | 0.000 | 0.85 (0.82 to 0.88) | 0.000 | 0.28 (0.22 to 0.34) | 0.000 |
| Guinea | 0.33 (0.28 to 0.38) | 0.000 | 0.94 (0.85 to 1.02) | 0.000 | 0.34 (0.3 to 0.37) | 0.000 |
| Guinea-Bissau | 0.48 (0.35 to 0.62) | 0.000 | 0.28 (0.11 to 0.44) | 0.001 | -0.16 (-0.34 to 0.02) | 0.088 |
| Guyana | 0.1 (0.02 to 0.17) | 0.011 | -0.22 (-0.31 to -0.13) | 0.000 | -0.21 (-0.3 to -0.12) | 0.000 |
| Haiti | 0.13 (-0.03 to 0.28) | 0.102 | -0.22 (-0.39 to -0.04) | 0.018 | -0.12 (-0.32 to 0.09) | 0.266 |
| Honduras | 0.35 (0.15 to 0.55) | 0.001 | 0.21 (0.1 to 0.33) | 0.000 | -0.11 (-0.26 to 0.04) | 0.147 |
| Hungary | -0.63 (-0.67 to -0.59) | 0.000 | -0.87 (-0.98 to -0.77) | 0.000 | -0.64 (-0.75 to -0.54) | 0.000 |
| Iceland | -0.12 (-0.16 to -0.09) | 0.000 | -0.12 (-0.19 to -0.04) | 0.002 | 0.13 (0.1 to 0.15) | 0.000 |
| India | 0.14 (0.13 to 0.16) | 0.000 | -0.29 (-0.32 to -0.27) | 0.000 | 0.6 (0.56 to 0.65) | 0.000 |
| Indonesia | 0.24 (0.14 to 0.34) | 0.000 | 0.63 (0.52 to 0.74) | 0.000 | -0.02 (-0.2 to 0.17) | 0.843 |
| Iran (Islamic Republic of) | -0.14 (-0.17 to -0.12) | 0.000 | -0.08 (-0.09 to -0.08) | 0.000 | -0.27 (-0.34 to -0.21) | 0.000 |
| Iraq | -0.14 (-0.28 to -0.01) | 0.037 | -0.19 (-0.35 to -0.03) | 0.019 | -0.07 (-0.26 to 0.11) | 0.425 |
| Ireland | -0.06 (-0.19 to 0.07) | 0.392 | -0.42 (-0.57 to -0.26) | 0.000 | -0.38 (-0.53 to -0.23) | 0.000 |
| Israel | -0.18 (-0.21 to -0.15) | 0.000 | 0.12 (0.02 to 0.22) | 0.015 | 0.19 (0.01 to 0.36) | 0.038 |
| Italy | -1.17 (-1.21 to -1.13) | 0.000 | -0.28 (-0.31 to -0.25) | 0.000 | 0.4 (0.25 to 0.54) | 0.000 |
| Jamaica | 0.34 (0.31 to 0.37) | 0.000 | 0.61 (0.6 to 0.63) | 0.000 | 0.24 (0.19 to 0.29) | 0.000 |
| Japan | -0.64 (-0.68 to -0.6) | 0.000 | 0.17 (0.12 to 0.22) | 0.000 | -0.35 (-0.43 to -0.27) | 0.000 |
| Jordan | 0.08 (0.03 to 0.13) | 0.002 | -0.45 (-0.51 to -0.39) | 0.000 | -0.24 (-0.29 to -0.18) | 0.000 |
| Kazakhstan | -0.1 (-0.16 to -0.05) | 0.000 | -0.79 (-0.82 to -0.75) | 0.000 | -0.32 (-0.35 to -0.28) | 0.000 |
| Kenya | 0.76 (0.72 to 0.8) | 0.000 | -0.59 (-0.67 to -0.52) | 0.000 | -0.24 (-0.28 to -0.19) | 0.000 |
| Kiribati | 0.35 (0.23 to 0.47) | 0.000 | 0.29 (0.26 to 0.33) | 0.000 | -0.11 (-0.23 to 0.01) | 0.062 |
| Kuwait | 0.36 (0.27 to 0.45) | 0.000 | -0.01 (-0.11 to 0.1) | 0.912 | -0.22 (-0.34 to -0.11) | 0.000 |
| Kyrgyzstan | -0.35 (-0.37 to -0.33) | 0.000 | -0.43 (-0.47 to -0.39) | 0.000 | -0.23 (-0.27 to -0.19) | 0.000 |
| Lao People's Democratic Republic | -0.73 (-0.91 to -0.56) | 0.000 | -0.21 (-0.39 to -0.02) | 0.026 | -0.1 (-0.32 to 0.12) | 0.391 |
| Latvia | 0.27 (0.23 to 0.31) | 0.000 | 0.43 (0.26 to 0.61) | 0.000 | -0.14 (-0.41 to 0.12) | 0.289 |
| Lebanon | -0.36 (-0.5 to -0.23) | 0.000 | 0.63 (0.47 to 0.79) | 0.000 | 0.3 (0.12 to 0.48) | 0.001 |
| Lesotho | 0.92 (0.9 to 0.95) | 0.000 | 0.49 (0.44 to 0.54) | 0.000 | -0.23 (-0.34 to -0.12) | 0.000 |
| Liberia | 0.15 (-0.01 to 0.3) | 0.073 | 0.46 (0.27 to 0.65) | 0.000 | 0.1 (-0.12 to 0.32) | 0.379 |
| Libya | 0.72 (0.64 to 0.79) | 0.000 | -0.02 (-0.11 to 0.07) | 0.665 | 0.42 (0.26 to 0.58) | 0.000 |
| Lithuania | -0.57 (-0.74 to -0.4) | 0.000 | -0.02 (-0.16 to 0.13) | 0.824 | -0.1 (-0.36 to 0.15) | 0.421 |
| Luxembourg | -0.32 (-0.59 to -0.05) | 0.021 | 1.05 (0.73 to 1.37) | 0.000 | -0.71 (-1.17 to -0.25) | 0.003 |
| Madagascar | 0.52 (0.52 to 0.53) | 0.000 | 0.43 (0.41 to 0.44) | 0.000 | -0.11 (-0.14 to -0.08) | 0.000 |
| Malawi | 0.67 (0.56 to 0.78) | 0.000 | -0.42 (-0.45 to -0.39) | 0.000 | -0.11 (-0.19 to -0.03) | 0.009 |
| Malaysia | -0.56 (-0.66 to -0.46) | 0.000 | 0.7 (0.55 to 0.85) | 0.000 | -0.01 (-0.16 to 0.14) | 0.898 |
| Maldives | -0.2 (-0.27 to -0.13) | 0.000 | -0.71 (-0.82 to -0.6) | 0.000 | 0.09 (0.01 to 0.16) | 0.018 |
| Mali | -0.03 (-0.12 to 0.05) | 0.450 | 0.22 (0.11 to 0.32) | 0.000 | 0.15 (0.03 to 0.27) | 0.013 |
| Malta | -0.63 (-1 to -0.26) | 0.001 | 0.38 (-0.06 to 0.82) | 0.090 | -0.33 (-0.84 to 0.17) | 0.198 |
| Marshall Islands | 0.43 (0.4 to 0.45) | 0.000 | 0.11 (0.08 to 0.14) | 0.000 | -0.11 (-0.14 to -0.08) | 0.000 |
| Mauritania | 0.4 (0.32 to 0.48) | 0.000 | 0 (-0.09 to 0.09) | 0.984 | -0.14 (-0.25 to -0.03) | 0.012 |
| Mauritius | -0.29 (-0.32 to -0.25) | 0.000 | -0.41 (-0.5 to -0.32) | 0.000 | -0.35 (-0.45 to -0.26) | 0.000 |
| Mexico | -0.81 (-0.96 to -0.66) | 0.000 | -0.29 (-0.37 to -0.21) | 0.000 | -0.68 (-0.79 to -0.56) | 0.000 |
| Micronesia (Federated States of) | -0.19 (-0.23 to -0.15) | 0.000 | -0.05 (-0.1 to 0) | 0.046 | -0.33 (-0.38 to -0.27) | 0.000 |
| Monaco | -0.35 (-0.37 to -0.32) | 0.000 | 0.03 (-0.04 to 0.1) | 0.448 | 0.12 (0.01 to 0.23) | 0.033 |
| Mongolia | 0 (-0.13 to 0.12) | 0.955 | 0.11 (-0.04 to 0.26) | 0.144 | -0.17 (-0.33 to 0) | 0.052 |
| Montenegro | 0.03 (-0.01 to 0.08) | 0.172 | -0.34 (-0.4 to -0.28) | 0.000 | -0.61 (-0.67 to -0.56) | 0.000 |
| Morocco | 0.45 (0.38 to 0.53) | 0.000 | -0.58 (-0.67 to -0.5) | 0.000 | -0.3 (-0.44 to -0.16) | 0.000 |
| Mozambique | 0.13 (0.02 to 0.24) | 0.016 | 0.26 (0.13 to 0.38) | 0.000 | 0.01 (-0.13 to 0.15) | 0.888 |
| Myanmar | -1.08 (-1.25 to -0.91) | 0.000 | 0.02 (-0.16 to 0.21) | 0.796 | -0.16 (-0.36 to 0.03) | 0.094 |
| Namibia | 0.06 (0 to 0.12) | 0.040 | -0.15 (-0.2 to -0.09) | 0.000 | -0.56 (-0.68 to -0.44) | 0.000 |
| Nauru | 0.34 (0.32 to 0.36) | 0.000 | 0.09 (0.03 to 0.15) | 0.002 | -0.12 (-0.19 to -0.06) | 0.000 |
| Nepal | -0.33 (-0.39 to -0.28) | 0.000 | 0.1 (0.07 to 0.12) | 0.000 | -0.74 (-0.78 to -0.7) | 0.000 |
| Netherlands | 0.12 (0.08 to 0.15) | 0.000 | -0.31 (-0.36 to -0.26) | 0.000 | -0.06 (-0.1 to -0.02) | 0.008 |
| New Zealand | -1.9 (-1.94 to -1.86) | 0.000 | -0.42 (-0.49 to -0.35) | 0.000 | 0.35 (0.13 to 0.58) | 0.002 |
| Nicaragua | 0.39 (0.37 to 0.42) | 0.000 | 0.04 (0.03 to 0.06) | 0.000 | -0.24 (-0.26 to -0.23) | 0.000 |
| Niger | 0.28 (0.22 to 0.34) | 0.000 | 0.07 (0 to 0.14) | 0.036 | 0.09 (0.01 to 0.16) | 0.027 |
| Nigeria | 1.05 (0.9 to 1.2) | 0.000 | 0.18 (0.11 to 0.25) | 0.000 | 0.08 (0.06 to 0.09) | 0.000 |
| Niue | 0.35 (0.32 to 0.38) | 0.000 | 0.07 (0.06 to 0.08) | 0.000 | -0.11 (-0.18 to -0.04) | 0.002 |
| North Macedonia | -0.16 (-0.17 to -0.14) | 0.000 | -0.22 (-0.3 to -0.14) | 0.000 | -0.44 (-0.5 to -0.39) | 0.000 |
| Northern Mariana Islands | 0.91 (0.89 to 0.94) | 0.000 | 0.23 (0.19 to 0.27) | 0.000 | 0.05 (0.04 to 0.06) | 0.000 |
| Norway | -0.96 (-1.02 to -0.9) | 0.000 | 0.05 (-0.11 to 0.21) | 0.555 | 0.04 (-0.14 to 0.22) | 0.661 |
| Oman | 0.07 (-0.06 to 0.2) | 0.291 | 1.15 (1.06 to 1.23) | 0.000 | 0.08 (-0.14 to 0.3) | 0.478 |
| Pakistan | -0.02 (-0.04 to 0.01) | 0.272 | 0.78 (0.71 to 0.86) | 0.000 | -0.39 (-0.44 to -0.35) | 0.000 |
| Palau | 0.41 (0.4 to 0.42) | 0.000 | 0.06 (0.04 to 0.09) | 0.000 | -0.16 (-0.22 to -0.11) | 0.000 |
| Palestine | 0.03 (-0.03 to 0.09) | 0.276 | 0.15 (0.08 to 0.22) | 0.000 | -0.2 (-0.28 to -0.12) | 0.000 |
| Panama | 0.22 (0.2 to 0.23) | 0.000 | 0.14 (0.12 to 0.16) | 0.000 | -0.08 (-0.1 to -0.07) | 0.000 |
| Papua New Guinea | 0.43 (0.39 to 0.48) | 0.000 | 0.19 (0.16 to 0.21) | 0.000 | -0.26 (-0.32 to -0.2) | 0.000 |
| Paraguay | 0.33 (0.31 to 0.36) | 0.000 | 0.46 (0.43 to 0.48) | 0.000 | -0.18 (-0.22 to -0.14) | 0.000 |
| Peru | -0.38 (-0.46 to -0.29) | 0.000 | 0.02 (-0.06 to 0.1) | 0.631 | 0.29 (0.23 to 0.35) | 0.000 |
| Philippines | 1.83 (1.78 to 1.88) | 0.000 | -0.39 (-0.46 to -0.32) | 0.000 | 0.08 (0.06 to 0.1) | 0.000 |
| Poland | -0.02 (-0.14 to 0.1) | 0.759 | 0.12 (-0.01 to 0.26) | 0.079 | -0.9 (-1.09 to -0.7) | 0.000 |
| Portugal | -0.05 (-0.39 to 0.28) | 0.758 | 1.49 (1.08 to 1.91) | 0.000 | -0.01 (-0.47 to 0.45) | 0.958 |
| Puerto Rico | 0.3 (0.26 to 0.34) | 0.000 | -0.03 (-0.06 to 0) | 0.036 | -0.22 (-0.25 to -0.19) | 0.000 |
| Qatar | 0.02 (-0.02 to 0.07) | 0.305 | -0.52 (-0.56 to -0.48) | 0.000 | -0.31 (-0.35 to -0.27) | 0.000 |
| Republic of Korea | 1.1 (0.82 to 1.38) | 0.000 | -0.21 (-0.55 to 0.13) | 0.222 | -0.81 (-1.15 to -0.47) | 0.000 |
| Republic of Moldova | 0.22 (0.2 to 0.24) | 0.000 | 0.36 (0.29 to 0.42) | 0.000 | 0.17 (0.05 to 0.3) | 0.007 |
| Romania | 0.41 (0.01 to 0.81) | 0.044 | -0.48 (-0.56 to -0.4) | 0.000 | -0.04 (-0.42 to 0.35) | 0.844 |
| Russian Federation | 0.49 (0.36 to 0.63) | 0.000 | 0.31 (-0.17 to 0.79) | 0.204 | 0.36 (-0.11 to 0.82) | 0.131 |
| Rwanda | 0.14 (0.07 to 0.21) | 0.000 | -0.39 (-0.43 to -0.36) | 0.000 | -0.01 (-0.11 to 0.09) | 0.894 |
| Saint Kitts and Nevis | 0.06 (0.02 to 0.09) | 0.001 | -0.02 (-0.05 to 0.02) | 0.444 | -0.02 (-0.06 to 0.03) | 0.471 |
| Saint Lucia | 0.07 (0.06 to 0.09) | 0.000 | -0.17 (-0.19 to -0.16) | 0.000 | -0.11 (-0.13 to -0.1) | 0.000 |
| Saint Vincent and the Grenadines | -0.29 (-0.34 to -0.25) | 0.000 | 0.18 (0.13 to 0.23) | 0.000 | -0.04 (-0.1 to 0.02) | 0.178 |
| Samoa | 0.23 (0.18 to 0.27) | 0.000 | -0.24 (-0.25 to -0.22) | 0.000 | -0.06 (-0.09 to -0.03) | 0.000 |
| San Marino | -0.3 (-0.33 to -0.28) | 0.000 | 0.02 (-0.06 to 0.1) | 0.643 | 0.06 (-0.08 to 0.2) | 0.402 |
| Sao Tome and Principe | 0.57 (0.5 to 0.64) | 0.000 | 0.24 (0.16 to 0.32) | 0.000 | -0.01 (-0.11 to 0.08) | 0.758 |
| Saudi Arabia | 0.81 (0.74 to 0.88) | 0.000 | 0.49 (0.4 to 0.58) | 0.000 | -0.24 (-0.34 to -0.14) | 0.000 |
| Senegal | 0.15 (0.11 to 0.2) | 0.000 | 0.05 (0 to 0.1) | 0.057 | -0.26 (-0.31 to -0.21) | 0.000 |
| Serbia | 0.48 (0.4 to 0.57) | 0.000 | 0.05 (0.03 to 0.07) | 0.000 | 0 (-0.07 to 0.07) | 0.921 |
| Seychelles | 0.21 (0.14 to 0.29) | 0.000 | -0.44 (-0.53 to -0.36) | 0.000 | -0.31 (-0.4 to -0.22) | 0.000 |
| Sierra Leone | 0.35 (0.23 to 0.48) | 0.000 | -0.13 (-0.28 to 0.02) | 0.093 | -0.17 (-0.34 to 0.01) | 0.059 |
| Singapore | -0.9 (-0.95 to -0.84) | 0.000 | -1.09 (-1.18 to -1.01) | 0.000 | -0.9 (-1 to -0.79) | 0.000 |
| Slovakia | -0.68 (-0.76 to -0.6) | 0.000 | -1.41 (-1.43 to -1.39) | 0.000 | -0.57 (-0.62 to -0.52) | 0.000 |
| Slovenia | -0.43 (-0.47 to -0.4) | 0.000 | -1.18 (-1.2 to -1.16) | 0.000 | -0.45 (-0.53 to -0.38) | 0.000 |
| Solomon Islands | 0.17 (0.1 to 0.24) | 0.000 | -0.08 (-0.16 to 0) | 0.056 | -0.09 (-0.18 to 0) | 0.049 |
| Somalia | 0.45 (0.43 to 0.47) | 0.000 | 0.19 (0.17 to 0.22) | 0.000 | -0.12 (-0.14 to -0.09) | 0.000 |
| South Africa | 0.12 (0.04 to 0.2) | 0.003 | -1.29 (-1.44 to -1.14) | 0.000 | -0.5 (-0.61 to -0.4) | 0.000 |
| South Sudan | 0.7 (0.67 to 0.73) | 0.000 | 0.4 (0.32 to 0.48) | 0.000 | 0.05 (0.02 to 0.08) | 0.002 |
| Spain | -0.09 (-0.22 to 0.03) | 0.148 | 0.19 (-0.1 to 0.47) | 0.202 | 0.2 (0 to 0.41) | 0.055 |
| Sri Lanka | -0.48 (-0.54 to -0.42) | 0.000 | 0.34 (0.27 to 0.42) | 0.000 | -0.42 (-0.49 to -0.35) | 0.000 |
| Sudan | 0.42 (0.26 to 0.58) | 0.000 | 0.29 (0.1 to 0.49) | 0.003 | 0.06 (-0.16 to 0.28) | 0.593 |
| Suriname | 0.6 (0.5 to 0.7) | 0.000 | 0.4 (0.28 to 0.51) | 0.000 | 0.04 (-0.09 to 0.18) | 0.512 |
| Sweden | -0.19 (-0.24 to -0.14) | 0.000 | 0.79 (0.63 to 0.95) | 0.000 | 0.4 (0.35 to 0.45) | 0.000 |
| Switzerland | -0.4 (-0.49 to -0.31) | 0.000 | 0.26 (0.16 to 0.35) | 0.000 | -0.1 (-0.26 to 0.06) | 0.234 |
| Syrian Arab Republic | 0.32 (0.13 to 0.52) | 0.001 | 0.16 (-0.07 to 0.4) | 0.175 | 0.17 (-0.11 to 0.44) | 0.229 |
| Taiwan (Province of China) | 0.02 (-0.14 to 0.18) | 0.816 | 0.51 (0.43 to 0.58) | 0.000 | -1.27 (-1.36 to -1.18) | 0.000 |
| Tajikistan | -0.18 (-0.23 to -0.13) | 0.000 | -0.31 (-0.44 to -0.18) | 0.000 | 0.09 (-0.06 to 0.24) | 0.229 |
| Thailand | 0.06 (-0.01 to 0.13) | 0.113 | -0.43 (-0.5 to -0.37) | 0.000 | 0.25 (0.1 to 0.41) | 0.001 |
| Timor-Leste | -0.06 (-0.25 to 0.13) | 0.518 | 0.06 (-0.17 to 0.29) | 0.614 | 0.17 (-0.09 to 0.42) | 0.203 |
| Togo | 0.55 (0.43 to 0.67) | 0.000 | 0.13 (-0.02 to 0.28) | 0.092 | -0.08 (-0.25 to 0.09) | 0.352 |
| Tokelau | 0.38 (0.35 to 0.41) | 0.000 | 0.08 (0.05 to 0.11) | 0.000 | -0.16 (-0.2 to -0.12) | 0.000 |
| Tonga | 0.38 (0.32 to 0.44) | 0.000 | 0.01 (-0.06 to 0.09) | 0.712 | -0.23 (-0.26 to -0.2) | 0.000 |
| Trinidad and Tobago | 0.04 (-0.07 to 0.14) | 0.486 | -0.39 (-0.52 to -0.27) | 0.000 | -0.13 (-0.27 to 0.01) | 0.066 |
| Tunisia | 0.18 (0.13 to 0.23) | 0.000 | 0.07 (0.02 to 0.13) | 0.008 | 0.01 (-0.05 to 0.07) | 0.672 |
| Turkey | -0.15 (-0.24 to -0.06) | 0.002 | -0.9 (-1.01 to -0.78) | 0.000 | -1.51 (-1.62 to -1.4) | 0.000 |
| Turkmenistan | -0.14 (-0.24 to -0.03) | 0.009 | 0.13 (0.03 to 0.23) | 0.013 | 0.17 (0.03 to 0.32) | 0.021 |
| Tuvalu | 0.39 (0.26 to 0.53) | 0.000 | 0.1 (0.06 to 0.14) | 0.000 | -0.11 (-0.37 to 0.16) | 0.417 |
| Uganda | 0.39 (0.36 to 0.42) | 0.000 | -0.13 (-0.16 to -0.11) | 0.000 | -0.19 (-0.25 to -0.13) | 0.000 |
| Ukraine | 0.56 (0.39 to 0.73) | 0.000 | -0.01 (-0.18 to 0.16) | 0.919 | 0.19 (0.06 to 0.31) | 0.003 |
| United Arab Emirates | -0.08 (-0.09 to -0.06) | 0.000 | 0.18 (0.15 to 0.21) | 0.000 | 0.2 (0.12 to 0.27) | 0.000 |
| United Kingdom | -0.87 (-1.01 to -0.73) | 0.000 | -0.36 (-0.4 to -0.31) | 0.000 | 0.49 (0.19 to 0.8) | 0.001 |
| United Republic of Tanzania | 0.48 (0.43 to 0.54) | 0.000 | 0.7 (0.64 to 0.75) | 0.000 | 0.27 (0.22 to 0.31) | 0.000 |
| United States of America | -1.98 (-2.11 to -1.85) | 0.000 | 1.41 (1.35 to 1.46) | 0.000 | 0.84 (0.52 to 1.16) | 0.000 |
| United States Virgin Islands | -0.01 (-0.02 to 0) | 0.076 | -0.06 (-0.07 to -0.04) | 0.000 | 0.1 (0.09 to 0.11) | 0.000 |
| Uruguay | -0.3 (-0.45 to -0.15) | 0.000 | -0.64 (-0.8 to -0.48) | 0.000 | -0.21 (-0.49 to 0.08) | 0.155 |
| Uzbekistan | 0.2 (0.18 to 0.22) | 0.000 | 0.15 (0.13 to 0.17) | 0.000 | 0.18 (0.07 to 0.28) | 0.001 |
| Vanuatu | -0.03 (-0.1 to 0.04) | 0.388 | 0.03 (-0.05 to 0.11) | 0.521 | -0.17 (-0.26 to -0.08) | 0.000 |
| Venezuela (Bolivarian Republic of) | -0.56 (-0.64 to -0.48) | 0.000 | -0.61 (-0.74 to -0.49) | 0.000 | 0.01 (-0.26 to 0.29) | 0.919 |
| Viet Nam | -0.65 (-0.76 to -0.54) | 0.000 | 0.65 (0.55 to 0.74) | 0.000 | -0.23 (-0.33 to -0.13) | 0.000 |
| Yemen | 0.1 (-0.07 to 0.27) | 0.255 | 0.13 (-0.08 to 0.34) | 0.218 | 0.2 (-0.04 to 0.44) | 0.099 |
| Zambia | 0.19 (0.15 to 0.23) | 0.000 | 0.01 (-0.03 to 0.06) | 0.582 | -0.11 (-0.17 to -0.06) | 0.000 |
| Zimbabwe | 0.99 (0.97 to 1.02) | 0.000 | 0.29 (0.27 to 0.32) | 0.000 | -0.45 (-0.47 to -0.42) | 0.000 |

**Table S3.** APCs of global and SDI region in ASPR of IHD in WCBA. Abbreviations: IHD, ischemic heart disease; WCBA, women of childbearing age; APC, annual percentage change; SDI, sociodemographic index; CI, confidence interval.

| Ischemic heart disease | | | |
| --- | --- | --- | --- |
| Location | Year | APC (95% CI) | p value |
| Global | 1990 - 1995 | 0.14 (0.1 to 0.18) | <0.001 |
| Global | 1995 - 2000 | 0.32 (0.26 to 0.38) | <0.001 |
| Global | 2000 - 2005 | -0.23 (-0.29 to -0.17) | <0.001 |
| Global | 2005 - 2012 | 0.33 (0.3 to 0.36) | <0.001 |
| Global | 2012 - 2017 | 0.05 (-0.01 to 0.11) | 0.089 |
| Global | 2017 - 2019 | -0.25 (-0.44 to -0.07) | 0.011 |
| High SDI | 1990 - 2000 | -0.72 (-0.79 to -0.66) | <0.001 |
| High SDI | 2000 - 2005 | 0.14 (-0.11 to 0.4) | 0.252 |
| High SDI | 2005 - 2010 | 0.86 (0.6 to 1.11) | <0.001 |
| High SDI | 2010 - 2014 | -0.21 (-0.6 to 0.19) | 0.29 |
| High SDI | 2014 - 2019 | 0.86 (0.68 to 1.03) | <0.001 |
| High-middle SDI | 1990 - 2000 | 0.51 (0.49 to 0.53) | <0.001 |
| High-middle SDI | 2000 - 2005 | -0.21 (-0.3 to -0.13) | <0.001 |
| High-middle SDI | 2005 - 2011 | 0.19 (0.13 to 0.25) | <0.001 |
| High-middle SDI | 2011 - 2019 | -0.12 (-0.15 to -0.09) | <0.001 |
| Low SDI | 1990 - 1995 | 0.07 (0.05 to 0.1) | <0.001 |
| Low SDI | 1995 - 2000 | 0.26 (0.22 to 0.3) | <0.001 |
| Low SDI | 2000 - 2005 | -0.25 (-0.29 to -0.21) | <0.001 |
| Low SDI | 2005 - 2010 | 0.56 (0.52 to 0.6) | <0.001 |
| Low SDI | 2010 - 2017 | 0.08 (0.06 to 0.1) | <0.001 |
| Low SDI | 2017 - 2019 | -0.08 (-0.21 to 0.06) | 0.233 |
| Low-middle SDI | 1990 - 1995 | 0.03 (-0.02 to 0.08) | 0.204 |
| Low-middle SDI | 1995 - 2000 | 0.29 (0.22 to 0.36) | <0.001 |
| Low-middle SDI | 2000 - 2005 | -0.41 (-0.47 to -0.34) | <0.001 |
| Low-middle SDI | 2005 - 2011 | 0.39 (0.35 to 0.44) | <0.001 |
| Low-middle SDI | 2011 - 2015 | 0.52 (0.42 to 0.63) | <0.001 |
| Low-middle SDI | 2015 - 2019 | -0.08 (-0.15 to -0.01) | 0.022 |
| Middle SDI | 1990 - 1993 | 0.24 (0.17 to 0.31) | <0.001 |
| Middle SDI | 1993 - 2000 | 0.39 (0.37 to 0.41) | <0.001 |
| Middle SDI | 2000 - 2006 | -0.22 (-0.25 to -0.19) | <0.001 |
| Middle SDI | 2006 - 2011 | 0.24 (0.19 to 0.28) | <0.001 |
| Middle SDI | 2011 - 2014 | 0.38 (0.23 to 0.52) | <0.001 |
| Middle SDI | 2014 - 2019 | -0.22 (-0.25 to -0.19) | <0.001 |

**Table S4.** APCs of 204 countries in ASPR of IHD in WCBA. Abbreviations: IHD, ischemic heart disease; WCBA, women of childbearing age; APC, annual percentage change; CI, confidence interval.

| Ischemic heart disease | | | |
| --- | --- | --- | --- |
| Location | Year | APC (95% CI) | p value |
| Afghanistan | 1990 - 1995 | -0.26 (-0.49 to -0.04) | 0.026 |
| Afghanistan | 1995 - 2000 | 0.89 (0.57 to 1.21) | 0 |
| Afghanistan | 2000 - 2005 | -0.56 (-0.88 to -0.24) | 0.002 |
| Afghanistan | 2005 - 2012 | 1.35 (1.02 to 1.68) | 0 |
| Afghanistan | 2010 - 2017 | -1.2 (-1.71 to -0.69) | 0 |
| Afghanistan | 2014 - 2019 | 0.6 (0.36 to 0.83) | 0 |
| Albania | 1990 - 2000 | -0.59 (-0.61 to -0.56) | 0 |
| Albania | 1999 - 2005 | -0.22 (-0.24 to -0.19) | 0 |
| Albania | 2009 - 2010 | -0.45 (-0.54 to -0.37) | 0 |
| Albania | 2014 - 2014 | 0.24 (0.18 to 0.3) | 0 |
| Algeria | 1990 - 2019 | -0.27 (-0.45 to -0.1) | 0.005 |
| Algeria | 1995 - 2000 | 0.44 (0.19 to 0.68) | 0.002 |
| Algeria | 2000 - 2005 | -0.67 (-0.91 to -0.42) | 0 |
| Algeria | 2005 - 2011 | 0.78 (0.53 to 1.02) | 0 |
| Algeria | 2010 - 2019 | -0.75 (-1.14 to -0.36) | 0.001 |
| Algeria | 2014 - 1995 | 0.37 (0.19 to 0.55) | 0.001 |
| American Samoa | 1990 - 2000 | 0.55 (0.54 to 0.56) | 0 |
| American Samoa | 1999 - 2005 | 0.29 (0.25 to 0.33) | 0 |
| American Samoa | 2004 - 2010 | -0.04 (-0.07 to -0.01) | 0.017 |
| American Samoa | 2010 - 2017 | 0.11 (0.08 to 0.13) | 0 |
| American Samoa | 2017 - 2019 | -0.2 (-0.33 to -0.07) | 0.005 |
| Andorra | 1990 - 1995 | 0.51 (0.47 to 0.56) | 0 |
| Andorra | 2000 - 2000 | -0.82 (-1 to -0.65) | 0 |
| Andorra | 2005 - 2005 | 0.06 (0.02 to 0.11) | 0.012 |
| Andorra | 2015 - 2011 | 0.7 (0.53 to 0.87) | 0 |
| Angola | 1990 - 2015 | 0.04 (0.03 to 0.04) | 0 |
| Angola | 2000 - 2019 | -0.3 (-0.31 to -0.29) | 0 |
| Angola | 2005 - 1993 | -0.11 (-0.12 to -0.11) | 0 |
| Angola | 2011 - 2000 | -0.38 (-0.41 to -0.34) | 0 |
| Angola | 2014 - 2006 | -0.05 (-0.06 to -0.05) | 0 |
| Antigua and Barbuda | 1990 - 2011 | -0.11 (-0.19 to -0.03) | 0.012 |
| Antigua and Barbuda | 1996 - 2014 | -0.53 (-1.01 to -0.06) | 0.032 |
| Antigua and Barbuda | 1999 - 2019 | -0.18 (-0.29 to -0.07) | 0.004 |
| Antigua and Barbuda | 2005 - 1995 | -0.49 (-0.64 to -0.34) | 0 |
| Antigua and Barbuda | 2010 - 2000 | 0.04 (-0.2 to 0.28) | 0.725 |
| Antigua and Barbuda | 2014 - 2005 | -0.19 (-0.3 to -0.08) | 0.003 |
| Argentina | 1990 - 2012 | -0.39 (-0.43 to -0.35) | 0 |
| Argentina | 1996 - 2017 | -0.16 (-0.19 to -0.12) | 0 |
| Argentina | 2004 - 2019 | 0.25 (0.2 to 0.31) | 0 |
| Argentina | 2010 - 2000 | -1.12 (-1.19 to -1.05) | 0 |
| Argentina | 2015 - 2005 | 0.1 (0.02 to 0.18) | 0.014 |
| Armenia | 1990 - 2010 | -0.52 (-0.55 to -0.48) | 0 |
| Armenia | 1993 - 2014 | -0.24 (-0.26 to -0.22) | 0 |
| Armenia | 1998 - 2019 | -0.14 (-0.15 to -0.13) | 0 |
| Armenia | 2005 - 2000 | -0.32 (-0.34 to -0.29) | 0 |
| Armenia | 2010 - 2005 | 0.06 (0.05 to 0.07) | 0 |
| Armenia | 2017 - 2011 | -0.03 (-0.09 to 0.04) | 0.399 |
| Australia | 1990 - 2019 | -1.2 (-1.46 to -0.94) | 0 |
| Australia | 1994 - 1995 | -0.81 (-0.87 to -0.75) | 0 |
| Australia | 2005 - 2000 | -0.39 (-0.58 to -0.2) | 0 |
| Australia | 2011 - 2005 | -1.16 (-1.97 to -0.34) | 0.009 |
| Australia | 2014 - 2010 | 0.46 (0.27 to 0.65) | 0 |
| Austria | 1990 - 2017 | 0.1 (0.04 to 0.16) | 0.004 |
| Austria | 1994 - 2019 | -0.3 (-0.31 to -0.28) | 0 |
| Austria | 2004 - 1995 | 0.21 (0.18 to 0.24) | 0 |
| Austria | 2011 - 2000 | -0.06 (-0.22 to 0.1) | 0.407 |
| Austria | 2014 - 2005 | 0.35 (0.19 to 0.5) | 0 |
| Austria | 2017 - 2011 | -0.01 (-0.19 to 0.17) | 0.902 |
| Azerbaijan | 1990 - 2015 | -0.65 (-0.76 to -0.55) | 0 |
| Azerbaijan | 1993 - 2019 | -0.34 (-0.37 to -0.31) | 0 |
| Azerbaijan | 2001 - 1993 | -0.45 (-0.55 to -0.34) | 0 |
| Azerbaijan | 2005 - 2000 | 0.05 (-0.01 to 0.12) | 0.115 |
| Azerbaijan | 2010 - 2006 | -0.46 (-0.56 to -0.36) | 0 |
| Azerbaijan | 2014 - 2011 | -0.07 (-0.12 to -0.02) | 0.007 |
| Bahamas | 1990 - 2014 | -0.6 (-0.65 to -0.54) | 0 |
| Bahamas | 1994 - 2019 | -0.13 (-0.16 to -0.09) | 0 |
| Bahamas | 2000 - 1995 | 0.11 (0.08 to 0.15) | 0 |
| Bahamas | 2006 - 2000 | 0.23 (0.14 to 0.31) | 0 |
| Bahamas | 2010 - 2005 | -0.14 (-0.19 to -0.09) | 0 |
| Bahamas | 2015 - 2012 | 0.32 (0.26 to 0.38) | 0 |
| Bahrain | 1990 - 2017 | -0.79 (-0.92 to -0.66) | 0 |
| Bahrain | 1993 - 2019 | -0.26 (-0.31 to -0.22) | 0 |
| Bahrain | 2000 - 2000 | -0.97 (-1.05 to -0.89) | 0 |
| Bahrain | 2005 - 2005 | 0.06 (-0.02 to 0.14) | 0.118 |
| Bahrain | 2010 - 2010 | -0.59 (-0.67 to -0.51) | 0 |
| Bahrain | 2015 - 2014 | 0.32 (0.23 to 0.41) | 0 |
| Bangladesh | 1990 - 2019 | 0.61 (0.39 to 0.83) | 0 |
| Bangladesh | 1992 - 2000 | 0.3 (0.08 to 0.52) | 0.011 |
| Bangladesh | 1995 - 2005 | -0.06 (-0.16 to 0.04) | 0.221 |
| Bangladesh | 1999 - 2011 | 0.08 (0.07 to 0.09) | 0 |
| Bangladesh | 2011 - 2019 | 0.38 (0.16 to 0.6) | 0.002 |
| Bangladesh | 2014 - 1995 | -0.05 (-0.1 to 0) | 0.061 |
| Barbados | 1990 - 2000 | -0.01 (-0.04 to 0.02) | 0.496 |
| Barbados | 1995 - 2005 | 0.37 (0.33 to 0.42) | 0 |
| Barbados | 2000 - 2010 | -0.29 (-0.33 to -0.24) | 0 |
| Barbados | 2005 - 2017 | -0.04 (-0.07 to -0.01) | 0.022 |
| Barbados | 2011 - 2019 | -0.17 (-0.31 to -0.03) | 0.023 |
| Barbados | 2014 - 1995 | 0.09 (0.06 to 0.13) | 0 |
| Belarus | 1990 - 2000 | 0.17 (0.1 to 0.24) | 0 |
| Belarus | 1995 - 2005 | 1.1 (1 to 1.2) | 0 |
| Belarus | 2000 - 2011 | -0.35 (-0.45 to -0.26) | 0 |
| Belarus | 2005 - 2015 | 0.41 (0.31 to 0.5) | 0 |
| Belarus | 2010 - 2019 | -1.08 (-1.18 to -0.99) | 0 |
| Belarus | 2015 - 1993 | 0.26 (0.16 to 0.35) | 0 |
| Belgium | 1990 - 2000 | 0.28 (0.26 to 0.3) | 0 |
| Belgium | 2005 - 2006 | 0.72 (0.55 to 0.88) | 0 |
| Belgium | 2010 - 2011 | -0.59 (-0.85 to -0.34) | 0 |
| Belgium | 2014 - 2014 | 0.35 (0.23 to 0.47) | 0 |
| Belize | 1990 - 2019 | 0.2 (0.11 to 0.28) | 0 |
| Belize | 1995 - 1995 | 0.62 (0.5 to 0.74) | 0 |
| Belize | 2000 - 2000 | -0.1 (-0.22 to 0.01) | 0.07 |
| Belize | 2005 - 2005 | 0.55 (0.43 to 0.67) | 0 |
| Belize | 2010 - 2012 | -0.47 (-0.66 to -0.28) | 0 |
| Belize | 2014 - 2017 | 0.21 (0.13 to 0.3) | 0 |
| Benin | 1990 - 2019 | -0.01 (-0.12 to 0.1) | 0.84 |
| Benin | 1995 - 2000 | 0.75 (0.6 to 0.91) | 0 |
| Benin | 2000 - 2005 | -0.2 (-0.35 to -0.05) | 0.013 |
| Benin | 2005 - 2010 | 0.35 (0.2 to 0.5) | 0 |
| Benin | 2010 - 2014 | -0.08 (-0.33 to 0.16) | 0.468 |
| Benin | 2014 - 2019 | 0.24 (0.13 to 0.35) | 0.001 |
| Bermuda | 1990 - 2000 | 0.12 (-0.1 to 0.33) | 0.258 |
| Bermuda | 1995 - 2005 | -0.8 (-1.09 to -0.51) | 0 |
| Bermuda | 2000 - 2011 | 0.04 (-0.25 to 0.33) | 0.797 |
| Bermuda | 2005 - 2019 | -0.87 (-1.15 to -0.59) | 0 |
| Bermuda | 2010 - 1995 | 0.12 (-0.34 to 0.58) | 0.584 |
| Bermuda | 2014 - 2000 | -0.57 (-0.78 to -0.37) | 0 |
| Bhutan | 1990 - 2005 | -0.16 (-0.2 to -0.12) | 0 |
| Bhutan | 1995 - 2010 | 0.33 (0.27 to 0.39) | 0 |
| Bhutan | 2000 - 2017 | -0.13 (-0.14 to -0.12) | 0 |
| Bhutan | 2011 - 2019 | -0.48 (-0.65 to -0.31) | 0 |
| Bhutan | 2014 - 1995 | -0.24 (-0.41 to -0.06) | 0.012 |
| Bhutan | 2017 - 2000 | 0.05 (-0.13 to 0.24) | 0.542 |
| Bolivia (Plurinational State of) | 1990 - 2005 | -0.64 (-0.85 to -0.44) | 0 |
| Bolivia (Plurinational State of) | 1994 - 2011 | 0.19 (0.05 to 0.34) | 0.014 |
| Bolivia (Plurinational State of) | 2000 - 2015 | -0.2 (-0.41 to 0.01) | 0.057 |
| Bolivia (Plurinational State of) | 2005 - 2019 | 0.42 (0.22 to 0.63) | 0.001 |
| Bolivia (Plurinational State of) | 2010 - 1993 | -0.46 (-0.78 to -0.14) | 0.009 |
| Bolivia (Plurinational State of) | 2014 - 2000 | 0.28 (0.14 to 0.43) | 0.001 |
| Bosnia and Herzegovina | 1990 - 2006 | -0.43 (-0.49 to -0.37) | 0 |
| Bosnia and Herzegovina | 1999 - 2011 | -0.2 (-0.34 to -0.05) | 0.01 |
| Bosnia and Herzegovina | 2005 - 2014 | 0.4 (0.19 to 0.61) | 0.001 |
| Bosnia and Herzegovina | 2010 - 2019 | -0.82 (-1.14 to -0.49) | 0 |
| Bosnia and Herzegovina | 2014 - 1995 | 0.36 (0.21 to 0.5) | 0 |
| Botswana | 1990 - 2000 | 0.62 (0.51 to 0.73) | 0 |
| Botswana | 1993 - 2005 | 0.26 (0.23 to 0.29) | 0 |
| Botswana | 2001 - 2012 | 0.61 (0.4 to 0.83) | 0 |
| Botswana | 2004 - 2017 | 0.19 (0.14 to 0.23) | 0 |
| Botswana | 2010 - 2019 | -0.28 (-0.3 to -0.26) | 0 |
| Brazil | 1990 - 2000 | 0.09 (0.06 to 0.13) | 0 |
| Brazil | 1996 - 2005 | -0.39 (-0.45 to -0.33) | 0 |
| Brazil | 2001 - 2010 | -0.69 (-0.79 to -0.59) | 0 |
| Brazil | 2005 - 2014 | 0.25 (0.18 to 0.31) | 0 |
| Brazil | 2010 - 2019 | -0.62 (-0.68 to -0.56) | 0 |
| Brazil | 2015 - 2000 | 0.25 (0.18 to 0.31) | 0 |
| Brunei Darussalam | 1990 - 2005 | -0.68 (-1.29 to -0.07) | 0.032 |
| Brunei Darussalam | 1995 - 2011 | 0.88 (0.02 to 1.74) | 0.046 |
| Brunei Darussalam | 2000 - 2019 | -1.38 (-2.21 to -0.55) | 0.003 |
| Brunei Darussalam | 2005 - 1995 | 2.31 (1.41 to 3.21) | 0 |
| Brunei Darussalam | 2010 - 2000 | -2.26 (-3.62 to -0.87) | 0.004 |
| Brunei Darussalam | 2014 - 2005 | 1.68 (1.02 to 2.33) | 0 |
| Bulgaria | 1990 - 2010 | 0.21 (-0.04 to 0.47) | 0.099 |
| Bulgaria | 1995 - 2017 | 0.62 (0.28 to 0.97) | 0.002 |
| Bulgaria | 2000 - 2019 | 0.01 (-0.18 to 0.2) | 0.936 |
| Bulgaria | 2007 - 1995 | -0.74 (-0.93 to -0.56) | 0 |
| Bulgaria | 2014 - 2000 | 0.7 (0.46 to 0.95) | 0 |
| Burkina Faso | 1990 - 2005 | 0.21 (0.14 to 0.28) | 0 |
| Burkina Faso | 1995 - 2011 | 1.28 (1.19 to 1.38) | 0 |
| Burkina Faso | 2000 - 2015 | -0.3 (-0.36 to -0.23) | 0 |
| Burkina Faso | 2006 - 2019 | -0.58 (-0.73 to -0.43) | 0 |
| Burkina Faso | 2010 - 1993 | -0.05 (-0.08 to -0.02) | 0.003 |
| Burundi | 1990 - 2000 | 0.22 (0.2 to 0.24) | 0 |
| Burundi | 1995 - 2006 | 0.5 (0.47 to 0.53) | 0 |
| Burundi | 2000 - 2011 | -0.29 (-0.32 to -0.26) | 0 |
| Burundi | 2005 - 2014 | 0.16 (0.13 to 0.18) | 0 |
| Burundi | 2010 - 2019 | -0.44 (-0.47 to -0.42) | 0 |
| Burundi | 2015 - 1995 | 0.1 (0.07 to 0.13) | 0 |
| Cabo Verde | 1990 - 2000 | 0.54 (0.5 to 0.58) | 0 |
| Cabo Verde | 1995 - 2005 | 0.97 (0.91 to 1.03) | 0 |
| Cabo Verde | 2000 - 2012 | 0.05 (0.04 to 0.07) | 0 |
| Cabo Verde | 2011 - 2017 | -0.16 (-0.18 to -0.14) | 0 |
| Cambodia | 1990 - 2019 | -1.05 (-1.21 to -0.88) | 0 |
| Cambodia | 1994 - 2000 | 0.69 (0.57 to 0.81) | 0 |
| Cambodia | 2000 - 2005 | -0.91 (-1.08 to -0.74) | 0 |
| Cambodia | 2005 - 2010 | -0.02 (-0.19 to 0.15) | 0.817 |
| Cambodia | 2010 - 2014 | -0.67 (-0.84 to -0.5) | 0 |
| Cambodia | 2015 - 2019 | 0.63 (0.46 to 0.8) | 0 |
| Cameroon | 1990 - 2000 | 0.3 (0.24 to 0.36) | 0 |
| Cameroon | 1995 - 2005 | 1.51 (1.43 to 1.59) | 0 |
| Cameroon | 2000 - 2011 | 0.69 (0.43 to 0.95) | 0 |
| Cameroon | 2003 - 2019 | 0.39 (0.35 to 0.44) | 0 |
| Cameroon | 2010 - 1995 | -0.28 (-0.37 to -0.2) | 0 |
| Cameroon | 2015 - 2000 | -0.05 (-0.14 to 0.03) | 0.211 |
| Canada | 1990 - 2005 | 0.02 (-0.08 to 0.13) | 0.65 |
| Canada | 1994 - 2010 | -1.69 (-1.76 to -1.63) | 0 |
| Canada | 2000 - 2017 | -0.23 (-0.32 to -0.13) | 0 |
| Canada | 2005 - 2019 | -0.54 (-0.64 to -0.45) | 0 |
| Canada | 2010 - 1995 | 0.36 (0.27 to 0.45) | 0 |
| Canada | 2015 - 2000 | -0.38 (-0.48 to -0.29) | 0 |
| Central African Republic | 1990 - 2005 | -0.38 (-0.54 to -0.22) | 0 |
| Central African Republic | 1995 - 2011 | 0.4 (0.19 to 0.62) | 0.001 |
| Central African Republic | 2000 - 2015 | -0.33 (-0.55 to -0.12) | 0.005 |
| Central African Republic | 2005 - 2019 | 0.59 (0.37 to 0.82) | 0 |
| Central African Republic | 2010 - 1993 | -0.51 (-0.86 to -0.16) | 0.008 |
| Central African Republic | 2014 - 2000 | 0.37 (0.21 to 0.53) | 0 |
| Chad | 1990 - 2006 | 0.11 (-0.16 to 0.37) | 0.412 |
| Chad | 1993 - 2011 | 0.72 (0.21 to 1.25) | 0.01 |
| Chad | 1996 - 2014 | 2.08 (1.55 to 2.61) | 0 |
| Chad | 1999 - 2019 | 0.52 (-0.02 to 1.07) | 0.058 |
| Chad | 2002 - 1995 | -0.03 (-0.08 to 0.02) | 0.256 |
| Chad | 2012 - 2000 | 0.19 (0.12 to 0.27) | 0 |
| Chile | 1990 - 2005 | -0.53 (-0.57 to -0.49) | 0 |
| Chile | 2005 - 2012 | 0.47 (0.15 to 0.78) | 0.006 |
| Chile | 2010 - 2017 | -0.6 (-1.1 to -0.1) | 0.02 |
| Chile | 2014 - 2019 | 0.53 (0.3 to 0.77) | 0 |
| China | 1990 - 2000 | 0.65 (0.63 to 0.67) | 0 |
| China | 2000 - 2005 | 0.23 (0.11 to 0.34) | 0.001 |
| China | 2004 - 2010 | -0.09 (-0.14 to -0.04) | 0.002 |
| China | 2010 - 2014 | 0.78 (0.71 to 0.85) | 0 |
| China | 2015 - 2019 | -0.3 (-0.37 to -0.23) | 0 |
| Colombia | 1990 - 2000 | -0.61 (-0.64 to -0.57) | 0 |
| Colombia | 1993 - 2005 | -0.48 (-0.49 to -0.47) | 0 |
| Colombia | 2001 - 2011 | -0.82 (-0.89 to -0.74) | 0 |
| Colombia | 2004 - 2019 | -0.17 (-0.19 to -0.16) | 0 |
| Colombia | 2011 - 1995 | -0.38 (-0.42 to -0.35) | 0 |
| Colombia | 2015 - 2000 | -0.01 (-0.03 to 0.01) | 0.369 |
| Comoros | 1990 - 2005 | 0.14 (0.11 to 0.17) | 0 |
| Comoros | 1995 - 2010 | -0.12 (-0.15 to -0.08) | 0 |
| Comoros | 2000 - 2017 | -0.45 (-0.49 to -0.42) | 0 |
| Comoros | 2005 - 2019 | 0.08 (0.05 to 0.11) | 0 |
| Comoros | 2011 - 1995 | -0.09 (-0.12 to -0.07) | 0 |
| Comoros | 2017 - 2000 | 0.1 (-0.01 to 0.22) | 0.081 |
| Congo | 1990 - 2005 | -0.27 (-0.31 to -0.23) | 0 |
| Congo | 2001 - 2011 | -0.45 (-0.71 to -0.19) | 0.002 |
| Congo | 2005 - 2015 | 0.67 (0.49 to 0.84) | 0 |
| Congo | 2010 - 2019 | -0.49 (-0.76 to -0.21) | 0.002 |
| Congo | 2014 - 1993 | 0.29 (0.17 to 0.42) | 0 |
| Cook Islands | 1990 - 2000 | 0.56 (0.54 to 0.59) | 0 |
| Cook Islands | 1993 - 2006 | 0.37 (0.36 to 0.38) | 0 |
| Cook Islands | 1999 - 2011 | 0.16 (0.15 to 0.17) | 0 |
| Cook Islands | 2005 - 2014 | -0.02 (-0.02 to -0.01) | 0 |
| Cook Islands | 2014 - 2019 | -0.1 (-0.15 to -0.05) | 0.001 |
| Cook Islands | 2017 - 1995 | -0.3 (-0.35 to -0.25) | 0 |
| Costa Rica | 1990 - 2000 | 0.04 (0.03 to 0.05) | 0 |
| Costa Rica | 1997 - 2005 | -0.01 (-0.01 to 0) | 0.017 |
| Costa Rica | 2006 - 2012 | -0.3 (-0.32 to -0.29) | 0 |
| Costa Rica | 2011 - 2017 | -0.66 (-0.72 to -0.61) | 0 |
| Costa Rica | 2014 - 2019 | -0.5 (-0.55 to -0.44) | 0 |
| Costa Rica | 2017 - 2000 | 0.04 (-0.01 to 0.1) | 0.125 |
| Coted'Ivoire | 1990 - 2005 | 0.1 (0 to 0.19) | 0.043 |
| Coted'Ivoire | 1995 - 2010 | 1.58 (1.45 to 1.72) | 0 |
| Coted'Ivoire | 2000 - 2014 | -0.41 (-0.54 to -0.29) | 0 |
| Coted'Ivoire | 2005 - 2019 | 0.46 (0.33 to 0.58) | 0 |
| Coted'Ivoire | 2010 - 2000 | -0.68 (-0.81 to -0.55) | 0 |
| Coted'Ivoire | 2015 - 2005 | 0.27 (0.13 to 0.41) | 0.001 |
| Croatia | 1990 - 2011 | -0.78 (-1.05 to -0.51) | 0 |
| Croatia | 1995 - 2019 | 0.34 (-0.03 to 0.71) | 0.069 |
| Croatia | 2000 - 1995 | -1.49 (-1.84 to -1.13) | 0 |
| Croatia | 2005 - 2000 | -0.14 (-0.51 to 0.22) | 0.414 |
| Croatia | 2010 - 2005 | -1.63 (-2.21 to -1.05) | 0 |
| Croatia | 2014 - 2010 | 0.54 (0.28 to 0.81) | 0.001 |
| Cuba | 1990 - 2017 | -0.54 (-0.57 to -0.51) | 0 |
| Cuba | 1999 - 2019 | -0.27 (-0.34 to -0.19) | 0 |
| Cuba | 2005 - 1995 | -0.52 (-0.62 to -0.42) | 0 |
| Cuba | 2010 - 2000 | 0.03 (-0.13 to 0.18) | 0.744 |
| Cuba | 2014 - 2005 | -0.2 (-0.28 to -0.13) | 0 |
| Cyprus | 1990 - 2011 | 0.24 (-0.02 to 0.51) | 0.068 |
| Cyprus | 2006 - 2015 | 7.06 (3.4 to 10.85) | 0.001 |
| Cyprus | 2010 - 2019 | -5.48 (-8.69 to -2.16) | 0.003 |
| Cyprus | 2014 - 1993 | 4.5 (2.85 to 6.17) | 0 |
| Czechia | 1990 - 2000 | -0.72 (-0.8 to -0.65) | 0 |
| Czechia | 1993 - 2006 | -0.58 (-0.61 to -0.55) | 0 |
| Czechia | 1999 - 2011 | -0.4 (-0.43 to -0.37) | 0 |
| Czechia | 2005 - 2014 | -0.92 (-0.96 to -0.87) | 0 |
| Czechia | 2010 - 2019 | -0.32 (-0.46 to -0.18) | 0 |
| Czechia | 2013 - 1995 | -0.1 (-0.13 to -0.08) | 0 |
| Democratic People's Republic of Korea | 1990 - 2000 | 0.29 (0.27 to 0.32) | 0 |
| Democratic People's Republic of Korea | 1996 - 2005 | 0.41 (0.35 to 0.48) | 0 |
| Democratic People's Republic of Korea | 2000 - 2012 | 0.69 (0.62 to 0.76) | 0 |
| Democratic People's Republic of Korea | 2004 - 2017 | 0.46 (0.43 to 0.49) | 0 |
| Democratic People's Republic of Korea | 2010 - 2019 | 0 (-0.03 to 0.03) | 0.954 |
| Democratic People's Republic of Korea | 2016 - 2000 | -0.2 (-0.26 to -0.13) | 0 |
| Democratic Republic of the Congo | 1990 - 2005 | -0.15 (-0.21 to -0.09) | 0 |
| Democratic Republic of the Congo | 1994 - 2010 | 0.06 (0.02 to 0.1) | 0.004 |
| Democratic Republic of the Congo | 2000 - 2014 | -0.53 (-0.59 to -0.48) | 0 |
| Democratic Republic of the Congo | 2005 - 2019 | 0.03 (-0.03 to 0.09) | 0.258 |
| Democratic Republic of the Congo | 2010 - 2000 | -0.36 (-0.42 to -0.31) | 0 |
| Democratic Republic of the Congo | 2015 - 2005 | 0.29 (0.23 to 0.35) | 0 |
| Denmark | 1990 - 2011 | -0.64 (-0.66 to -0.62) | 0 |
| Denmark | 2000 - 2019 | -0.19 (-0.45 to 0.07) | 0.135 |
| Denmark | 2003 - 1995 | -0.04 (-0.08 to 0.01) | 0.085 |
| Denmark | 2010 - 2000 | -0.8 (-1.06 to -0.54) | 0 |
| Denmark | 2013 - 2005 | -1.25 (-1.38 to -1.12) | 0 |
| Denmark | 2017 - 2010 | 0.04 (-0.26 to 0.34) | 0.779 |
| Djibouti | 1990 - 2017 | 0.55 (0.53 to 0.57) | 0 |
| Djibouti | 2000 - 2019 | 0.24 (0.22 to 0.26) | 0 |
| Djibouti | 2010 - 1995 | -0.16 (-0.19 to -0.12) | 0 |
| Djibouti | 2017 - 2000 | 0.05 (-0.17 to 0.26) | 0.658 |
| Dominica | 1990 - 2005 | -0.11 (-0.12 to -0.11) | 0 |
| Dominica | 2000 - 2011 | 0.11 (0.08 to 0.14) | 0 |
| Dominica | 2005 - 2015 | 0.28 (0.25 to 0.31) | 0 |
| Dominica | 2010 - 2019 | -0.28 (-0.31 to -0.25) | 0 |
| Dominica | 2015 - 1993 | 0.13 (0.09 to 0.16) | 0 |
| Dominican Republic | 1990 - 2000 | 0.66 (0.61 to 0.72) | 0 |
| Dominican Republic | 1994 - 2006 | 0.3 (0.29 to 0.32) | 0 |
| Dominican Republic | 2004 - 2011 | 0.52 (0.48 to 0.56) | 0 |
| Dominican Republic | 2010 - 2014 | -0.21 (-0.27 to -0.16) | 0 |
| Dominican Republic | 2015 - 2019 | 0.08 (0.02 to 0.13) | 0.009 |
| Ecuador | 1990 - 1995 | 0.23 (0.06 to 0.4) | 0.011 |
| Ecuador | 1995 - 2000 | -0.68 (-0.91 to -0.46) | 0 |
| Ecuador | 2000 - 2005 | 0.18 (-0.04 to 0.39) | 0.102 |
| Ecuador | 2005 - 2012 | 1.57 (1.34 to 1.8) | 0 |
| Ecuador | 2010 - 2017 | -0.82 (-1.18 to -0.45) | 0 |
| Ecuador | 2014 - 2019 | 0.43 (0.26 to 0.6) | 0 |
| Egypt | 1990 - 2000 | -0.95 (-1.21 to -0.69) | 0 |
| Egypt | 1994 - 2005 | 0.24 (0.06 to 0.43) | 0.014 |
| Egypt | 2000 - 2010 | -0.4 (-0.66 to -0.15) | 0.005 |
| Egypt | 2005 - 2014 | 1.29 (1.03 to 1.55) | 0 |
| Egypt | 2010 - 2019 | -0.84 (-1.25 to -0.43) | 0.001 |
| Egypt | 2014 - 2000 | 0.47 (0.28 to 0.65) | 0 |
| El Salvador | 1990 - 2005 | 0.3 (0.26 to 0.34) | 0 |
| El Salvador | 1997 - 2011 | 0.51 (0.38 to 0.65) | 0 |
| El Salvador | 2001 - 2019 | 0.84 (0.57 to 1.11) | 0 |
| El Salvador | 2004 - 1995 | 0.41 (0.35 to 0.47) | 0 |
| El Salvador | 2010 - 2000 | -0.35 (-0.48 to -0.21) | 0 |
| El Salvador | 2014 - 2005 | 0.15 (0.09 to 0.21) | 0 |
| Equatorial Guinea | 1990 - 2010 | -0.5 (-0.59 to -0.42) | 0 |
| Equatorial Guinea | 1993 - 2017 | -0.18 (-0.2 to -0.16) | 0 |
| Equatorial Guinea | 2001 - 2019 | -0.55 (-0.57 to -0.53) | 0 |
| Equatorial Guinea | 2010 - 1995 | -0.17 (-0.25 to -0.08) | 0.001 |
| Equatorial Guinea | 2014 - 2000 | 0.06 (-0.11 to 0.22) | 0.463 |
| Equatorial Guinea | 2017 - 2005 | -0.13 (-0.32 to 0.05) | 0.141 |
| Eritrea | 1990 - 2011 | 0.1 (0.08 to 0.12) | 0 |
| Eritrea | 1995 - 2015 | -0.05 (-0.05 to -0.04) | 0 |
| Eritrea | 2008 - 2019 | -0.16 (-0.18 to -0.14) | 0 |
| Eritrea | 2014 - 1993 | -0.5 (-0.59 to -0.41) | 0 |
| Eritrea | 2017 - 2000 | 0.04 (-0.05 to 0.14) | 0.34 |
| Estonia | 1990 - 2006 | 0.46 (0.31 to 0.61) | 0 |
| Estonia | 1995 - 2011 | -0.73 (-0.93 to -0.52) | 0 |
| Estonia | 2000 - 2014 | 0.42 (0.21 to 0.62) | 0.001 |
| Estonia | 2005 - 2019 | 0.05 (-0.02 to 0.12) | 0.118 |
| Estonia | 2014 - 1995 | -0.47 (-0.62 to -0.32) | 0 |
| Eswatini | 1990 - 2000 | 0.37 (0.35 to 0.4) | 0 |
| Eswatini | 1996 - 2005 | 1.17 (1.03 to 1.3) | 0 |
| Eswatini | 1999 - 2012 | 0.46 (0.43 to 0.49) | 0 |
| Eswatini | 2005 - 2017 | -0.02 (-0.06 to 0.02) | 0.341 |
| Eswatini | 2010 - 2019 | -0.55 (-0.57 to -0.53) | 0 |
| Eswatini | 2017 - 2000 | -0.15 (-0.29 to -0.01) | 0.035 |
| Ethiopia | 1990 - 2005 | 0.25 (0.23 to 0.26) | 0 |
| Ethiopia | 2000 - 2010 | 0.16 (0.14 to 0.17) | 0 |
| Ethiopia | 2010 - 2014 | -0.07 (-0.16 to 0.02) | 0.097 |
| Ethiopia | 2014 - 2019 | -0.44 (-0.62 to -0.27) | 0 |
| Ethiopia | 2017 - 2000 | 0.13 (-0.05 to 0.32) | 0.145 |
| Fiji | 1990 - 2005 | -0.35 (-0.46 to -0.24) | 0 |
| Fiji | 1994 - 2011 | 0.17 (0.1 to 0.25) | 0 |
| Fiji | 2000 - 2019 | 0.03 (-0.08 to 0.14) | 0.599 |
| Fiji | 2005 - 1995 | 0.59 (0.48 to 0.7) | 0 |
| Fiji | 2010 - 2000 | -0.26 (-0.43 to -0.08) | 0.007 |
| Fiji | 2014 - 2005 | -0.03 (-0.11 to 0.04) | 0.373 |
| Finland | 1990 - 2010 | -1.99 (-2.14 to -1.84) | 0 |
| Finland | 1994 - 2017 | -1.44 (-1.55 to -1.34) | 0 |
| Finland | 2000 - 2019 | -0.01 (-0.04 to 0.02) | 0.426 |
| Finland | 2012 - 1995 | 0.28 (0.22 to 0.34) | 0 |
| France | 1990 - 2000 | -0.69 (-0.93 to -0.45) | 0 |
| France | 1995 - 2005 | -0.05 (-0.15 to 0.04) | 0.221 |
| France | 2005 - 2011 | 1.56 (1.24 to 1.89) | 0 |
| France | 2010 - 2015 | -1.26 (-1.76 to -0.75) | 0 |
| France | 2014 - 2019 | 0.65 (0.41 to 0.88) | 0 |
| Gabon | 1990 - 1993 | 0.03 (-0.01 to 0.06) | 0.097 |
| Gabon | 1996 - 2000 | 0.14 (0.08 to 0.21) | 0 |
| Gabon | 2001 - 2006 | 0.41 (0.22 to 0.6) | 0.001 |
| Gabon | 2004 - 2011 | 0.07 (-0.02 to 0.17) | 0.113 |
| Gabon | 2008 - 2014 | -0.03 (-0.07 to 0.02) | 0.186 |
| Gabon | 2014 - 2019 | -0.16 (-0.21 to -0.12) | 0 |
| Gambia | 1990 - 1995 | -0.05 (-0.19 to 0.09) | 0.449 |
| Gambia | 1995 - 2000 | 1.28 (1.08 to 1.49) | 0 |
| Gambia | 2000 - 2005 | -0.03 (-0.23 to 0.17) | 0.737 |
| Gambia | 2005 - 2012 | 0.86 (0.66 to 1.06) | 0 |
| Gambia | 2010 - 2017 | -0.56 (-0.88 to -0.24) | 0.002 |
| Gambia | 2014 - 2019 | 0.26 (0.11 to 0.4) | 0.002 |
| Georgia | 1990 - 2000 | -0.26 (-0.4 to -0.12) | 0.002 |
| Georgia | 1995 - 2005 | 0.34 (0.14 to 0.54) | 0.003 |
| Georgia | 2000 - 2010 | -0.03 (-0.23 to 0.17) | 0.732 |
| Georgia | 2005 - 2014 | 0.64 (0.44 to 0.85) | 0 |
| Georgia | 2010 - 2019 | -0.29 (-0.61 to 0.03) | 0.074 |
| Georgia | 2014 - 2000 | 0.31 (0.16 to 0.46) | 0.001 |
| Germany | 1990 - 2005 | -0.48 (-0.69 to -0.27) | 0 |
| Germany | 1995 - 2011 | -1.12 (-1.21 to -1.03) | 0 |
| Germany | 2004 - 2019 | 0.12 (0.07 to 0.18) | 0 |
| Germany | 2015 - 1995 | 0.85 (0.59 to 1.11) | 0 |
| Ghana | 1990 - 2000 | -0.17 (-0.27 to -0.07) | 0.002 |
| Ghana | 1995 - 2005 | 1.37 (1.24 to 1.5) | 0 |
| Ghana | 2000 - 2010 | -0.69 (-0.81 to -0.57) | 0 |
| Ghana | 2005 - 2017 | 0.98 (0.86 to 1.1) | 0 |
| Ghana | 2010 - 2019 | -0.18 (-0.31 to -0.05) | 0.012 |
| Ghana | 2015 - 1995 | 0.19 (0.05 to 0.33) | 0.013 |
| Greece | 1990 - 2000 | -0.28 (-0.35 to -0.2) | 0 |
| Greece | 1995 - 2005 | 0.1 (0.07 to 0.12) | 0 |
| Greece | 2005 - 2011 | 1.41 (1.31 to 1.51) | 0 |
| Greece | 2010 - 2015 | -0.22 (-0.37 to -0.07) | 0.008 |
| Greece | 2014 - 2019 | 1.62 (1.33 to 1.92) | 0 |
| Greece | 2017 - 1993 | 0.11 (-0.21 to 0.44) | 0.462 |
| Greenland | 1990 - 2000 | 0.24 (-0.04 to 0.51) | 0.084 |
| Greenland | 1992 - 2006 | -0.19 (-0.42 to 0.04) | 0.095 |
| Greenland | 1995 - 2011 | -0.78 (-0.85 to -0.7) | 0 |
| Greenland | 2000 - 2014 | -0.34 (-0.42 to -0.26) | 0 |
| Greenland | 2005 - 2019 | -0.49 (-0.54 to -0.43) | 0 |
| Greenland | 2011 - 1995 | -0.08 (-0.1 to -0.05) | 0 |
| Grenada | 1990 - 2000 | -0.11 (-0.2 to -0.03) | 0.011 |
| Grenada | 1995 - 2005 | 0.25 (0.06 to 0.43) | 0.012 |
| Grenada | 1999 - 2012 | 0.03 (0.01 to 0.04) | 0.001 |
| Grenada | 2015 - 2017 | 0.4 (0.27 to 0.52) | 0 |
| Guam | 1990 - 2019 | 1.07 (1 to 1.14) | 0 |
| Guam | 1995 - 2000 | 1.65 (1.49 to 1.82) | 0 |
| Guam | 1999 - 2005 | 0.53 (0.47 to 0.58) | 0 |
| Guam | 2006 - 2010 | 0.74 (0.42 to 1.07) | 0 |
| Guam | 2009 - 2014 | 0.21 (0.11 to 0.31) | 0.001 |
| Guam | 2014 - 2019 | -0.26 (-0.33 to -0.18) | 0 |
| Guatemala | 1990 - 2000 | 0.01 (-0.05 to 0.07) | 0.725 |
| Guatemala | 1995 - 2005 | 0.51 (0.43 to 0.59) | 0 |
| Guatemala | 2000 - 2011 | 0.85 (0.82 to 0.88) | 0 |
| Guatemala | 2009 - 2019 | 0.33 (0.29 to 0.36) | 0 |
| Guatemala | 2017 - 1995 | 0.11 (-0.15 to 0.37) | 0.386 |
| Guinea | 1990 - 2000 | 0.16 (0.07 to 0.25) | 0.001 |
| Guinea | 1995 - 2005 | 0.53 (0.5 to 0.57) | 0 |
| Guinea | 2005 - 2010 | 1.45 (1.25 to 1.65) | 0 |
| Guinea | 2009 - 2017 | 0.34 (0.3 to 0.37) | 0 |
| Guinea-Bissau | 1990 - 2019 | -0.19 (-0.36 to -0.01) | 0.036 |
| Guinea-Bissau | 1995 - 1995 | 1.33 (1.07 to 1.58) | 0 |
| Guinea-Bissau | 2000 - 2000 | -0.2 (-0.45 to 0.06) | 0.12 |
| Guinea-Bissau | 2005 - 2005 | 0.87 (0.62 to 1.13) | 0 |
| Guinea-Bissau | 2010 - 2011 | -0.69 (-1.09 to -0.3) | 0.002 |
| Guinea-Bissau | 2014 - 2015 | 0.27 (0.09 to 0.45) | 0.007 |
| Guyana | 1990 - 2019 | -0.41 (-0.51 to -0.31) | 0 |
| Guyana | 1995 - 1993 | 0.73 (0.59 to 0.87) | 0 |
| Guyana | 2000 - 2000 | -0.62 (-0.75 to -0.48) | 0 |
| Guyana | 2005 - 2006 | 0.28 (0.14 to 0.41) | 0.001 |
| Guyana | 2010 - 2011 | -0.75 (-0.88 to -0.62) | 0 |
| Guyana | 2015 - 2014 | 0.47 (0.33 to 0.62) | 0 |
| Haiti | 1990 - 2019 | -0.4 (-0.6 to -0.21) | 0.001 |
| Haiti | 1995 - 1995 | 0.79 (0.51 to 1.07) | 0 |
| Haiti | 2000 - 2000 | -0.77 (-1.05 to -0.5) | 0 |
| Haiti | 2005 - 2005 | 0.49 (0.21 to 0.77) | 0.002 |
| Haiti | 2010 - 2012 | -0.95 (-1.39 to -0.5) | 0 |
| Haiti | 2014 - 2017 | 0.55 (0.35 to 0.76) | 0 |
| Honduras | 1990 - 2019 | 0.19 (0.09 to 0.3) | 0.002 |
| Honduras | 1996 - 2000 | 0.66 (0.04 to 1.28) | 0.039 |
| Honduras | 1999 - 2005 | 0.12 (0.01 to 0.23) | 0.033 |
| Honduras | 2006 - 2010 | 0.4 (0.09 to 0.72) | 0.017 |
| Honduras | 2010 - 2014 | -0.74 (-1.05 to -0.42) | 0 |
| Honduras | 2014 - 2019 | 0.4 (0.25 to 0.54) | 0 |
| Hungary | 1990 - 2000 | -0.63 (-0.67 to -0.59) | 0 |
| Hungary | 2000 - 2005 | -1.56 (-1.71 to -1.4) | 0 |
| Hungary | 2005 - 2011 | -0.02 (-0.18 to 0.14) | 0.828 |
| Hungary | 2010 - 2019 | -1.58 (-1.74 to -1.42) | 0 |
| Hungary | 2015 - 1995 | 0.54 (0.37 to 0.7) | 0 |
| Iceland | 1990 - 2000 | 0.4 (0.32 to 0.48) | 0 |
| Iceland | 1994 - 2005 | -0.54 (-0.58 to -0.5) | 0 |
| Iceland | 2001 - 2010 | 0.19 (-0.03 to 0.42) | 0.082 |
| Iceland | 2004 - 2017 | -0.22 (-0.27 to -0.17) | 0 |
| Iceland | 2010 - 2019 | 0.13 (0.1 to 0.15) | 0 |
| India | 1990 - 1995 | 0.14 (0.13 to 0.16) | 0 |
| India | 2000 - 2000 | -0.77 (-0.82 to -0.73) | 0 |
| India | 2005 - 2005 | 0.31 (0.28 to 0.34) | 0 |
| India | 2011 - 2011 | 1.58 (1.43 to 1.73) | 0 |
| India | 2014 - 2015 | 0.08 (0.05 to 0.12) | 0 |
| Indonesia | 1990 - 2019 | 0.15 (-0.02 to 0.33) | 0.083 |
| Indonesia | 1995 - 1993 | 0.35 (0.28 to 0.42) | 0 |
| Indonesia | 2005 - 2000 | 0.98 (0.73 to 1.23) | 0 |
| Indonesia | 2010 - 2006 | -0.56 (-0.95 to -0.17) | 0.008 |
| Indonesia | 2014 - 2011 | 0.41 (0.24 to 0.59) | 0 |
| Iran (Islamic Republic of) | 1990 - 2014 | -0.27 (-0.34 to -0.2) | 0 |
| Iran (Islamic Republic of) | 1993 - 2019 | -0.08 (-0.09 to -0.08) | 0 |
| Iran (Islamic Republic of) | 2011 - 1995 | -0.52 (-0.66 to -0.39) | 0 |
| Iran (Islamic Republic of) | 2014 - 2000 | -0.26 (-0.4 to -0.13) | 0.001 |
| Iran (Islamic Republic of) | 2017 - 2005 | -0.01 (-0.15 to 0.14) | 0.932 |
| Iraq | 1990 - 2012 | -0.77 (-1.02 to -0.52) | 0 |
| Iraq | 1994 - 2017 | 0.36 (0.19 to 0.54) | 0.001 |
| Iraq | 2000 - 2019 | -0.83 (-1.08 to -0.59) | 0 |
| Iraq | 2005 - 2000 | 0.62 (0.37 to 0.87) | 0 |
| Iraq | 2010 - 2005 | -0.86 (-1.25 to -0.46) | 0 |
| Iraq | 2014 - 2010 | 0.56 (0.37 to 0.74) | 0 |
| Ireland | 1990 - 2014 | -0.34 (-0.51 to -0.16) | 0.001 |
| Ireland | 1995 - 2019 | 0.29 (0.05 to 0.53) | 0.024 |
| Ireland | 2000 - 2000 | -1.13 (-1.37 to -0.9) | 0 |
| Ireland | 2005 - 2005 | 0.49 (0.25 to 0.73) | 0.001 |
| Ireland | 2010 - 2011 | -1.4 (-1.63 to -1.18) | 0 |
| Ireland | 2015 - 2019 | 0.91 (0.67 to 1.15) | 0 |
| Israel | 1990 - 1995 | -0.18 (-0.21 to -0.15) | 0 |
| Israel | 2005 - 2000 | 0.51 (0.27 to 0.74) | 0 |
| Israel | 2010 - 2005 | -0.26 (-0.62 to 0.11) | 0.159 |
| Israel | 2014 - 2010 | 0.54 (0.37 to 0.71) | 0 |
| Italy | 1990 - 2017 | -1.72 (-1.82 to -1.62) | 0 |
| Italy | 1994 - 2019 | -0.73 (-0.75 to -0.7) | 0 |
| Italy | 2005 - 1995 | 0.28 (0.21 to 0.35) | 0 |
| Italy | 2011 - 2000 | -0.15 (-0.46 to 0.16) | 0.316 |
| Italy | 2014 - 2005 | 1.13 (0.82 to 1.44) | 0 |
| Italy | 2017 - 2011 | 0.17 (-0.16 to 0.51) | 0.287 |
| Jamaica | 1990 - 2015 | 0.2 (0.15 to 0.25) | 0 |
| Jamaica | 1996 - 2019 | 0.61 (0.6 to 0.63) | 0 |
| Jamaica | 2009 - 1993 | 0.15 (0.08 to 0.21) | 0 |
| Jamaica | 2015 - 2000 | 0.36 (0.26 to 0.45) | 0 |
| Japan | 1990 - 2006 | -0.85 (-0.93 to -0.78) | 0 |
| Japan | 1995 - 2011 | -0.38 (-0.41 to -0.35) | 0 |
| Japan | 2005 - 2014 | 0.86 (0.75 to 0.96) | 0 |
| Japan | 2010 - 2019 | -0.8 (-0.96 to -0.63) | 0 |
| Japan | 2014 - 1995 | 0.01 (-0.06 to 0.09) | 0.681 |
| Jordan | 1990 - 2000 | -0.24 (-0.3 to -0.17) | 0 |
| Jordan | 1995 - 2005 | 0.47 (0.38 to 0.57) | 0 |
| Jordan | 2000 - 2012 | -0.82 (-0.91 to -0.73) | 0 |
| Jordan | 2005 - 2017 | 0.01 (-0.08 to 0.1) | 0.851 |
| Jordan | 2010 - 2019 | -0.67 (-0.76 to -0.58) | 0 |
| Jordan | 2015 - 2000 | 0.32 (0.22 to 0.41) | 0 |
| Kazakhstan | 1990 - 2005 | -0.21 (-0.29 to -0.14) | 0 |
| Kazakhstan | 1994 - 2010 | 0.02 (-0.09 to 0.13) | 0.645 |
| Kazakhstan | 1998 - 2014 | -0.18 (-0.42 to 0.05) | 0.117 |
| Kazakhstan | 2001 - 2019 | -0.86 (-0.89 to -0.83) | 0 |
| Kazakhstan | 2009 - 2000 | -0.75 (-0.82 to -0.68) | 0 |
| Kazakhstan | 2014 - 2005 | 0.03 (-0.02 to 0.08) | 0.199 |
| Kenya | 1990 - 2011 | 0.26 (0.21 to 0.31) | 0 |
| Kenya | 1995 - 2019 | 1.39 (1.32 to 1.46) | 0 |
| Kenya | 2000 - 1995 | -0.1 (-0.15 to -0.05) | 0.001 |
| Kenya | 2006 - 2000 | -1.58 (-1.8 to -1.35) | 0 |
| Kenya | 2009 - 2005 | -0.31 (-0.34 to -0.28) | 0 |
| Kenya | 2017 - 2010 | 0.03 (-0.2 to 0.25) | 0.807 |
| Kiribati | 1990 - 2017 | 0.16 (0.05 to 0.27) | 0.009 |
| Kiribati | 1995 - 2019 | 0.59 (0.34 to 0.84) | 0 |
| Kiribati | 1999 - 1995 | 0.29 (0.26 to 0.33) | 0 |
| Kiribati | 2010 - 2000 | -0.32 (-0.57 to -0.08) | 0.014 |
| Kiribati | 2014 - 2005 | 0.06 (-0.05 to 0.17) | 0.284 |
| Kuwait | 1990 - 2011 | -0.01 (-0.12 to 0.11) | 0.925 |
| Kuwait | 1995 - 2015 | 0.82 (0.65 to 0.98) | 0 |
| Kuwait | 2000 - 2019 | -0.22 (-0.38 to -0.06) | 0.013 |
| Kuwait | 2005 - 1993 | 0.26 (0.1 to 0.42) | 0.004 |
| Kuwait | 2010 - 2000 | -0.79 (-1.04 to -0.54) | 0 |
| Kuwait | 2014 - 2006 | 0.23 (0.11 to 0.35) | 0.001 |
| Kyrgyzstan | 1990 - 2011 | -0.3 (-0.32 to -0.28) | 0 |
| Kyrgyzstan | 1996 - 2014 | -0.45 (-0.49 to -0.41) | 0 |
| Kyrgyzstan | 2001 - 2019 | -0.59 (-0.71 to -0.47) | 0 |
| Kyrgyzstan | 2004 - 1995 | -0.33 (-0.35 to -0.3) | 0 |
| Kyrgyzstan | 2011 - 2000 | -0.51 (-0.63 to -0.39) | 0 |
| Kyrgyzstan | 2014 - 2005 | -0.04 (-0.07 to -0.01) | 0.012 |
| Lao People's Democratic Republic | 1990 - 2012 | -1.5 (-1.98 to -1.02) | 0 |
| Lao People's Democratic Republic | 1993 - 2017 | -0.34 (-0.5 to -0.19) | 0 |
| Lao People's Democratic Republic | 2000 - 2019 | -1.13 (-1.4 to -0.86) | 0 |
| Lao People's Democratic Republic | 2005 - 2000 | 0.96 (0.67 to 1.25) | 0 |
| Lao People's Democratic Republic | 2010 - 2005 | -0.89 (-1.37 to -0.41) | 0.002 |
| Lao People's Democratic Republic | 2014 - 2010 | 0.54 (0.33 to 0.75) | 0 |
| Latvia | 1990 - 2014 | 0.27 (0.23 to 0.31) | 0 |
| Latvia | 2006 - 2019 | 0.76 (0.2 to 1.33) | 0.01 |
| Latvia | 2010 - 2000 | -1.11 (-1.66 to -0.56) | 0 |
| Latvia | 2014 - 2005 | 0.64 (0.39 to 0.89) | 0 |
| Lebanon | 1990 - 2011 | -0.76 (-1.01 to -0.51) | 0 |
| Lebanon | 1994 - 2019 | -0.04 (-0.22 to 0.13) | 0.594 |
| Lebanon | 2000 - 1995 | 0.45 (0.2 to 0.69) | 0.002 |
| Lebanon | 2005 - 2000 | 0.86 (0.62 to 1.11) | 0 |
| Lebanon | 2010 - 2005 | -0.03 (-0.42 to 0.36) | 0.867 |
| Lebanon | 2014 - 2010 | 0.57 (0.39 to 0.75) | 0 |
| Lesotho | 1990 - 2017 | 0.95 (0.92 to 0.98) | 0 |
| Lesotho | 1998 - 2019 | 0.7 (0.64 to 0.77) | 0 |
| Lesotho | 2004 - 1995 | 0.31 (0.23 to 0.4) | 0 |
| Lesotho | 2009 - 2000 | -0.13 (-0.22 to -0.03) | 0.011 |
| Lesotho | 2014 - 2005 | -0.57 (-0.84 to -0.29) | 0.001 |
| Lesotho | 2017 - 2011 | 0.06 (-0.24 to 0.37) | 0.658 |
| Liberia | 1990 - 2015 | -0.44 (-0.65 to -0.23) | 0 |
| Liberia | 1995 - 2019 | 0.88 (0.59 to 1.18) | 0 |
| Liberia | 2000 - 1993 | -0.1 (-0.39 to 0.2) | 0.481 |
| Liberia | 2005 - 2000 | 1.17 (0.87 to 1.47) | 0 |
| Liberia | 2010 - 2006 | -0.4 (-0.88 to 0.07) | 0.089 |
| Liberia | 2014 - 2011 | 0.5 (0.29 to 0.72) | 0 |
| Libya | 1990 - 2014 | 0.53 (0.43 to 0.63) | 0 |
| Libya | 1995 - 2019 | 0.95 (0.81 to 1.08) | 0 |
| Libya | 2000 - 1995 | 0.11 (-0.1 to 0.33) | 0.282 |
| Libya | 2004 - 2000 | -0.13 (-0.16 to -0.09) | 0 |
| Libya | 2014 - 2005 | 1.33 (0.91 to 1.75) | 0 |
| Libya | 2017 - 2012 | 0.16 (-0.31 to 0.63) | 0.471 |
| Lithuania | 1990 - 2017 | -1.19 (-1.73 to -0.65) | 0 |
| Lithuania | 1993 - 2019 | -0.26 (-0.33 to -0.19) | 0 |
| Lithuania | 2005 - 2000 | 0.29 (-0.05 to 0.63) | 0.092 |
| Lithuania | 2010 - 2005 | -1.02 (-1.55 to -0.49) | 0.001 |
| Lithuania | 2014 - 2010 | 0.63 (0.39 to 0.88) | 0 |
| Luxembourg | 1990 - 2014 | -1.03 (-1.66 to -0.4) | 0.003 |
| Luxembourg | 1994 - 2019 | 0.26 (0.13 to 0.38) | 0.001 |
| Luxembourg | 2006 - 2000 | 2.66 (1.63 to 3.7) | 0 |
| Luxembourg | 2010 - 2005 | -3.06 (-4.02 to -2.08) | 0 |
| Luxembourg | 2014 - 2011 | 1.21 (0.77 to 1.65) | 0 |
| Madagascar | 1990 - 2019 | 0.52 (0.52 to 0.53) | 0 |
| Madagascar | 2004 - 1995 | 0.35 (0.32 to 0.38) | 0 |
| Madagascar | 2010 - 2000 | -0.14 (-0.17 to -0.12) | 0 |
| Madagascar | 2017 - 2005 | 0.01 (-0.13 to 0.15) | 0.872 |
| Malawi | 1990 - 2010 | 0.09 (-0.11 to 0.29) | 0.378 |
| Malawi | 1994 - 2017 | 1.14 (1 to 1.28) | 0 |
| Malawi | 2000 - 2019 | -0.42 (-0.45 to -0.39) | 0 |
| Malawi | 2015 - 1995 | 0.27 (0.07 to 0.47) | 0.009 |
| Malaysia | 1990 - 2000 | -1.41 (-1.62 to -1.2) | 0 |
| Malaysia | 1994 - 2005 | 0.13 (0.02 to 0.24) | 0.025 |
| Malaysia | 2001 - 2011 | -0.31 (-0.62 to -0.01) | 0.043 |
| Malaysia | 2005 - 2015 | 1.87 (1.67 to 2.07) | 0 |
| Malaysia | 2010 - 2019 | -0.46 (-0.79 to -0.13) | 0.01 |
| Malaysia | 2014 - 1993 | 0.35 (0.2 to 0.5) | 0 |
| Maldives | 1990 - 2000 | -0.37 (-0.51 to -0.23) | 0 |
| Maldives | 1994 - 2006 | -0.07 (-0.16 to 0.03) | 0.164 |
| Maldives | 2000 - 2011 | -0.98 (-1.11 to -0.85) | 0 |
| Maldives | 2005 - 2014 | -0.36 (-0.57 to -0.16) | 0.002 |
| Maldives | 2009 - 2019 | -0.14 (-0.27 to 0) | 0.047 |
| Maldives | 2014 - 1995 | 0.27 (0.17 to 0.36) | 0 |
| Mali | 1990 - 2000 | -0.46 (-0.61 to -0.3) | 0 |
| Mali | 1994 - 2005 | 0.31 (0.19 to 0.42) | 0 |
| Mali | 2000 - 2012 | -0.16 (-0.32 to 0) | 0.044 |
| Mali | 2005 - 2017 | 0.69 (0.53 to 0.85) | 0 |
| Mali | 2010 - 2019 | -0.03 (-0.28 to 0.22) | 0.798 |
| Mali | 2014 - 2000 | 0.29 (0.18 to 0.41) | 0 |
| Malta | 1990 - 2005 | -1.21 (-1.69 to -0.72) | 0 |
| Malta | 1995 - 2010 | 0.09 (-0.6 to 0.78) | 0.789 |
| Malta | 2000 - 2014 | -1.17 (-1.83 to -0.5) | 0.002 |
| Malta | 2005 - 2019 | 2.35 (1.64 to 3.07) | 0 |
| Malta | 2010 - 2000 | -2.25 (-3.33 to -1.15) | 0.001 |
| Malta | 2014 - 2005 | 1.23 (0.74 to 1.71) | 0 |
| Marshall Islands | 1990 - 2011 | 0.7 (0.64 to 0.77) | 0 |
| Marshall Islands | 1993 - 2019 | 0.29 (0.27 to 0.31) | 0 |
| Marshall Islands | 2000 - 1995 | 0.02 (-0.03 to 0.06) | 0.378 |
| Marshall Islands | 2005 - 2000 | 0.23 (0.18 to 0.27) | 0 |
| Marshall Islands | 2010 - 2005 | -0.23 (-0.27 to -0.18) | 0 |
| Marshall Islands | 2015 - 2010 | 0.03 (-0.01 to 0.07) | 0.143 |
| Mauritania | 1990 - 2017 | 0.15 (0.04 to 0.25) | 0.009 |
| Mauritania | 1995 - 2019 | 0.71 (0.57 to 0.86) | 0 |
| Mauritania | 2000 - 1995 | -0.51 (-0.65 to -0.37) | 0 |
| Mauritania | 2005 - 2000 | 0.65 (0.5 to 0.79) | 0 |
| Mauritania | 2010 - 2005 | -0.48 (-0.71 to -0.25) | 0.001 |
| Mauritania | 2014 - 2011 | 0.13 (0.03 to 0.24) | 0.02 |
| Mauritius | 1990 - 2015 | -0.29 (-0.32 to -0.25) | 0 |
| Mauritius | 2000 - 2019 | -1.45 (-1.58 to -1.32) | 0 |
| Mauritius | 2005 - 1993 | 0.91 (0.77 to 1.04) | 0 |
| Mauritius | 2010 - 2000 | -1.01 (-1.15 to -0.87) | 0 |
| Mauritius | 2015 - 2006 | 0.48 (0.33 to 0.62) | 0 |
| Mexico | 1990 - 2011 | -0.52 (-0.6 to -0.44) | 0 |
| Mexico | 1996 - 2014 | -1.39 (-1.85 to -0.92) | 0 |
| Mexico | 1999 - 2019 | -0.66 (-0.77 to -0.56) | 0 |
| Mexico | 2005 - 1995 | 0.19 (0.04 to 0.34) | 0.019 |
| Mexico | 2010 - 2000 | -0.79 (-0.88 to -0.71) | 0 |
| Mexico | 2017 - 2005 | -0.26 (-0.75 to 0.23) | 0.265 |
| Micronesia (Federated States of) | 1990 - 2012 | -0.01 (-0.07 to 0.04) | 0.567 |
| Micronesia (Federated States of) | 1995 - 2017 | -0.41 (-0.49 to -0.33) | 0 |
| Micronesia (Federated States of) | 2000 - 2019 | -0.15 (-0.23 to -0.08) | 0.001 |
| Micronesia (Federated States of) | 2005 - 2000 | 0.08 (0 to 0.15) | 0.053 |
| Micronesia (Federated States of) | 2010 - 2005 | -0.45 (-0.57 to -0.33) | 0 |
| Micronesia (Federated States of) | 2014 - 2010 | -0.23 (-0.28 to -0.17) | 0 |
| Monaco | 1990 - 2014 | -0.35 (-0.37 to -0.32) | 0 |
| Monaco | 2005 - 2019 | 0.49 (0.33 to 0.66) | 0 |
| Monaco | 2010 - 2000 | -0.32 (-0.48 to -0.16) | 0.001 |
| Monaco | 2015 - 2005 | 0.67 (0.5 to 0.84) | 0 |
| Mongolia | 1990 - 2011 | -0.51 (-0.68 to -0.35) | 0 |
| Mongolia | 1995 - 2019 | 0.64 (0.4 to 0.87) | 0 |
| Mongolia | 2000 - 1995 | -0.2 (-0.43 to 0.03) | 0.082 |
| Mongolia | 2005 - 2000 | 0.5 (0.27 to 0.74) | 0 |
| Mongolia | 2010 - 2005 | -0.81 (-1.17 to -0.46) | 0 |
| Mongolia | 2014 - 2010 | 0.36 (0.19 to 0.52) | 0 |
| Montenegro | 1990 - 2017 | -0.12 (-0.19 to -0.06) | 0.001 |
| Montenegro | 1995 - 2019 | 0.23 (0.14 to 0.32) | 0 |
| Montenegro | 2000 - 1995 | -0.67 (-0.76 to -0.58) | 0 |
| Montenegro | 2005 - 2000 | 0.07 (-0.02 to 0.16) | 0.131 |
| Montenegro | 2010 - 2005 | -1.45 (-1.53 to -1.36) | 0 |
| Montenegro | 2015 - 2011 | 0.45 (0.36 to 0.53) | 0 |
| Morocco | 1990 - 2015 | -0.01 (-0.11 to 0.1) | 0.889 |
| Morocco | 1995 - 2019 | 1.03 (0.89 to 1.18) | 0 |
| Morocco | 2000 - 1993 | -0.86 (-1.01 to -0.72) | 0 |
| Morocco | 2005 - 2000 | -0.23 (-0.33 to -0.13) | 0 |
| Morocco | 2011 - 2006 | -1.1 (-1.53 to -0.67) | 0 |
| Morocco | 2014 - 2011 | 0.16 (0.06 to 0.27) | 0.005 |
| Mozambique | 1990 - 2014 | -0.28 (-0.42 to -0.14) | 0.001 |
| Mozambique | 1995 - 2019 | 0.64 (0.45 to 0.84) | 0 |
| Mozambique | 2000 - 1995 | -0.01 (-0.21 to 0.18) | 0.889 |
| Mozambique | 2005 - 2000 | 0.6 (0.41 to 0.79) | 0 |
| Mozambique | 2010 - 2005 | -0.39 (-0.69 to -0.08) | 0.017 |
| Mozambique | 2014 - 2012 | 0.33 (0.19 to 0.47) | 0 |
| Myanmar | 1990 - 2017 | -1.87 (-2.33 to -1.41) | 0 |
| Myanmar | 1993 - 2019 | -0.68 (-0.83 to -0.52) | 0 |
| Myanmar | 2000 - 2000 | -1.39 (-1.67 to -1.11) | 0 |
| Myanmar | 2005 - 2005 | 1.82 (1.52 to 2.11) | 0 |
| Myanmar | 2010 - 2010 | -1.08 (-1.38 to -0.79) | 0 |
| Myanmar | 2015 - 2014 | 0.99 (0.69 to 1.3) | 0 |
| Namibia | 1990 - 2019 | 0.41 (0.3 to 0.51) | 0 |
| Namibia | 1994 - 2000 | -0.22 (-0.29 to -0.14) | 0 |
| Namibia | 2000 - 2005 | 0.12 (0.02 to 0.22) | 0.027 |
| Namibia | 2005 - 2011 | -0.48 (-0.51 to -0.44) | 0 |
| Namibia | 2014 - 2019 | -1 (-1.32 to -0.68) | 0 |
| Namibia | 2017 - 1995 | -0.07 (-0.43 to 0.28) | 0.671 |
| Nauru | 1990 - 2000 | 0.34 (0.32 to 0.36) | 0 |
| Nauru | 2000 - 2005 | -0.05 (-0.13 to 0.04) | 0.281 |
| Nauru | 2005 - 2010 | 0.26 (0.17 to 0.35) | 0 |
| Nauru | 2010 - 2017 | -0.28 (-0.42 to -0.14) | 0.001 |
| Nauru | 2014 - 2019 | 0 (-0.06 to 0.06) | 0.943 |
| Nepal | 1990 - 1995 | -0.08 (-0.11 to -0.05) | 0 |
| Nepal | 1996 - 2000 | -0.84 (-1.01 to -0.68) | 0 |
| Nepal | 1999 - 2005 | -0.19 (-0.23 to -0.16) | 0 |
| Nepal | 2005 - 2011 | 0.46 (0.4 to 0.51) | 0 |
| Nepal | 2010 - 2015 | -0.98 (-1.01 to -0.95) | 0 |
| Nepal | 2017 - 2019 | 0.09 (-0.09 to 0.27) | 0.312 |
| Netherlands | 1990 - 1993 | 0.58 (0.51 to 0.66) | 0 |
| Netherlands | 1994 - 2000 | -0.26 (-0.27 to -0.24) | 0 |
| Netherlands | 2005 - 2006 | -0.38 (-0.49 to -0.26) | 0 |
| Netherlands | 2009 - 2011 | -0.17 (-0.22 to -0.11) | 0 |
| Netherlands | 2015 - 2014 | 0.08 (0 to 0.16) | 0.04 |
| New Zealand | 1990 - 2019 | -1.9 (-1.94 to -1.86) | 0 |
| New Zealand | 1999 - 1995 | -1.06 (-1.16 to -0.95) | 0 |
| New Zealand | 2005 - 2000 | 0.38 (0.27 to 0.49) | 0 |
| New Zealand | 2011 - 2005 | -0.92 (-1.4 to -0.45) | 0.001 |
| New Zealand | 2014 - 2012 | 1.72 (1.24 to 2.2) | 0 |
| New Zealand | 2017 - 2017 | 0.24 (-0.27 to 0.76) | 0.324 |
| Nicaragua | 1990 - 2019 | 0.29 (0.28 to 0.31) | 0 |
| Nicaragua | 1996 - 2000 | 0.59 (0.51 to 0.67) | 0 |
| Nicaragua | 1999 - 2005 | 0.22 (0.2 to 0.24) | 0 |
| Nicaragua | 2005 - 2010 | -0.18 (-0.2 to -0.15) | 0 |
| Nicaragua | 2010 - 2014 | -0.57 (-0.59 to -0.54) | 0 |
| Nicaragua | 2015 - 2019 | 0.16 (0.14 to 0.19) | 0 |
| Niger | 1990 - 2000 | -0.05 (-0.12 to 0.03) | 0.201 |
| Niger | 1995 - 2005 | 0.69 (0.58 to 0.79) | 0 |
| Niger | 2000 - 2011 | -0.29 (-0.4 to -0.19) | 0 |
| Niger | 2005 - 2019 | 0.53 (0.42 to 0.63) | 0 |
| Niger | 2010 - 1995 | -0.04 (-0.21 to 0.13) | 0.616 |
| Niger | 2014 - 2000 | 0.19 (0.11 to 0.26) | 0 |
| Nigeria | 1990 - 2005 | 0.48 (0.31 to 0.64) | 0 |
| Nigeria | 1993 - 2010 | 0.81 (0.5 to 1.12) | 0 |
| Nigeria | 1996 - 2017 | 1.87 (1.54 to 2.2) | 0 |
| Nigeria | 1999 - 2019 | 0.54 (0.21 to 0.88) | 0.003 |
| Nigeria | 2002 - 1995 | 0.08 (0.06 to 0.09) | 0 |
| Niue | 1990 - 2000 | 0.47 (0.39 to 0.54) | 0 |
| Niue | 1993 - 2005 | 0.29 (0.26 to 0.33) | 0 |
| Niue | 1999 - 2011 | 0.07 (0.06 to 0.08) | 0 |
| Niue | 2011 - 2015 | -0.17 (-0.32 to -0.03) | 0.023 |
| Niue | 2014 - 2019 | -0.03 (-0.18 to 0.12) | 0.646 |
| Niue | 2017 - 1993 | -0.23 (-0.37 to -0.08) | 0.006 |
| North Macedonia | 1990 - 2000 | -0.16 (-0.17 to -0.14) | 0 |
| North Macedonia | 2001 - 2006 | -0.37 (-0.61 to -0.12) | 0.006 |
| North Macedonia | 2004 - 2011 | -0.14 (-0.2 to -0.09) | 0 |
| North Macedonia | 2010 - 2014 | -1.11 (-1.22 to -0.99) | 0 |
| North Macedonia | 2014 - 2019 | 0.09 (0.04 to 0.14) | 0.002 |
| Northern Mariana Islands | 1990 - 1995 | 1.04 (0.97 to 1.11) | 0 |
| Northern Mariana Islands | 1993 - 2000 | 0.85 (0.82 to 0.88) | 0 |
| Northern Mariana Islands | 1999 - 2005 | 0.13 (0.11 to 0.16) | 0 |
| Northern Mariana Islands | 2006 - 2012 | 0.42 (0.29 to 0.56) | 0 |
| Northern Mariana Islands | 2009 - 2017 | 0.05 (0.04 to 0.06) | 0 |
| Norway | 1990 - 2019 | -0.96 (-1.02 to -0.9) | 0 |
| Norway | 2000 - 2000 | -0.45 (-0.7 to -0.21) | 0.001 |
| Norway | 2005 - 2005 | 0.68 (0.44 to 0.93) | 0 |
| Norway | 2010 - 2010 | -0.49 (-0.87 to -0.1) | 0.017 |
| Norway | 2014 - 2014 | 0.47 (0.29 to 0.64) | 0 |
| Oman | 1990 - 2019 | -0.23 (-0.43 to -0.03) | 0.027 |
| Oman | 1995 - 2000 | 0.45 (0.24 to 0.65) | 0 |
| Oman | 2001 - 2005 | 1.23 (1.14 to 1.33) | 0 |
| Oman | 2010 - 2011 | -0.62 (-1.07 to -0.16) | 0.011 |
| Oman | 2014 - 2019 | 0.64 (0.43 to 0.85) | 0 |
| Pakistan | 1990 - 1995 | -0.15 (-0.2 to -0.1) | 0 |
| Pakistan | 1995 - 2000 | 0.15 (0.12 to 0.19) | 0 |
| Pakistan | 2002 - 2005 | 0.49 (0.28 to 0.7) | 0 |
| Pakistan | 2005 - 2010 | 1.33 (1.25 to 1.4) | 0 |
| Pakistan | 2010 - 2017 | -0.75 (-0.82 to -0.68) | 0 |
| Pakistan | 2015 - 2019 | 0.06 (-0.01 to 0.13) | 0.101 |
| Palau | 1990 - 1995 | 0.41 (0.4 to 0.42) | 0 |
| Palau | 2000 - 2000 | -0.1 (-0.14 to -0.06) | 0 |
| Palau | 2005 - 2005 | 0.27 (0.23 to 0.31) | 0 |
| Palau | 2010 - 2011 | -0.21 (-0.27 to -0.14) | 0 |
| Palau | 2014 - 2015 | 0.08 (-0.05 to 0.2) | 0.202 |
| Palau | 2017 - 2019 | -0.44 (-0.57 to -0.31) | 0 |
| Palestine | 1990 - 1993 | -0.22 (-0.3 to -0.14) | 0 |
| Palestine | 1995 - 2000 | 0.35 (0.24 to 0.46) | 0 |
| Palestine | 2000 - 2006 | -0.25 (-0.36 to -0.14) | 0 |
| Palestine | 2005 - 2011 | 0.66 (0.55 to 0.77) | 0 |
| Palestine | 2010 - 2014 | -0.6 (-0.77 to -0.42) | 0 |
| Palestine | 2014 - 2019 | 0.12 (0.04 to 0.2) | 0.006 |
| Panama | 1990 - 1995 | 0.18 (0.16 to 0.2) | 0 |
| Panama | 1997 - 2000 | 0.36 (0.34 to 0.38) | 0 |
| Panama | 2005 - 2005 | -0.14 (-0.19 to -0.09) | 0 |
| Panama | 2010 - 2012 | -0.08 (-0.1 to -0.07) | 0 |
| Papua New Guinea | 1990 - 2017 | 0.15 (0.09 to 0.21) | 0 |
| Papua New Guinea | 1995 - 2019 | 0.79 (0.7 to 0.88) | 0 |
| Papua New Guinea | 2000 - 2000 | 0.19 (0.16 to 0.21) | 0 |
| Papua New Guinea | 2010 - 2005 | -0.46 (-0.54 to -0.37) | 0 |
| Papua New Guinea | 2015 - 2010 | -0.01 (-0.1 to 0.08) | 0.802 |
| Paraguay | 1990 - 2014 | 0.47 (0.41 to 0.53) | 0 |
| Paraguay | 1994 - 2019 | 0.23 (0.21 to 0.24) | 0 |
| Paraguay | 2005 - 2000 | 0.75 (0.69 to 0.8) | 0 |
| Paraguay | 2010 - 2005 | -0.43 (-0.49 to -0.38) | 0 |
| Paraguay | 2015 - 2011 | 0.14 (0.08 to 0.19) | 0 |
| Peru | 1990 - 2019 | 0.05 (-0.07 to 0.16) | 0.388 |
| Peru | 1995 - 1995 | -0.9 (-1.06 to -0.74) | 0 |
| Peru | 2000 - 2000 | -0.13 (-0.29 to 0.02) | 0.084 |
| Peru | 2005 - 2005 | 0.21 (0.16 to 0.27) | 0 |
| Peru | 2014 - 2010 | 0.35 (0.24 to 0.46) | 0 |
| Philippines | 1990 - 2017 | 1.47 (1.42 to 1.52) | 0 |
| Philippines | 1995 - 2019 | 2.29 (2.18 to 2.4) | 0 |
| Philippines | 1999 - 1995 | 0.08 (-0.16 to 0.32) | 0.483 |
| Philippines | 2002 - 2000 | -0.38 (-0.49 to -0.27) | 0 |
| Philippines | 2006 - 2005 | -0.71 (-0.83 to -0.6) | 0 |
| Philippines | 2010 - 2011 | 0.08 (0.06 to 0.1) | 0 |
| Poland | 1990 - 2015 | -0.53 (-0.81 to -0.26) | 0.001 |
| Poland | 1994 - 2019 | 0.4 (0.34 to 0.46) | 0 |
| Poland | 2006 - 1993 | -0.43 (-0.85 to 0) | 0.049 |
| Poland | 2010 - 2000 | -2.49 (-2.89 to -2.08) | 0 |
| Poland | 2014 - 2006 | 0.4 (0.21 to 0.58) | 0 |
| Portugal | 1990 - 2011 | -2.04 (-2.47 to -1.6) | 0 |
| Portugal | 1995 - 2014 | 2.48 (1.85 to 3.12) | 0 |
| Portugal | 2000 - 2019 | 0.81 (0.16 to 1.45) | 0.018 |
| Portugal | 2005 - 1995 | 2.36 (1.73 to 2.99) | 0 |
| Portugal | 2010 - 2000 | -1.47 (-2.44 to -0.49) | 0.007 |
| Portugal | 2014 - 2005 | 1.17 (0.71 to 1.63) | 0 |
| Puerto Rico | 1990 - 2012 | 0.25 (0.23 to 0.27) | 0 |
| Puerto Rico | 1996 - 2017 | 0.39 (0.27 to 0.52) | 0 |
| Puerto Rico | 1999 - 2019 | 0.05 (-0.09 to 0.18) | 0.476 |
| Puerto Rico | 2002 - 2000 | -0.05 (-0.07 to -0.04) | 0 |
| Puerto Rico | 2010 - 2005 | -0.3 (-0.32 to -0.28) | 0 |
| Puerto Rico | 2017 - 2010 | 0.05 (-0.09 to 0.19) | 0.451 |
| Qatar | 1990 - 2014 | 0.22 (0.16 to 0.27) | 0 |
| Qatar | 1994 - 2019 | -0.08 (-0.17 to 0) | 0.056 |
| Qatar | 1998 - 2000 | -0.31 (-0.49 to -0.12) | 0.004 |
| Qatar | 2001 - 2005 | -0.68 (-0.77 to -0.6) | 0 |
| Qatar | 2005 - 2011 | -0.4 (-0.41 to -0.39) | 0 |
| Qatar | 2017 - 2019 | 0.02 (-0.17 to 0.21) | 0.826 |
| Republic of Korea | 1990 - 1995 | 0.47 (0.1 to 0.85) | 0.018 |
| Republic of Korea | 1995 - 2000 | 1.89 (1.37 to 2.42) | 0 |
| Republic of Korea | 2000 - 2005 | -1.63 (-2.14 to -1.13) | 0 |
| Republic of Korea | 2005 - 2010 | 1.59 (1.04 to 2.15) | 0 |
| Republic of Korea | 2010 - 2017 | -3.17 (-3.68 to -2.66) | 0 |
| Republic of Korea | 2015 - 2019 | 2.22 (1.65 to 2.79) | 0 |
| Republic of Moldova | 1990 - 1995 | 0.22 (0.2 to 0.24) | 0 |
| Republic of Moldova | 2000 - 2000 | -0.07 (-0.17 to 0.02) | 0.127 |
| Republic of Moldova | 2005 - 2005 | 0.9 (0.8 to 0.99) | 0 |
| Republic of Moldova | 2010 - 2011 | -0.09 (-0.24 to 0.06) | 0.21 |
| Republic of Moldova | 2014 - 2015 | 0.61 (0.31 to 0.91) | 0.001 |
| Republic of Moldova | 2017 - 2019 | 0.06 (-0.25 to 0.37) | 0.7 |
| Romania | 1990 - 1993 | 0.18 (-0.03 to 0.39) | 0.093 |
| Romania | 1996 - 2000 | 0.87 (-0.35 to 2.1) | 0.15 |
| Romania | 1999 - 2006 | -0.48 (-0.56 to -0.4) | 0 |
| Romania | 2011 - 2011 | -1.09 (-2.24 to 0.07) | 0.064 |
| Romania | 2014 - 2014 | 0.69 (0.43 to 0.95) | 0 |
| Russian Federation | 1990 - 2019 | 0.49 (0.36 to 0.63) | 0 |
| Russian Federation | 2001 - 1995 | -0.2 (-1.17 to 0.79) | 0.676 |
| Russian Federation | 2005 - 2000 | 0.77 (0.15 to 1.4) | 0.018 |
| Russian Federation | 2010 - 2005 | -0.89 (-1.86 to 0.08) | 0.069 |
| Russian Federation | 2014 - 2012 | 1.37 (0.92 to 1.81) | 0 |
| Rwanda | 1990 - 2017 | 0.43 (0.29 to 0.56) | 0 |
| Rwanda | 1994 - 2019 | -0.09 (-0.16 to -0.02) | 0.021 |
| Rwanda | 2001 - 2000 | -0.43 (-0.48 to -0.39) | 0 |
| Rwanda | 2010 - 2005 | 0.26 (0.05 to 0.47) | 0.019 |
| Rwanda | 2014 - 2010 | -0.22 (-0.32 to -0.13) | 0 |
| Saint Kitts and Nevis | 1990 - 2014 | -0.05 (-0.1 to -0.01) | 0.021 |
| Saint Kitts and Nevis | 1995 - 2019 | 0.19 (0.13 to 0.25) | 0 |
| Saint Kitts and Nevis | 2000 - 2000 | -0.06 (-0.12 to 0) | 0.051 |
| Saint Kitts and Nevis | 2005 - 2005 | 0.04 (-0.02 to 0.1) | 0.172 |
| Saint Kitts and Nevis | 2010 - 2011 | -0.19 (-0.29 to -0.09) | 0.001 |
| Saint Kitts and Nevis | 2014 - 2019 | 0.12 (0.08 to 0.17) | 0 |
| Saint Lucia | 1990 - 1995 | -0.01 (-0.02 to 0.01) | 0.18 |
| Saint Lucia | 1995 - 2000 | 0.18 (0.16 to 0.2) | 0 |
| Saint Lucia | 2000 - 2005 | -0.3 (-0.32 to -0.28) | 0 |
| Saint Lucia | 2005 - 2010 | -0.01 (-0.03 to 0.01) | 0.302 |
| Saint Lucia | 2010 - 2017 | -0.29 (-0.31 to -0.27) | 0 |
| Saint Lucia | 2015 - 2019 | 0.11 (0.09 to 0.13) | 0 |
| Saint Vincent and the Grenadines | 1990 - 1995 | -0.38 (-0.51 to -0.24) | 0 |
| Saint Vincent and the Grenadines | 1993 - 2000 | -0.25 (-0.29 to -0.21) | 0 |
| Saint Vincent and the Grenadines | 2000 - 2005 | 0.14 (0.09 to 0.2) | 0 |
| Saint Vincent and the Grenadines | 2006 - 2011 | 0.25 (0.12 to 0.39) | 0.001 |
| Saint Vincent and the Grenadines | 2010 - 2015 | -0.29 (-0.42 to -0.16) | 0 |
| Saint Vincent and the Grenadines | 2014 - 2019 | 0.16 (0.1 to 0.22) | 0 |
| Samoa | 1990 - 1993 | 0.02 (-0.04 to 0.08) | 0.508 |
| Samoa | 1995 - 2000 | 0.48 (0.4 to 0.57) | 0 |
| Samoa | 2000 - 2006 | -0.24 (-0.25 to -0.22) | 0 |
| Samoa | 2014 - 2011 | 0.08 (0.02 to 0.14) | 0.014 |
| San Marino | 1990 - 2014 | -0.3 (-0.33 to -0.28) | 0 |
| San Marino | 2005 - 2019 | 0.42 (0.23 to 0.61) | 0 |
| San Marino | 2010 - 1995 | -0.46 (-0.76 to -0.17) | 0.004 |
| San Marino | 2014 - 2000 | 0.48 (0.34 to 0.62) | 0 |
| Sao Tome and Principe | 1990 - 2005 | 0.31 (0.22 to 0.4) | 0 |
| Sao Tome and Principe | 1995 - 2012 | 0.9 (0.77 to 1.03) | 0 |
| Sao Tome and Principe | 2000 - 2017 | 0.09 (-0.04 to 0.22) | 0.161 |
| Sao Tome and Principe | 2005 - 2019 | 0.43 (0.3 to 0.56) | 0 |
| Sao Tome and Principe | 2010 - 2000 | -0.21 (-0.41 to -0.01) | 0.042 |
| Sao Tome and Principe | 2014 - 2005 | 0.14 (0.05 to 0.23) | 0.005 |
| Saudi Arabia | 1990 - 2010 | 0.47 (0.38 to 0.57) | 0 |
| Saudi Arabia | 1995 - 2014 | 1.24 (1.11 to 1.38) | 0 |
| Saudi Arabia | 2000 - 2019 | 0.32 (0.23 to 0.42) | 0 |
| Saudi Arabia | 2006 - 2000 | 0.82 (0.61 to 1.03) | 0 |
| Saudi Arabia | 2010 - 2005 | -0.72 (-0.92 to -0.51) | 0 |
| Saudi Arabia | 2014 - 2011 | 0.14 (0.05 to 0.24) | 0.007 |
| Senegal | 1990 - 2019 | -0.14 (-0.2 to -0.08) | 0 |
| Senegal | 1995 - 1995 | 0.52 (0.44 to 0.6) | 0 |
| Senegal | 2000 - 2000 | -0.56 (-0.64 to -0.48) | 0 |
| Senegal | 2005 - 2005 | 0.81 (0.74 to 0.89) | 0 |
| Senegal | 2010 - 2010 | -0.62 (-0.7 to -0.54) | 0 |
| Senegal | 2015 - 2017 | 0.19 (0.11 to 0.28) | 0 |
| Serbia | 1990 - 2019 | 1.05 (0.81 to 1.29) | 0 |
| Serbia | 1992 - 1995 | 0.58 (0.36 to 0.8) | 0 |
| Serbia | 1995 - 2000 | 0.13 (0.11 to 0.15) | 0 |
| Serbia | 2005 - 2005 | -0.05 (-0.1 to 0) | 0.044 |
| Serbia | 2011 - 2011 | 0.18 (-0.03 to 0.4) | 0.092 |
| Serbia | 2014 - 2015 | -0.09 (-0.14 to -0.04) | 0.001 |
| Seychelles | 1990 - 2019 | -0.4 (-0.53 to -0.26) | 0 |
| Seychelles | 1994 - 1993 | 0.71 (0.61 to 0.81) | 0 |
| Seychelles | 2000 - 2000 | -0.75 (-0.88 to -0.61) | 0 |
| Seychelles | 2005 - 2006 | -0.06 (-0.2 to 0.07) | 0.335 |
| Seychelles | 2010 - 2011 | -0.87 (-1.01 to -0.74) | 0 |
| Seychelles | 2015 - 2014 | 0.4 (0.26 to 0.54) | 0 |
| Sierra Leone | 1990 - 2019 | -0.3 (-0.46 to -0.14) | 0.001 |
| Sierra Leone | 1995 - 1995 | 1.18 (0.95 to 1.41) | 0 |
| Sierra Leone | 2000 - 2000 | -0.62 (-0.84 to -0.39) | 0 |
| Sierra Leone | 2005 - 2005 | 0.49 (0.25 to 0.73) | 0.001 |
| Sierra Leone | 2010 - 2012 | -0.78 (-1.15 to -0.41) | 0.001 |
| Sierra Leone | 2014 - 2017 | 0.32 (0.16 to 0.49) | 0.001 |
| Singapore | 1990 - 2019 | -1.06 (-1.21 to -0.91) | 0 |
| Singapore | 1993 - 2000 | -0.81 (-0.85 to -0.77) | 0 |
| Singapore | 2001 - 2005 | -1.8 (-2.07 to -1.52) | 0 |
| Singapore | 2004 - 2010 | -0.72 (-0.75 to -0.7) | 0 |
| Singapore | 2014 - 2014 | -1.58 (-1.85 to -1.31) | 0 |
| Singapore | 2017 - 2019 | -0.22 (-0.52 to 0.08) | 0.136 |
| Slovakia | 1990 - 2000 | -1.23 (-1.37 to -1.09) | 0 |
| Slovakia | 1994 - 2005 | -0.24 (-0.34 to -0.14) | 0 |
| Slovakia | 2000 - 2011 | -1.41 (-1.43 to -1.39) | 0 |
| Slovakia | 2014 - 2019 | 0.11 (0.01 to 0.2) | 0.03 |
| Slovenia | 1990 - 1995 | -0.54 (-0.64 to -0.44) | 0 |
| Slovenia | 1993 - 2000 | -0.38 (-0.4 to -0.35) | 0 |
| Slovenia | 2001 - 2005 | -1.28 (-1.31 to -1.26) | 0 |
| Slovenia | 2009 - 2010 | -1.11 (-1.31 to -0.92) | 0 |
| Slovenia | 2012 - 2017 | -0.76 (-0.95 to -0.57) | 0 |
| Slovenia | 2015 - 2019 | 0.11 (0.05 to 0.17) | 0.002 |
| Solomon Islands | 1990 - 1995 | 0.12 (0.05 to 0.19) | 0.002 |
| Solomon Islands | 1996 - 2000 | 0.27 (0.08 to 0.47) | 0.01 |
| Solomon Islands | 2000 - 2005 | -0.35 (-0.47 to -0.22) | 0 |
| Solomon Islands | 2005 - 2011 | 0.25 (0.13 to 0.38) | 0.001 |
| Solomon Islands | 2010 - 2015 | -0.4 (-0.59 to -0.21) | 0.001 |
| Solomon Islands | 2014 - 2019 | 0.16 (0.07 to 0.25) | 0.002 |
| Somalia | 1990 - 1993 | 0.41 (0.39 to 0.43) | 0 |
| Somalia | 1996 - 2000 | 0.55 (0.49 to 0.61) | 0 |
| Somalia | 2000 - 2006 | 0.07 (0.03 to 0.11) | 0.001 |
| Somalia | 2005 - 2011 | 0.35 (0.31 to 0.38) | 0 |
| Somalia | 2010 - 2014 | -0.33 (-0.37 to -0.3) | 0 |
| Somalia | 2015 - 2019 | 0.16 (0.12 to 0.19) | 0 |
| South Africa | 1990 - 1995 | -0.32 (-0.42 to -0.21) | 0 |
| South Africa | 1995 - 2000 | 0.67 (0.52 to 0.82) | 0 |
| South Africa | 2000 - 2005 | -1.59 (-1.74 to -1.45) | 0 |
| South Africa | 2005 - 2012 | -1.01 (-1.46 to -0.57) | 0 |
| South Africa | 2008 - 2017 | -0.6 (-0.65 to -0.55) | 0 |
| South Africa | 2017 - 2019 | -0.16 (-0.66 to 0.33) | 0.487 |
| South Sudan | 1990 - 2000 | 0.91 (0.84 to 0.98) | 0 |
| South Sudan | 1994 - 2005 | 0.53 (0.49 to 0.57) | 0 |
| South Sudan | 2001 - 2010 | 0.62 (0.4 to 0.84) | 0 |
| South Sudan | 2004 - 2014 | 0.28 (0.17 to 0.39) | 0 |
| South Sudan | 2008 - 2019 | 0.13 (0.08 to 0.18) | 0 |
| South Sudan | 2014 - 2000 | -0.02 (-0.07 to 0.03) | 0.501 |
| Spain | 1990 - 2005 | 0.49 (0.27 to 0.71) | 0 |
| Spain | 1995 - 2011 | -0.82 (-0.98 to -0.66) | 0 |
| Spain | 2002 - 2019 | -0.35 (-1.2 to 0.51) | 0.397 |
| Spain | 2005 - 1995 | 1.1 (0.81 to 1.39) | 0 |
| Spain | 2010 - 2000 | -0.31 (-0.76 to 0.14) | 0.156 |
| Spain | 2014 - 2005 | 0.62 (0.42 to 0.83) | 0 |
| Sri Lanka | 1990 - 2010 | -0.77 (-0.94 to -0.6) | 0 |
| Sri Lanka | 1993 - 2017 | -0.34 (-0.39 to -0.3) | 0 |
| Sri Lanka | 2001 - 2019 | -0.66 (-0.82 to -0.5) | 0 |
| Sri Lanka | 2005 - 1995 | 1.53 (1.43 to 1.64) | 0 |
| Sri Lanka | 2010 - 2000 | -0.89 (-1 to -0.78) | 0 |
| Sri Lanka | 2015 - 2005 | 0.16 (0.05 to 0.27) | 0.007 |
| Sudan | 1990 - 2011 | 0.02 (-0.2 to 0.23) | 0.872 |
| Sudan | 1995 - 2015 | 0.92 (0.62 to 1.22) | 0 |
| Sudan | 2000 - 2019 | -0.2 (-0.5 to 0.1) | 0.171 |
| Sudan | 2005 - 1993 | 0.91 (0.61 to 1.21) | 0 |
| Sudan | 2010 - 2000 | -0.59 (-1.06 to -0.12) | 0.018 |
| Sudan | 2014 - 2006 | 0.58 (0.36 to 0.8) | 0 |
| Suriname | 1990 - 2011 | 0.31 (0.18 to 0.44) | 0 |
| Suriname | 1995 - 2014 | 0.97 (0.78 to 1.15) | 0 |
| Suriname | 2000 - 2019 | 0.27 (0.15 to 0.4) | 0 |
| Suriname | 2006 - 1995 | 0.64 (0.35 to 0.93) | 0 |
| Suriname | 2010 - 2000 | -0.45 (-0.73 to -0.16) | 0.005 |
| Suriname | 2014 - 2005 | 0.44 (0.31 to 0.57) | 0 |
| Sweden | 1990 - 2012 | -0.19 (-0.24 to -0.14) | 0 |
| Sweden | 2000 - 2017 | 0.35 (0.16 to 0.55) | 0.001 |
| Sweden | 2005 - 2019 | 1.33 (1.03 to 1.63) | 0 |
| Sweden | 2009 - 2000 | 0.4 (0.35 to 0.45) | 0 |
| Switzerland | 1990 - 2005 | -0.69 (-0.86 to -0.52) | 0 |
| Switzerland | 1995 - 2010 | -0.03 (-0.1 to 0.03) | 0.269 |
| Switzerland | 2005 - 2014 | 0.62 (0.4 to 0.84) | 0 |
| Switzerland | 2010 - 2019 | -0.79 (-1.13 to -0.45) | 0 |
| Switzerland | 2014 - 2000 | 0.46 (0.3 to 0.62) | 0 |
| Syrian Arab Republic | 1990 - 2005 | -0.03 (-0.3 to 0.23) | 0.794 |
| Syrian Arab Republic | 1995 - 2011 | 0.77 (0.41 to 1.13) | 0 |
| Syrian Arab Republic | 2000 - 2019 | -0.52 (-0.88 to -0.16) | 0.008 |
| Syrian Arab Republic | 2005 - 1995 | 1.03 (0.65 to 1.4) | 0 |
| Syrian Arab Republic | 2010 - 2000 | -0.5 (-1.09 to 0.09) | 0.088 |
| Syrian Arab Republic | 2014 - 2005 | 0.71 (0.44 to 0.98) | 0 |
| Taiwan (Province of China) | 1990 - 2010 | -0.58 (-0.74 to -0.42) | 0 |
| Taiwan (Province of China) | 1994 - 2017 | 0.22 (-0.28 to 0.72) | 0.357 |
| Taiwan (Province of China) | 1997 - 2019 | 0.92 (0.85 to 0.99) | 0 |
| Taiwan (Province of China) | 2005 - 1995 | 0 (-0.16 to 0.16) | 0.957 |
| Taiwan (Province of China) | 2010 - 2000 | -1.61 (-1.68 to -1.54) | 0 |
| Taiwan (Province of China) | 2017 - 2005 | -0.08 (-0.47 to 0.31) | 0.674 |
| Tajikistan | 1990 - 2011 | -0.18 (-0.23 to -0.13) | 0 |
| Tajikistan | 2000 - 2015 | -0.55 (-0.75 to -0.35) | 0 |
| Tajikistan | 2005 - 2019 | -0.01 (-0.2 to 0.19) | 0.917 |
| Tajikistan | 2010 - 1993 | -0.32 (-0.63 to -0.01) | 0.041 |
| Tajikistan | 2014 - 2000 | 0.42 (0.28 to 0.56) | 0 |
| Thailand | 1990 - 2006 | -0.5 (-0.59 to -0.4) | 0 |
| Thailand | 1995 - 2011 | 0.75 (0.62 to 0.88) | 0 |
| Thailand | 2000 - 2014 | -0.74 (-0.86 to -0.61) | 0 |
| Thailand | 2005 - 2019 | -0.05 (-0.1 to -0.01) | 0.024 |
| Thailand | 2014 - 1995 | 0.79 (0.38 to 1.21) | 0.001 |
| Thailand | 2017 - 2000 | 0.07 (-0.35 to 0.49) | 0.728 |
| Timor-Leste | 1990 - 2005 | -0.52 (-0.87 to -0.17) | 0.007 |
| Timor-Leste | 1994 - 2012 | 0.31 (0.06 to 0.56) | 0.019 |
| Timor-Leste | 2000 - 2017 | -0.3 (-0.65 to 0.05) | 0.088 |
| Timor-Leste | 2005 - 2019 | 0.51 (0.16 to 0.86) | 0.008 |
| Timor-Leste | 2010 - 2000 | -0.38 (-0.93 to 0.17) | 0.158 |
| Timor-Leste | 2014 - 2005 | 0.6 (0.35 to 0.85) | 0 |
| Togo | 1990 - 2010 | -0.02 (-0.18 to 0.14) | 0.755 |
| Togo | 1995 - 2014 | 1.27 (1.05 to 1.5) | 0 |
| Togo | 2000 - 2019 | -0.23 (-0.46 to -0.01) | 0.046 |
| Togo | 2005 - 2000 | 0.58 (0.34 to 0.81) | 0 |
| Togo | 2010 - 2005 | -0.51 (-0.88 to -0.14) | 0.011 |
| Togo | 2014 - 2011 | 0.26 (0.1 to 0.43) | 0.005 |
| Tokelau | 1990 - 2019 | 0.32 (0.29 to 0.36) | 0 |
| Tokelau | 1995 - 1995 | 0.45 (0.4 to 0.5) | 0 |
| Tokelau | 2000 - 2000 | -0.04 (-0.09 to 0.02) | 0.155 |
| Tokelau | 2005 - 2005 | 0.22 (0.17 to 0.28) | 0 |
| Tokelau | 2010 - 2010 | -0.22 (-0.3 to -0.13) | 0 |
| Tokelau | 2014 - 2017 | -0.11 (-0.15 to -0.07) | 0 |
| Tonga | 1990 - 2019 | 0.71 (0.6 to 0.82) | 0 |
| Tonga | 1994 - 1995 | 0.12 (0.04 to 0.2) | 0.004 |
| Tonga | 2000 - 2000 | -0.23 (-0.35 to -0.12) | 0 |
| Tonga | 2005 - 2005 | 0.33 (0.21 to 0.44) | 0 |
| Tonga | 2010 - 2011 | -0.23 (-0.26 to -0.2) | 0 |
| Trinidad and Tobago | 1990 - 2015 | -0.43 (-0.57 to -0.29) | 0 |
| Trinidad and Tobago | 1995 - 2019 | 0.62 (0.43 to 0.82) | 0 |
| Trinidad and Tobago | 2000 - 1993 | -0.82 (-1.01 to -0.63) | 0 |
| Trinidad and Tobago | 2005 - 2000 | 0.15 (-0.04 to 0.34) | 0.115 |
| Trinidad and Tobago | 2010 - 2006 | -0.7 (-1 to -0.4) | 0 |
| Trinidad and Tobago | 2014 - 2011 | 0.32 (0.18 to 0.46) | 0 |
| Tunisia | 1990 - 2014 | 0.13 (0.08 to 0.17) | 0 |
| Tunisia | 1996 - 2019 | 0.29 (0.16 to 0.42) | 0 |
| Tunisia | 2000 - 1995 | -0.06 (-0.14 to 0.03) | 0.16 |
| Tunisia | 2005 - 2000 | 0.23 (0.15 to 0.32) | 0 |
| Tunisia | 2010 - 2005 | -0.21 (-0.34 to -0.07) | 0.005 |
| Tunisia | 2014 - 2012 | 0.19 (0.13 to 0.25) | 0 |
| Turkey | 1990 - 2017 | -0.91 (-1.03 to -0.78) | 0 |
| Turkey | 1995 - 2019 | 0.8 (0.63 to 0.98) | 0 |
| Turkey | 2000 - 2000 | -1.59 (-1.76 to -1.42) | 0 |
| Turkey | 2005 - 2005 | -0.02 (-0.2 to 0.15) | 0.784 |
| Turkey | 2010 - 2010 | -2.59 (-2.75 to -2.42) | 0 |
| Turkey | 2015 - 2014 | -0.15 (-0.32 to 0.02) | 0.073 |
| Turkmenistan | 1990 - 2019 | -0.46 (-0.78 to -0.14) | 0.008 |
| Turkmenistan | 1993 - 2000 | 0.03 (-0.01 to 0.06) | 0.148 |
| Turkmenistan | 2006 - 2005 | 0.33 (0.01 to 0.65) | 0.044 |
| Turkmenistan | 2010 - 2011 | -0.17 (-0.48 to 0.15) | 0.276 |
| Turkmenistan | 2014 - 2019 | 0.45 (0.31 to 0.59) | 0 |
| Tuvalu | 1990 - 1995 | 0.23 (0.1 to 0.36) | 0.002 |
| Tuvalu | 1995 - 2000 | 0.6 (0.31 to 0.89) | 0.001 |
| Tuvalu | 1999 - 2005 | 0.1 (0.06 to 0.14) | 0 |
| Tuvalu | 2011 - 2010 | -0.44 (-1 to 0.12) | 0.112 |
| Tuvalu | 2014 - 2017 | 0.29 (-0.27 to 0.85) | 0.281 |
| Tuvalu | 2017 - 2019 | -0.31 (-0.88 to 0.25) | 0.253 |
| Uganda | 1990 - 1995 | 0.28 (0.25 to 0.32) | 0 |
| Uganda | 1995 - 2000 | 0.53 (0.48 to 0.58) | 0 |
| Uganda | 2000 - 2005 | -0.07 (-0.12 to -0.02) | 0.007 |
| Uganda | 2005 - 2011 | -0.21 (-0.22 to -0.19) | 0 |
| Uganda | 2014 - 2015 | -0.29 (-0.44 to -0.14) | 0.001 |
| Uganda | 2017 - 2019 | 0 (-0.16 to 0.16) | 0.999 |
| Ukraine | 1990 - 1993 | -0.01 (-0.23 to 0.2) | 0.907 |
| Ukraine | 1995 - 2000 | 1.28 (0.96 to 1.59) | 0 |
| Ukraine | 2000 - 2006 | 0.26 (-0.06 to 0.58) | 0.101 |
| Ukraine | 2005 - 2011 | -0.35 (-0.45 to -0.24) | 0 |
| Ukraine | 2014 - 2014 | 0.61 (0.39 to 0.84) | 0 |
| United Arab Emirates | 1990 - 2019 | -0.08 (-0.09 to -0.06) | 0 |
| United Arab Emirates | 1999 - 1995 | -0.47 (-0.51 to -0.43) | 0 |
| United Arab Emirates | 2005 - 2000 | 1.01 (0.95 to 1.07) | 0 |
| United Arab Emirates | 2010 - 2005 | 0.08 (-0.01 to 0.17) | 0.069 |
| United Arab Emirates | 2014 - 2012 | 0.49 (0.32 to 0.67) | 0 |
| United Arab Emirates | 2017 - 2017 | -0.02 (-0.22 to 0.17) | 0.793 |
| United Kingdom | 1990 - 2019 | -1.14 (-1.57 to -0.71) | 0 |
| United Kingdom | 1993 - 2000 | -0.74 (-0.79 to -0.68) | 0 |
| United Kingdom | 2005 - 2005 | 0.12 (0.03 to 0.2) | 0.012 |
| United Kingdom | 2014 - 2010 | 1.18 (0.4 to 1.97) | 0.005 |
| United Kingdom | 2017 - 2014 | 0.23 (-0.63 to 1.09) | 0.582 |
| United Republic of Tanzania | 1990 - 2019 | 0.33 (0.3 to 0.36) | 0 |
| United Republic of Tanzania | 1996 - 2000 | 0.78 (0.61 to 0.95) | 0 |
| United Republic of Tanzania | 1999 - 2005 | 0.62 (0.59 to 0.65) | 0 |
| United Republic of Tanzania | 2006 - 2011 | 0.85 (0.68 to 1.02) | 0 |
| United Republic of Tanzania | 2009 - 2019 | 0.31 (0.29 to 0.33) | 0 |
| United Republic of Tanzania | 2017 - 1995 | 0.11 (-0.08 to 0.3) | 0.227 |
| United States Virgin Islands | 1990 - 2000 | 0.03 (0.02 to 0.05) | 0 |
| United States Virgin Islands | 1995 - 2005 | -0.06 (-0.08 to -0.04) | 0 |
| United States Virgin Islands | 2000 - 2010 | 0.11 (0.09 to 0.13) | 0 |
| United States Virgin Islands | 2005 - 2017 | -0.27 (-0.29 to -0.25) | 0 |
| United States Virgin Islands | 2010 - 2019 | 0.27 (0.25 to 0.29) | 0 |
| United States Virgin Islands | 2015 - 1995 | -0.11 (-0.13 to -0.09) | 0 |
| United States of America | 1990 - 2000 | -1.64 (-1.76 to -1.52) | 0 |
| United States of America | 1996 - 2005 | -2.66 (-3.01 to -2.31) | 0 |
| United States of America | 2000 - 2011 | 1.41 (1.35 to 1.46) | 0 |
| United States of America | 2011 - 2015 | 0.08 (-0.58 to 0.75) | 0.794 |
| United States of America | 2014 - 2019 | 1.82 (1.17 to 2.47) | 0 |
| United States of America | 2017 - 1993 | 0.24 (-0.48 to 0.96) | 0.493 |
| Uruguay | 1990 - 2000 | -0.69 (-0.89 to -0.48) | 0 |
| Uruguay | 1995 - 2006 | 0.18 (-0.1 to 0.47) | 0.183 |
| Uruguay | 2000 - 2011 | -1.15 (-1.42 to -0.88) | 0 |
| Uruguay | 2005 - 2014 | 0.01 (-0.19 to 0.21) | 0.908 |
| Uruguay | 2011 - 2019 | -1.35 (-2.21 to -0.48) | 0.005 |
| Uruguay | 2014 - 1995 | 0.44 (0.24 to 0.65) | 0 |
| Uzbekistan | 1990 - 2000 | 0.2 (0.18 to 0.22) | 0 |
| Uzbekistan | 2008 - 2005 | -0.26 (-0.37 to -0.15) | 0 |
| Uzbekistan | 2015 - 2012 | 0.72 (0.51 to 0.93) | 0 |
| Vanuatu | 1990 - 2017 | -0.15 (-0.28 to -0.03) | 0.021 |
| Vanuatu | 1994 - 2019 | 0.07 (-0.02 to 0.16) | 0.123 |
| Vanuatu | 2000 - 2000 | -0.16 (-0.29 to -0.04) | 0.015 |
| Vanuatu | 2005 - 2005 | 0.26 (0.14 to 0.39) | 0.001 |
| Vanuatu | 2010 - 2010 | -0.46 (-0.66 to -0.26) | 0 |
| Vanuatu | 2014 - 2014 | 0.06 (-0.03 to 0.15) | 0.175 |
| Venezuela (Bolivarian Republic of) | 1990 - 2019 | -0.56 (-0.64 to -0.48) | 0 |
| Venezuela (Bolivarian Republic of) | 1999 - 2000 | -0.87 (-1.06 to -0.68) | 0 |
| Venezuela (Bolivarian Republic of) | 2005 - 2005 | -0.29 (-0.48 to -0.1) | 0.005 |
| Venezuela (Bolivarian Republic of) | 2011 - 2011 | -0.88 (-1.71 to -0.03) | 0.042 |
| Venezuela (Bolivarian Republic of) | 2014 - 2019 | 0.62 (0.42 to 0.81) | 0 |
| Viet Nam | 1990 - 1995 | -1.05 (-1.55 to -0.55) | 0.001 |
| Viet Nam | 1992 - 2000 | -0.54 (-0.6 to -0.47) | 0 |
| Viet Nam | 2000 - 2005 | -0.01 (-0.15 to 0.14) | 0.935 |
| Viet Nam | 2005 - 2010 | 1.47 (1.31 to 1.62) | 0 |
| Viet Nam | 2010 - 2017 | -0.68 (-0.84 to -0.52) | 0 |
| Viet Nam | 2015 - 2019 | 0.33 (0.17 to 0.49) | 0.001 |
| Yemen | 1990 - 1995 | -0.27 (-0.5 to -0.05) | 0.022 |
| Yemen | 1995 - 2000 | 0.57 (0.25 to 0.89) | 0.002 |
| Yemen | 2000 - 2005 | -0.45 (-0.77 to -0.13) | 0.01 |
| Yemen | 2005 - 2011 | 0.86 (0.53 to 1.19) | 0 |
| Yemen | 2010 - 2015 | -0.36 (-0.87 to 0.16) | 0.158 |
| Yemen | 2014 - 2019 | 0.65 (0.42 to 0.88) | 0 |
| Zambia | 1990 - 1993 | 0.48 (0.4 to 0.56) | 0 |
| Zambia | 1994 - 2000 | -0.05 (-0.1 to 0) | 0.059 |
| Zambia | 2000 - 2006 | -0.67 (-0.74 to -0.6) | 0 |
| Zambia | 2005 - 2011 | 0.87 (0.8 to 0.94) | 0 |
| Zambia | 2010 - 2014 | -0.3 (-0.41 to -0.18) | 0 |
| Zambia | 2014 - 2019 | 0.04 (-0.02 to 0.09) | 0.185 |
| Zimbabwe | 1990 - 1995 | 0.78 (0.75 to 0.81) | 0 |
| Zimbabwe | 1995 - 2000 | 1.26 (1.2 to 1.32) | 0 |
| Zimbabwe | 1999 - 2005 | 0.57 (0.51 to 0.63) | 0 |
| Zimbabwe | 2003 - 2012 | 0.16 (0.14 to 0.18) | 0 |
| Zimbabwe | 2010 - 2017 | -0.74 (-0.78 to -0.71) | 0 |
| Zimbabwe | 2015 - 2019 | -0.08 (-0.12 to -0.04) | 0.001 |

**Table S5.** ASDR of IHD in 1990 and 2019 for WCBA in 204 countries, with AAPC from 1990 and 2019. Abbreviations: ASDR, age-standardized death rate; IHD, ischemic heart disease; WCBA, women of childbearing age; UI, uncertainty interval; AAPC, average annual percent change; CI, confidence interval.

| location | ASDR, per 100,000 (95% UI) | | AAPC (95% CI) |
| --- | --- | --- | --- |
|  | 1990 | 2019 |  |
| Afghanistan | 73.16 (42.49 to 119.6) | 51.14 (30.41 to 82.74) | -1.18 (-1.28 to -1.08) |
| Albania | 7.13 (5.55 to 8.92) | 5.56 (3.48 to 8.33) | -1.12 (-1.49 to -0.76) |
| Algeria | 34.46 (22.61 to 49.46) | 15 (9.52 to 21.78) | -2.81 (-2.94 to -2.67) |
| American Samoa | 8.73 (5.54 to 12.81) | 13 (8.41 to 18.68) | 1.4 (1.15 to 1.64) |
| Andorra | 2.24 (1.29 to 3.56) | 1.31 (0.75 to 2.06) | -1.77 (-1.88 to -1.66) |
| Angola | 7.57 (3.87 to 12.78) | 5.72 (2.89 to 9.91) | -0.96 (-1.31 to -0.61) |
| Antigua and Barbuda | 7.34 (5.64 to 9.37) | 3.24 (2.25 to 4.41) | -2.76 (-3.07 to -2.45) |
| Argentina | 7.43 (6.43 to 8.55) | 3.24 (2.59 to 3.98) | -2.82 (-3.14 to -2.5) |
| Armenia | 6.77 (5.52 to 8.04) | 4.35 (3.21 to 5.64) | -1.4 (-2.24 to -0.56) |
| Australia | 3.5 (3.04 to 4.01) | 1.6 (1.31 to 1.92) | -2.59 (-3.11 to -2.07) |
| Austria | 4.01 (3.47 to 4.6) | 1.36 (1.09 to 1.66) | -3.61 (-4.53 to -2.68) |
| Azerbaijan | 15.23 (12.47 to 18.26) | 9.97 (6.33 to 14.59) | -1.48 (-2.34 to -0.62) |
| Bahamas | 10.04 (7.8 to 12.78) | 5.6 (3.83 to 7.97) | -2.09 (-2.48 to -1.7) |
| Bahrain | 16.9 (12.64 to 22.13) | 4.28 (2.9 to 6.03) | -4.37 (-5.86 to -2.85) |
| Bangladesh | 18.39 (11.22 to 27.16) | 10.4 (6.67 to 15.29) | -1.83 (-2.21 to -1.44) |
| Barbados | 5.79 (4.55 to 7.19) | 2.95 (2.06 to 4.11) | -2.19 (-2.83 to -1.55) |
| Belarus | 7.67 (6.57 to 8.81) | 6.59 (4.53 to 9.41) | -0.35 (-1.94 to 1.27) |
| Belgium | 4.67 (3.99 to 5.41) | 1.6 (1.26 to 1.94) | -3.66 (-4.24 to -3.08) |
| Belize | 8.57 (6.59 to 10.78) | 4.96 (3.61 to 6.55) | -2.32 (-3.83 to -0.79) |
| Benin | 6.24 (3.64 to 9.9) | 6.14 (3.42 to 9.98) | -0.06 (-0.21 to 0.1) |
| Bermuda | 6.6 (5.21 to 8.27) | 1.7 (1.19 to 2.32) | -4.58 (-4.92 to -4.24) |
| Bhutan | 18.47 (9.31 to 30.57) | 12.07 (6.67 to 19.28) | -1.48 (-1.61 to -1.35) |
| Bolivia (Plurinational State of) | 10.53 (5.12 to 18.74) | 5.67 (2.7 to 9.99) | -2.13 (-2.26 to -2) |
| Bosnia and Herzegovina | 7.89 (6.4 to 9.57) | 4.03 (2.68 to 5.91) | -2.26 (-2.76 to -1.75) |
| Botswana | 6.52 (3.35 to 11.48) | 8.1 (3.84 to 14.63) | 0.76 (0.15 to 1.38) |
| Brazil | 12.29 (11.56 to 13.03) | 6.02 (5.5 to 6.57) | -2.4 (-2.64 to -2.16) |
| Brunei Darussalam | 11.2 (7.33 to 15.94) | 4.87 (3.29 to 6.97) | -2.85 (-3.14 to -2.55) |
| Bulgaria | 10.36 (8.92 to 12.03) | 8.53 (5.95 to 11.77) | -0.59 (-1.34 to 0.17) |
| Burkina Faso | 7.64 (4.38 to 12.18) | 7.21 (4.07 to 11.59) | -0.16 (-0.62 to 0.3) |
| Burundi | 9.89 (4.76 to 17.79) | 8.13 (4.4 to 13.82) | -0.68 (-1.03 to -0.32) |
| Cabo Verde | 4.73 (3 to 7.01) | 4.24 (2.53 to 6.45) | -0.4 (-0.75 to -0.04) |
| Cambodia | 12.33 (6.92 to 20.57) | 7.49 (4.21 to 12.18) | -1.68 (-1.86 to -1.5) |
| Cameroon | 5.58 (2.93 to 9.62) | 6.14 (3.14 to 11) | 0.35 (0.15 to 0.55) |
| Canada | 3.46 (3 to 3.94) | 1.73 (1.41 to 2.1) | -2.26 (-2.64 to -1.89) |
| Central African Republic | 9.98 (5.33 to 17.14) | 10.96 (5.83 to 19.37) | 0.33 (0.01 to 0.64) |
| Chad | 6.3 (3.47 to 10.43) | 7.03 (3.85 to 11.63) | 0.47 (0.37 to 0.57) |
| Chile | 3.03 (2.53 to 3.57) | 1.52 (1.2 to 1.89) | -2.25 (-2.8 to -1.7) |
| China | 8.52 (6.64 to 10.82) | 4.48 (3.43 to 5.7) | -2.17 (-2.96 to -1.36) |
| Colombia | 10.26 (9.1 to 11.49) | 3.94 (2.73 to 5.5) | -3.08 (-3.42 to -2.74) |
| Comoros | 6.92 (1.87 to 12.86) | 7.91 (3.85 to 13.49) | 0.36 (-1.15 to 1.9) |
| Congo | 11.88 (5.58 to 20.39) | 10.41 (5.27 to 17.87) | -0.49 (-1 to 0.03) |
| Cook Islands | 10.39 (5.7 to 17.58) | 6.13 (2.89 to 9.77) | -1.79 (-2.01 to -1.57) |
| Costa Rica | 6.37 (5.36 to 7.47) | 2.94 (2.04 to 4.12) | -2.54 (-3.09 to -1.99) |
| Coted'Ivoire | 7.58 (4.36 to 11.92) | 6.95 (3.79 to 11.51) | -0.31 (-0.52 to -0.09) |
| Croatia | 6.29 (5.27 to 7.42) | 2.11 (1.44 to 2.97) | -4.09 (-5.02 to -3.16) |
| Cuba | 11.64 (10.38 to 12.99) | 3.85 (2.87 to 5.18) | -3.66 (-4.15 to -3.17) |
| Cyprus | 6.85 (4.86 to 9.3) | 2.26 (1.63 to 3.02) | -3.78 (-4.76 to -2.79) |
| Czechia | 7.01 (6.12 to 7.98) | 1.89 (1.36 to 2.59) | -4.74 (-5.14 to -4.34) |
| Democratic People's Republic of Korea | 9.76 (5 to 16.62) | 10.18 (5.68 to 16.78) | 0.15 (0.05 to 0.25) |
| Democratic Republic of the Congo | 7.8 (3.94 to 14.23) | 6.8 (3.38 to 12.11) | -0.51 (-0.79 to -0.24) |
| Denmark | 3.71 (3.17 to 4.3) | 0.82 (0.61 to 1.08) | -4.97 (-5.87 to -4.06) |
| Djibouti | 4.9 (2.55 to 8.33) | 6.26 (2.88 to 12.03) | 0.83 (0.64 to 1.03) |
| Dominica | 8.58 (5.79 to 12.01) | 4.57 (2.72 to 7.11) | -2.28 (-2.67 to -1.89) |
| Dominican Republic | 15.29 (11.69 to 19.25) | 17.12 (10.51 to 25.37) | 0.48 (-0.11 to 1.07) |
| Ecuador | 9.01 (7.28 to 11) | 5.19 (3.52 to 7.56) | -2.16 (-3.14 to -1.18) |
| Egypt | 38.78 (29.52 to 49.71) | 24.45 (14.94 to 35.78) | -1.55 (-1.8 to -1.3) |
| El Salvador | 14.48 (11.43 to 17.86) | 6.38 (4.04 to 9.69) | -2.96 (-3.58 to -2.33) |
| Equatorial Guinea | 9.15 (4.43 to 16.55) | 4.29 (1.77 to 8.86) | -2.48 (-2.93 to -2.04) |
| Eritrea | 5.68 (2.59 to 11.71) | 7.17 (3.73 to 12.23) | 0.82 (0.59 to 1.05) |
| Estonia | 6.47 (5.34 to 7.79) | 1.67 (1.09 to 2.43) | -4.33 (-6.22 to -2.4) |
| Eswatini | 6.17 (3.43 to 10.24) | 6.34 (2.77 to 12.23) | -0.04 (-0.78 to 0.7) |
| Ethiopia | 12.38 (7.88 to 20.7) | 4.98 (3.19 to 7.24) | -3.09 (-3.37 to -2.82) |
| Fiji | 21.26 (13.78 to 30.82) | 17.03 (10.44 to 25.13) | -0.78 (-1.09 to -0.47) |
| Finland | 3.38 (2.88 to 3.96) | 1.05 (0.79 to 1.38) | -3.89 (-4.79 to -2.98) |
| France | 1.84 (1.59 to 2.12) | 1.14 (0.89 to 1.42) | -1.63 (-1.99 to -1.27) |
| Gabon | 6.27 (3.25 to 10.53) | 4.84 (2.47 to 8.66) | -0.91 (-1.29 to -0.53) |
| Gambia | 6.18 (3.36 to 10.26) | 8.84 (4.99 to 14.37) | 1.36 (0.31 to 2.42) |
| Georgia | 20.2 (17.51 to 23.16) | 5.53 (4.14 to 7.21) | -4.44 (-5.38 to -3.48) |
| Germany | 4.87 (4.23 to 5.56) | 1.7 (1.37 to 2.07) | -3.64 (-3.87 to -3.4) |
| Ghana | 12.8 (7.42 to 19.87) | 8.97 (5.18 to 14.35) | -1.23 (-1.32 to -1.13) |
| Greece | 4.17 (3.63 to 4.77) | 3.88 (3.35 to 4.44) | -0.2 (-0.72 to 0.33) |
| Greenland | 9.79 (6.37 to 14.27) | 3.64 (2.31 to 5.4) | -3.41 (-3.84 to -2.98) |
| Grenada | 10 (8.03 to 12.21) | 4.51 (3.44 to 5.82) | -2.7 (-3.17 to -2.24) |
| Guam | 9.19 (6.26 to 12.76) | 16.42 (11.58 to 22.17) | 2.21 (1.91 to 2.51) |
| Guatemala | 21.49 (17.43 to 26.16) | 7.58 (5.48 to 10.15) | -3.65 (-5.32 to -1.95) |
| Guinea | 7.54 (4.16 to 12.38) | 8.87 (5.08 to 14.61) | 0.6 (0.42 to 0.78) |
| Guinea-Bissau | 13.18 (7.18 to 22.62) | 14.13 (7.87 to 22.52) | 0.25 (0.08 to 0.43) |
| Guyana | 21.18 (16.26 to 26.76) | 13.53 (8.94 to 19.29) | -1.53 (-2.38 to -0.68) |
| Haiti | 27.43 (16.06 to 43.45) | 18.55 (8.27 to 33.2) | -1.26 (-1.45 to -1.08) |
| Honduras | 14.62 (8.79 to 21.72) | 8.13 (3.94 to 13.88) | -1.94 (-2.48 to -1.4) |
| Hungary | 12.91 (11.63 to 14.29) | 4.42 (3.32 to 5.71) | -3.66 (-4.48 to -2.83) |
| Iceland | 3.92 (3.12 to 4.82) | 1.24 (0.97 to 1.54) | -3.92 (-4.06 to -3.77) |
| India | 18.54 (15.22 to 22.32) | 15.34 (11.67 to 19.39) | -0.65 (-1.07 to -0.22) |
| Indonesia | 17.06 (13.46 to 21.81) | 13.96 (10.55 to 18.65) | -0.69 (-0.89 to -0.49) |
| Iran (Islamic Republic of) | 20.17 (17.82 to 23.23) | 7.51 (6.8 to 8.26) | -3.38 (-3.52 to -3.24) |
| Iraq | 25.89 (17.58 to 36.21) | 14.03 (8.93 to 21.15) | -2.11 (-2.24 to -1.97) |
| Ireland | 4.56 (3.87 to 5.3) | 1.33 (1.08 to 1.59) | -4.11 (-5.11 to -3.1) |
| Israel | 4.48 (3.87 to 5.12) | 0.72 (0.57 to 0.88) | -6.24 (-6.58 to -5.9) |
| Italy | 2.73 (2.58 to 2.88) | 1.14 (1.05 to 1.24) | -3.03 (-3.19 to -2.86) |
| Jamaica | 5.27 (4.26 to 6.34) | 5.53 (3.86 to 7.66) | 0.44 (-0.98 to 1.87) |
| Japan | 1.9 (1.8 to 2) | 1.18 (1.08 to 1.26) | -1.64 (-2.01 to -1.27) |
| Jordan | 12.5 (8.14 to 17.69) | 4.03 (2.64 to 5.81) | -3.82 (-4.38 to -3.25) |
| Kazakhstan | 10.99 (9.48 to 12.55) | 5.9 (4.35 to 8.07) | -2.05 (-3.16 to -0.93) |
| Kenya | 2.61 (1.8 to 3.63) | 4.55 (3.03 to 6.5) | 1.92 (1.66 to 2.19) |
| Kiribati | 41.35 (25.99 to 61.32) | 35.61 (21.93 to 54.36) | -0.53 (-0.62 to -0.44) |
| Kuwait | 11.7 (9.87 to 13.65) | 3.24 (2.37 to 4.29) | -4.17 (-5.26 to -3.07) |
| Kyrgyzstan | 10.68 (9.1 to 12.42) | 6.5 (5.09 to 8.18) | -1.91 (-2.75 to -1.06) |
| Lao People's Democratic Republic | 16.71 (9.54 to 27.39) | 12.72 (7.5 to 19.96) | -0.93 (-1.03 to -0.82) |
| Latvia | 9.72 (8.2 to 11.5) | 3.24 (2.1 to 4.93) | -3.54 (-5.27 to -1.78) |
| Lebanon | 22.95 (15.55 to 31.53) | 12.35 (6.88 to 17.78) | -2.08 (-2.26 to -1.91) |
| Lesotho | 3.36 (1.72 to 5.82) | 7.9 (3.59 to 14.56) | 2.84 (2.27 to 3.41) |
| Liberia | 8.87 (5.09 to 14.14) | 8.47 (4.55 to 14.2) | -0.19 (-0.33 to -0.06) |
| Libya | 21.73 (14.47 to 30.8) | 18.45 (11.9 to 26.78) | -0.53 (-1.49 to 0.44) |
| Lithuania | 8.62 (7.38 to 9.92) | 3.76 (2.73 to 5.07) | -2.79 (-4.43 to -1.11) |
| Luxembourg | 3.74 (3.05 to 4.47) | 1.01 (0.78 to 1.27) | -4.45 (-4.8 to -4.09) |
| Madagascar | 8.71 (5.43 to 13.41) | 8.76 (4.59 to 14.6) | 0.01 (-0.38 to 0.4) |
| Malawi | 8.87 (4.97 to 14.13) | 5.03 (2.63 to 8.45) | -1.9 (-2.12 to -1.68) |
| Malaysia | 6.44 (4.25 to 9.02) | 5.59 (3.61 to 8.17) | -0.35 (-0.7 to 0.01) |
| Maldives | 19.78 (12.36 to 30.81) | 3.29 (2 to 4.87) | -6.01 (-6.26 to -5.75) |
| Mali | 11.42 (6.65 to 18.41) | 8.92 (4.86 to 14.69) | -0.85 (-1.11 to -0.58) |
| Malta | 5.15 (4.26 to 6.14) | 2.2 (1.75 to 2.68) | -2.86 (-3.1 to -2.63) |
| Marshall Islands | 24.29 (13.9 to 39.59) | 34.6 (18.06 to 59.42) | 1.21 (1.11 to 1.31) |
| Mauritania | 10.2 (6.02 to 16.21) | 6.36 (3.41 to 10.51) | -1.62 (-1.75 to -1.48) |
| Mauritius | 15.95 (13.59 to 18.47) | 5.52 (4 to 7.34) | -3.76 (-4.49 to -3.02) |
| Mexico | 5.92 (5.66 to 6.18) | 4.78 (3.75 to 6.03) | -0.5 (-0.8 to -0.21) |
| Micronesia (Federated States of) | 29.28 (15.13 to 49.15) | 28.81 (3.59 to 54.3) | -0.04 (-0.11 to 0.03) |
| Monaco | 2.93 (1.78 to 4.52) | 1.56 (0.84 to 2.53) | -2.13 (-2.25 to -2.02) |
| Mongolia | 34.22 (23.29 to 47.35) | 16.03 (10.13 to 24.04) | -2.57 (-3.2 to -1.93) |
| Montenegro | 4.95 (3.32 to 7.01) | 3.67 (2.38 to 5.36) | -0.98 (-1.46 to -0.51) |
| Morocco | 26.53 (17.87 to 37.31) | 19.24 (11.5 to 30.32) | -1.06 (-1.24 to -0.87) |
| Mozambique | 5.42 (2.98 to 8.74) | 5.37 (2.65 to 9.48) | -0.06 (-0.36 to 0.24) |
| Myanmar | 28.63 (15.81 to 47.03) | 7.7 (4.57 to 11.53) | -4.5 (-4.74 to -4.27) |
| Namibia | 5.73 (2.9 to 9.42) | 3.99 (1.99 to 7.16) | -1.16 (-1.61 to -0.71) |
| Nauru | 35.96 (21.61 to 56.48) | 44.3 (25.54 to 70.39) | 0.7 (0.54 to 0.86) |
| Nepal | 17.35 (9.75 to 27.78) | 9.74 (5.32 to 15.5) | -1.96 (-2.17 to -1.76) |
| Netherlands | 4.25 (3.69 to 4.83) | 1.45 (1.21 to 1.71) | -3.69 (-4.26 to -3.11) |
| New Zealand | 6.01 (5.27 to 6.85) | 1.94 (1.64 to 2.3) | -3.84 (-4.42 to -3.25) |
| Nicaragua | 7.95 (5.79 to 10.46) | 5.92 (4 to 8.22) | -0.93 (-1.39 to -0.46) |
| Niger | 7.8 (4.15 to 12.96) | 6.69 (3.53 to 11.27) | -0.52 (-0.76 to -0.27) |
| Nigeria | 7.03 (3.96 to 11.89) | 5 (3.02 to 8.03) | -1.18 (-1.49 to -0.88) |
| Niue | 16.71 (9.98 to 25.48) | 16.23 (8.56 to 26.32) | -0.14 (-0.35 to 0.07) |
| North Macedonia | 12.34 (9.64 to 15.4) | 6.87 (4.53 to 9.97) | -2.04 (-2.73 to -1.34) |
| Northern Mariana Islands | 10.63 (6.18 to 16.67) | 10.17 (6.38 to 14.92) | -0.13 (-0.38 to 0.12) |
| Norway | 3.91 (3.65 to 4.15) | 0.91 (0.81 to 1.03) | -4.95 (-5.4 to -4.5) |
| Oman | 23.06 (14.53 to 34.82) | 9.86 (6.87 to 13.74) | -2.81 (-3.14 to -2.48) |
| Pakistan | 22.78 (16.71 to 30.65) | 26.78 (17.58 to 38.95) | 0.58 (0.48 to 0.7) |
| Palau | 20.56 (11.46 to 34.39) | 18.58 (11.32 to 28.32) | -0.33 (-0.43 to -0.22) |
| Palestine | 14.8 (9.63 to 21.97) | 7.61 (5.49 to 10.22) | -2.27 (-2.56 to -1.98) |
| Panama | 5.83 (4.82 to 7.02) | 2.72 (1.82 to 3.83) | -2.67 (-4.06 to -1.26) |
| Papua New Guinea | 12.66 (5.5 to 24.51) | 16.36 (8.06 to 28.99) | 0.86 (0.68 to 1.04) |
| Paraguay | 9.33 (6.99 to 12.03) | 5.28 (3.3 to 8.05) | -1.92 (-2.98 to -0.85) |
| Peru | 7.99 (5.57 to 10.99) | 2.89 (1.59 to 4.66) | -3.68 (-4.91 to -2.43) |
| Philippines | 4.29 (3.5 to 5.25) | 13.71 (9.51 to 18.59) | 4.25 (3.76 to 4.75) |
| Poland | 9.35 (8.96 to 9.73) | 1.97 (1.49 to 2.59) | -5.46 (-6.29 to -4.63) |
| Portugal | 4.07 (3.49 to 4.7) | 1.28 (1.04 to 1.54) | -3.97 (-4.61 to -3.34) |
| Puerto Rico | 6.88 (5.96 to 7.87) | 2.84 (1.99 to 3.91) | -3.58 (-3.79 to -3.36) |
| Qatar | 15.32 (10.15 to 21.83) | 3.92 (2.45 to 5.87) | -4.59 (-6.2 to -2.96) |
| Republic of Korea | 8.92 (7.13 to 10.81) | 0.7 (0.48 to 0.98) | -8.52 (-8.82 to -8.21) |
| Republic of Moldova | 9.22 (8.02 to 10.46) | 5.37 (4.23 to 6.65) | -1.58 (-3.05 to -0.09) |
| Romania | 8.84 (7.78 to 10.02) | 4.91 (3.7 to 6.36) | -1.98 (-2.66 to -1.3) |
| Russian Federation | 8.81 (7.55 to 9.42) | 7.67 (5.68 to 10.2) | -0.35 (-1.56 to 0.87) |
| Rwanda | 7.09 (3.39 to 13.22) | 3.8 (1.76 to 7.26) | -2.18 (-2.48 to -1.88) |
| Saint Kitts and Nevis | 15.76 (12.34 to 19.61) | 2.89 (0.96 to 4.74) | -5.81 (-6.02 to -5.61) |
| Saint Lucia | 8.21 (6.32 to 10.38) | 2.91 (2.15 to 3.84) | -3.7 (-4.08 to -3.32) |
| Saint Vincent and the Grenadines | 13.56 (10.81 to 16.67) | 7.67 (5.58 to 10.3) | -1.97 (-2.29 to -1.65) |
| Samoa | 18.06 (10.78 to 27.48) | 23.34 (12.31 to 36.96) | 0.89 (0.84 to 0.94) |
| San Marino | 1.29 (0.76 to 2.05) | 1.03 (0.5 to 1.84) | -0.77 (-0.92 to -0.63) |
| Sao Tome and Principe | 7.2 (4.07 to 11.12) | 8.73 (4.86 to 13.9) | 0.75 (0.41 to 1.09) |
| Saudi Arabia | 21.13 (13.02 to 31.86) | 19.28 (12.67 to 27.61) | -0.31 (-0.47 to -0.16) |
| Senegal | 7.74 (4.54 to 12.28) | 6.45 (3.45 to 10.61) | -0.53 (-1.02 to -0.03) |
| Serbia | 8.04 (5.71 to 10.82) | 3.94 (2.62 to 5.73) | -2.35 (-2.97 to -1.72) |
| Seychelles | 9.65 (6.87 to 12.96) | 5.75 (3.86 to 8.12) | -1.76 (-2.1 to -1.41) |
| Sierra Leone | 8.56 (4.23 to 14.55) | 10.35 (5.37 to 17.59) | 0.65 (0.54 to 0.77) |
| Singapore | 3.71 (3.05 to 4.43) | 1.16 (0.92 to 1.43) | -3.8 (-4.53 to -3.06) |
| Slovakia | 9.4 (7.5 to 11.61) | 3.26 (2.1 to 4.79) | -3.69 (-4.66 to -2.71) |
| Slovenia | 3.28 (2.2 to 4.64) | 0.81 (0.55 to 1.16) | -5.12 (-5.78 to -4.46) |
| Solomon Islands | 79.44 (45.78 to 128.12) | 77.77 (47.8 to 121.19) | -0.1 (-0.23 to 0.03) |
| Somalia | 7.71 (3.87 to 14.24) | 9.72 (5.05 to 16.64) | 0.82 (0.72 to 0.92) |
| South Africa | 9.82 (8 to 11.84) | 3.86 (2.27 to 5.64) | -3.43 (-4.56 to -2.28) |
| South Sudan | 3.86 (1.9 to 6.81) | 4.39 (2.08 to 8.08) | 0.39 (0.08 to 0.7) |
| Spain | 2.76 (2.36 to 3.19) | 1.23 (0.99 to 1.5) | -2.76 (-3.27 to -2.25) |
| Sri Lanka | 10.11 (7.26 to 13.6) | 4.67 (2.74 to 7.19) | -2.45 (-3.07 to -1.83) |
| Sudan | 40.15 (24.85 to 60.06) | 22.36 (11.52 to 37.87) | -2 (-2.12 to -1.89) |
| Suriname | 11.5 (8.04 to 15.29) | 7.46 (4.74 to 11.07) | -1.32 (-2.69 to 0.06) |
| Sweden | 3.14 (2.72 to 3.6) | 0.98 (0.77 to 1.22) | -3.93 (-4.69 to -3.18) |
| Switzerland | 2.59 (2.14 to 3.09) | 0.79 (0.6 to 1.01) | -4.02 (-4.98 to -3.05) |
| Syrian Arab Republic | 47.48 (33.91 to 63.67) | 27.28 (18.9 to 38.9) | -1.98 (-2.41 to -1.55) |
| Taiwan (Province of China) | 2.89 (2.43 to 3.4) | 1.37 (0.96 to 1.88) | -2.46 (-2.9 to -2.01) |
| Tajikistan | 15.87 (12.85 to 19.2) | 14.43 (9.93 to 20.38) | -0.11 (-0.48 to 0.27) |
| Thailand | 7.13 (4.64 to 10.42) | 3.01 (1.7 to 4.81) | -2.81 (-3.27 to -2.35) |
| Timor-Leste | 7.34 (3.94 to 12.31) | 9.31 (2.51 to 15.4) | 0.89 (0.3 to 1.49) |
| Togo | 8.68 (4.98 to 13.42) | 7.71 (4.07 to 12.87) | -0.41 (-0.81 to -0.01) |
| Tokelau | 26.31 (14.46 to 43.2) | 26.63 (15.31 to 41.65) | 0.04 (-0.02 to 0.1) |
| Tonga | 8.05 (5.03 to 12.33) | 7.08 (4 to 11.54) | -0.45 (-0.54 to -0.37) |
| Trinidad and Tobago | 15.59 (13.36 to 17.95) | 6.6 (4.29 to 9.55) | -3.18 (-4.02 to -2.33) |
| Tunisia | 13.84 (9.42 to 19.28) | 8.74 (5.05 to 13.26) | -1.62 (-1.78 to -1.46) |
| Turkey | 11.8 (8.15 to 16.78) | 3.91 (2.67 to 5.42) | -3.8 (-4.35 to -3.25) |
| Turkmenistan | 18.46 (15.96 to 21.09) | 15.82 (11.48 to 21.33) | -0.27 (-1.09 to 0.55) |
| Tuvalu | 28.36 (16.03 to 45.7) | 27.99 (16.4 to 44.2) | -0.04 (-0.13 to 0.04) |
| Uganda | 2.54 (1.04 to 4.9) | 3.87 (1.87 to 6.96) | 1.43 (1 to 1.86) |
| Ukraine | 7.29 (5.91 to 8.57) | 10.81 (7.6 to 14.83) | 2.06 (-0.29 to 4.47) |
| United Arab Emirates | 7.22 (4.08 to 11.69) | 5.14 (2.85 to 8.37) | -1.2 (-1.66 to -0.73) |
| United Kingdom | 4.44 (4.3 to 4.63) | 1.99 (1.88 to 2.17) | -2.66 (-2.83 to -2.49) |
| United Republic of Tanzania | 3.27 (1.8 to 5.5) | 4.12 (2.22 to 7.04) | 0.81 (0.5 to 1.13) |
| United States of America | 5.95 (5.7 to 6.18) | 4.64 (4.36 to 5.08) | -0.83 (-1.11 to -0.55) |
| United States Virgin Islands | 10.53 (7.15 to 14.74) | 5.18 (3.18 to 8.03) | -2.4 (-2.62 to -2.18) |
| Uruguay | 5.3 (4.41 to 6.27) | 1.89 (1.51 to 2.31) | -3.46 (-4.22 to -2.7) |
| Uzbekistan | 12.46 (10.71 to 14.16) | 21.27 (16.69 to 26.59) | 1.94 (1.41 to 2.48) |
| Vanuatu | 20.38 (10.03 to 34.57) | 29.04 (15.14 to 47.53) | 1.18 (0.87 to 1.49) |
| Venezuela (Bolivarian Republic of) | 12.49 (11.18 to 13.91) | 8.55 (5.91 to 12) | -1.07 (-1.8 to -0.34) |
| Viet Nam | 6.97 (4.14 to 10.82) | 3.7 (2.14 to 5.83) | -2.17 (-2.25 to -2.08) |
| Yemen | 34.7 (20.79 to 53.67) | 25.14 (14.54 to 40.31) | -1.09 (-1.23 to -0.96) |
| Zambia | 10.89 (5.95 to 18.32) | 7.4 (4 to 12.5) | -1.34 (-1.66 to -1.02) |
| Zimbabwe | 4.17 (2.48 to 6.5) | 7.82 (4.2 to 13.23) | 2.23 (1.32 to 3.15) |

**Table S6.** AAPCs of 204 countries in ASDR of IHD in WCBA. Abbreviations: ASPR, age-standardized prevalence rate; IHD, ischemic heart disease; WCBA, women of childbearing age; AAPC, average annual percent change; CI, confidence interval.

| In ASDR | AAPC (95% CI) |  | AAPC (95% CI) |  | AAPC (95% CI) |  |
| --- | --- | --- | --- | --- | --- | --- |
| location | 1990-1999 | p value | 2000-2009 | p value | 2010-2019 | p value |
| Afghanistan | 0.66 (0.52 to 0.81) | 0.000 | -2.35 (-2.45 to -2.25) | 0.000 | -1.86 (-2.11 to -1.62) | 0.000 |
| Albania | -1.69 (-2.56 to -0.81) | 0.000 | 0.1 (-0.2 to 0.41) | 0.513 | -2 (-2.41 to -1.58) | 0.000 |
| Algeria | -2.93 (-3.04 to -2.83) | 0.000 | -3.87 (-4.06 to -3.68) | 0.000 | -1.68 (-2.04 to -1.31) | 0.000 |
| American Samoa | 1.33 (1.07 to 1.6) | 0.000 | 1.08 (0.66 to 1.51) | 0.000 | 1.36 (1.08 to 1.63) | 0.000 |
| Andorra | -1.85 (-2.13 to -1.57) | 0.000 | -2.78 (-2.86 to -2.69) | 0.000 | -0.63 (-0.75 to -0.51) | 0.000 |
| Angola | 0.66 (0.36 to 0.95) | 0.000 | -1.59 (-1.87 to -1.31) | 0.000 | -1.84 (-2.76 to -0.91) | 0.000 |
| Antigua and Barbuda | -3.32 (-3.65 to -2.99) | 0.000 | -4.33 (-4.7 to -3.97) | 0.000 | -0.27 (-0.76 to 0.21) | 0.267 |
| Argentina | -4.3 (-4.43 to -4.17) | 0.000 | -2.5 (-2.94 to -2.05) | 0.000 | -1.77 (-2.64 to -0.91) | 0.000 |
| Armenia | -1.03 (-2.68 to 0.66) | 0.230 | -0.45 (-2.36 to 1.5) | 0.648 | -2.27 (-2.81 to -1.73) | 0.000 |
| Australia | -2.41 (-3.94 to -0.85) | 0.002 | -3.27 (-3.5 to -3.03) | 0.000 | -1.95 (-2.48 to -1.41) | 0.000 |
| Austria | -0.61 (-1.73 to 0.51) | 0.285 | -6.82 (-8.25 to -5.38) | 0.000 | -3.69 (-5.72 to -1.62) | 0.001 |
| Azerbaijan | 0.31 (-0.43 to 1.04) | 0.414 | -1.98 (-2.35 to -1.6) | 0.000 | -2.27 (-4.83 to 0.36) | 0.090 |
| Bahamas | -2.51 (-3.08 to -1.95) | 0.000 | -3.21 (-4.04 to -2.37) | 0.000 | -0.19 (-0.5 to 0.12) | 0.211 |
| Bahrain | -2.18 (-5.19 to 0.93) | 0.168 | -9.84 (-12.44 to -7.16) | 0.000 | -0.66 (-1.66 to 0.36) | 0.203 |
| Bangladesh | -5.15 (-5.88 to -4.42) | 0.000 | 0.56 (-0.21 to 1.33) | 0.156 | -1.22 (-1.61 to -0.82) | 0.000 |
| Barbados | -3.27 (-3.99 to -2.55) | 0.000 | -2.09 (-3.73 to -0.43) | 0.014 | -0.66 (-1.32 to 0) | 0.051 |
| Belarus | 4.27 (2.73 to 5.83) | 0.000 | -0.94 (-3.63 to 1.84) | 0.504 | -4.57 (-7.76 to -1.26) | 0.007 |
| Belgium | -3.3 (-4.6 to -1.98) | 0.000 | -4.47 (-4.7 to -4.25) | 0.000 | -3.01 (-4.29 to -1.72) | 0.000 |
| Belize | -0.05 (-4.21 to 4.29) | 0.981 | -3.04 (-3.39 to -2.68) | 0.000 | -3.04 (-3.39 to -2.68) | 0.000 |
| Benin | 1.25 (0.86 to 1.63) | 0.000 | -1.27 (-1.39 to -1.15) | 0.000 | -0.2 (-0.44 to 0.04) | 0.095 |
| Bermuda | -4.98 (-5.68 to -4.27) | 0.000 | -6.55 (-6.94 to -6.17) | 0.000 | -1.72 (-2.32 to -1.12) | 0.000 |
| Bhutan | -0.63 (-0.94 to -0.31) | 0.000 | -2.55 (-2.67 to -2.43) | 0.000 | -1.16 (-1.36 to -0.97) | 0.000 |
| Bolivia (Plurinational State of) | -2.47 (-2.8 to -2.13) | 0.000 | -2.36 (-2.46 to -2.26) | 0.000 | -1.4 (-1.49 to -1.31) | 0.000 |
| Bosnia and Herzegovina | -0.96 (-2.14 to 0.24) | 0.117 | -4.08 (-4.41 to -3.74) | 0.000 | -1.3 (-2.24 to -0.34) | 0.008 |
| Botswana | 0.64 (-0.68 to 1.98) | 0.344 | 1.12 (-0.17 to 2.43) | 0.090 | -0.44 (-0.8 to -0.09) | 0.014 |
| Brazil | -2.18 (-2.52 to -1.84) | 0.000 | -2.37 (-2.99 to -1.76) | 0.000 | -2.56 (-2.82 to -2.29) | 0.000 |
| Brunei Darussalam | -7.19 (-7.76 to -6.62) | 0.000 | -1.37 (-1.91 to -0.83) | 0.000 | 0.26 (0.06 to 0.47) | 0.010 |
| Bulgaria | 4.65 (3.15 to 6.17) | 0.000 | -4.61 (-5 to -4.21) | 0.000 | -1.03 (-2.26 to 0.21) | 0.103 |
| Burkina Faso | 0.04 (-0.76 to 0.85) | 0.917 | -1.07 (-1.61 to -0.53) | 0.000 | 0.79 (-0.19 to 1.79) | 0.116 |
| Burundi | 1.6 (0.85 to 2.36) | 0.000 | -2.39 (-3.07 to -1.71) | 0.000 | -1.01 (-1.49 to -0.54) | 0.000 |
| Cabo Verde | 3.43 (2.99 to 3.87) | 0.000 | -2.32 (-2.67 to -1.97) | 0.000 | -2.02 (-2.8 to -1.24) | 0.000 |
| Cambodia | -0.57 (-0.87 to -0.26) | 0.000 | -3.28 (-3.58 to -2.99) | 0.000 | -1.13 (-1.36 to -0.9) | 0.000 |
| Cameroon | 2.46 (2.19 to 2.72) | 0.000 | -0.21 (-0.69 to 0.28) | 0.406 | -1.46 (-1.73 to -1.18) | 0.000 |
| Canada | -2.36 (-2.47 to -2.26) | 0.000 | -2.36 (-2.47 to -2.26) | 0.000 | -1.77 (-2.64 to -0.9) | 0.000 |
| Central African Republic | 0.94 (0.25 to 1.63) | 0.008 | -0.09 (-0.37 to 0.2) | 0.521 | 0.04 (-0.5 to 0.59) | 0.873 |
| Chad | 1.44 (1.28 to 1.6) | 0.000 | 0.4 (0.3 to 0.5) | 0.000 | -0.43 (-0.57 to -0.28) | 0.000 |
| Chile | -3.79 (-5.38 to -2.16) | 0.000 | -1.65 (-1.9 to -1.4) | 0.000 | -1.34 (-1.62 to -1.06) | 0.000 |
| China | 0.73 (0.17 to 1.29) | 0.013 | -4.83 (-6.93 to -2.67) | 0.000 | -2.86 (-3.5 to -2.21) | 0.000 |
| Colombia | -4.7 (-5.38 to -4.01) | 0.000 | -3.57 (-3.84 to -3.3) | 0.000 | -0.58 (-1.31 to 0.15) | 0.121 |
| Comoros | 1.97 (0.81 to 3.14) | 0.002 | -1.82 (-2.66 to -0.96) | 0.000 | 1.1 (-3.42 to 5.82) | 0.640 |
| Congo | 1.73 (0.46 to 3.01) | 0.007 | -0.47 (-1.36 to 0.42) | 0.300 | -2.22 (-2.44 to -2) | 0.000 |
| Cook Islands | -3.76 (-4.05 to -3.47) | 0.000 | -1.64 (-2.17 to -1.11) | 0.000 | -0.12 (-0.39 to 0.16) | 0.403 |
| Costa Rica | -4.24 (-4.69 to -3.78) | 0.000 | -3.74 (-4.15 to -3.33) | 0.000 | 0.3 (-1.04 to 1.65) | 0.652 |
| Coted'Ivoire | 0.77 (0.22 to 1.31) | 0.006 | -0.13 (-0.29 to 0.04) | 0.130 | -1.8 (-2.05 to -1.55) | 0.000 |
| Croatia | -3.65 (-6.34 to -0.89) | 0.010 | -2.8 (-3.45 to -2.15) | 0.000 | -6.07 (-7.16 to -4.97) | 0.000 |
| Cuba | -4.41 (-5.14 to -3.67) | 0.000 | -5.03 (-5.56 to -4.49) | 0.000 | -1.06 (-2.06 to -0.05) | 0.041 |
| Cyprus | -1.02 (-1.7 to -0.34) | 0.006 | -5.59 (-7.95 to -3.18) | 0.000 | -3.54 (-4.65 to -2.41) | 0.000 |
| Czechia | -5.29 (-5.53 to -5.06) | 0.000 | -5.29 (-5.53 to -5.06) | 0.000 | -3.49 (-4.73 to -2.24) | 0.000 |
| Democratic People's Republic of Korea | 1 (0.82 to 1.19) | 0.000 | 0.43 (0.27 to 0.59) | 0.000 | -1.02 (-1.2 to -0.83) | 0.000 |
| Democratic Republic of the Congo | 0.14 (-0.23 to 0.5) | 0.466 | -0.74 (-1.12 to -0.36) | 0.000 | -1 (-1.48 to -0.51) | 0.000 |
| Denmark | -3.82 (-5.84 to -1.76) | 0.000 | -6.82 (-7.78 to -5.86) | 0.000 | -3.89 (-5.29 to -2.47) | 0.000 |
| Djibouti | 2.08 (1.82 to 2.33) | 0.000 | 0.93 (0.74 to 1.12) | 0.000 | -0.48 (-0.79 to -0.16) | 0.005 |
| Dominica | -3.04 (-3.52 to -2.56) | 0.000 | -2.99 (-3.84 to -2.13) | 0.000 | -1.11 (-1.56 to -0.66) | 0.000 |
| Dominican Republic | -0.4 (-0.96 to 0.18) | 0.174 | 1.42 (0.55 to 2.29) | 0.001 | 1.11 (-0.17 to 2.4) | 0.089 |
| Ecuador | -3.68 (-4.19 to -3.17) | 0.000 | -1.4 (-4.13 to 1.41) | 0.325 | -1.03 (-2.29 to 0.25) | 0.114 |
| Egypt | -2.88 (-3.22 to -2.53) | 0.000 | -1.45 (-1.71 to -1.2) | 0.000 | -0.3 (-0.69 to 0.09) | 0.122 |
| El Salvador | -3.89 (-5.6 to -2.15) | 0.000 | -2.35 (-2.72 to -1.98) | 0.000 | -2.35 (-2.72 to -1.98) | 0.000 |
| Equatorial Guinea | -2.14 (-2.84 to -1.44) | 0.000 | -3.62 (-4.08 to -3.15) | 0.000 | -1.35 (-2.23 to -0.46) | 0.003 |
| Eritrea | -0.15 (-0.81 to 0.51) | 0.654 | 2.07 (1.84 to 2.29) | 0.000 | 0.31 (0.2 to 0.42) | 0.000 |
| Estonia | 1.4 (-2.54 to 5.5) | 0.492 | -10.16 (-13.52 to -6.67) | 0.000 | -3.8 (-6.17 to -1.37) | 0.002 |
| Eswatini | 0.13 (-0.37 to 0.63) | 0.609 | 3.37 (1.22 to 5.57) | 0.002 | -4.37 (-4.82 to -3.93) | 0.000 |
| Ethiopia | -0.93 (-1.51 to -0.34) | 0.002 | -5.52 (-5.87 to -5.17) | 0.000 | -2.09 (-2.51 to -1.67) | 0.000 |
| Fiji | -0.59 (-0.83 to -0.35) | 0.000 | -0.44 (-1.12 to 0.25) | 0.212 | -0.97 (-1.33 to -0.61) | 0.000 |
| Finland | -2.74 (-3.67 to -1.8) | 0.000 | -5.5 (-5.92 to -5.09) | 0.000 | -3.59 (-6.24 to -0.86) | 0.010 |
| France | -0.53 (-0.86 to -0.19) | 0.002 | -1.37 (-1.7 to -1.04) | 0.000 | -3.23 (-4.18 to -2.27) | 0.000 |
| Gabon | 1.11 (0.47 to 1.76) | 0.001 | -1.16 (-1.8 to -0.52) | 0.000 | -2.58 (-3.16 to -2) | 0.000 |
| Gambia | 1.1 (0.51 to 1.69) | 0.001 | 1.59 (-1.41 to 4.68) | 0.303 | 1.74 (0.74 to 2.75) | 0.001 |
| Georgia | -0.79 (-2.98 to 1.45) | 0.487 | -5.84 (-6.92 to -4.74) | 0.000 | -5.68 (-7.1 to -4.23) | 0.000 |
| Germany | -4.27 (-4.63 to -3.9) | 0.000 | -4.32 (-4.55 to -4.1) | 0.000 | -2.22 (-2.74 to -1.71) | 0.000 |
| Ghana | -0.73 (-0.84 to -0.62) | 0.000 | -1 (-1.09 to -0.91) | 0.000 | -1.92 (-2.1 to -1.74) | 0.000 |
| Greece | 0.73 (0.55 to 0.9) | 0.000 | 0.73 (0.55 to 0.9) | 0.000 | -2.22 (-3.83 to -0.58) | 0.008 |
| Greenland | -1.94 (-2.22 to -1.65) | 0.000 | -4.56 (-5.64 to -3.47) | 0.000 | -3.23 (-3.59 to -2.86) | 0.000 |
| Grenada | -4.99 (-6.11 to -3.86) | 0.000 | -2.38 (-2.71 to -2.05) | 0.000 | -0.36 (-1.16 to 0.45) | 0.380 |
| Guam | 4.24 (3.32 to 5.17) | 0.000 | 1.31 (1.17 to 1.44) | 0.000 | 1.31 (1.17 to 1.44) | 0.000 |
| Guatemala | -4.53 (-9.26 to 0.45) | 0.074 | -4.45 (-6.12 to -2.76) | 0.000 | -1.43 (-1.89 to -0.97) | 0.000 |
| Guinea | -0.04 (-0.19 to 0.12) | 0.637 | 1.82 (1.34 to 2.3) | 0.000 | -0.08 (-0.3 to 0.14) | 0.495 |
| Guinea-Bissau | 1.07 (0.99 to 1.15) | 0.000 | 1.35 (0.97 to 1.73) | 0.000 | -1.5 (-1.89 to -1.11) | 0.000 |
| Guyana | -0.19 (-1.06 to 0.69) | 0.672 | -1.82 (-4.03 to 0.45) | 0.116 | -2.2 (-3.01 to -1.38) | 0.000 |
| Haiti | -3.03 (-3.49 to -2.58) | 0.000 | 0 (-0.29 to 0.29) | 0.998 | -0.99 (-1.12 to -0.86) | 0.000 |
| Honduras | -1.67 (-1.96 to -1.38) | 0.000 | -2.23 (-2.71 to -1.75) | 0.000 | -1.7 (-2.93 to -0.45) | 0.008 |
| Hungary | -1.82 (-2.99 to -0.65) | 0.002 | -4.54 (-4.87 to -4.22) | 0.000 | -4.23 (-6.46 to -1.95) | 0.000 |
| Iceland | -3.46 (-3.69 to -3.22) | 0.000 | -6.4 (-6.68 to -6.11) | 0.000 | -1.89 (-2.07 to -1.7) | 0.000 |
| India | 0.05 (-0.85 to 0.95) | 0.922 | -1.04 (-1.51 to -0.56) | 0.000 | -0.91 (-1.74 to -0.07) | 0.034 |
| Indonesia | -0.43 (-0.8 to -0.06) | 0.025 | -0.3 (-0.6 to 0.01) | 0.060 | -1.55 (-1.8 to -1.31) | 0.000 |
| Iran (Islamic Republic of) | -1.63 (-1.73 to -1.52) | 0.000 | -6.67 (-7.04 to -6.3) | 0.000 | -1.68 (-1.78 to -1.58) | 0.000 |
| Iraq | 0.82 (0.63 to 1.01) | 0.000 | -2.97 (-3.11 to -2.84) | 0.000 | -3.67 (-3.95 to -3.38) | 0.000 |
| Ireland | -2.63 (-3.24 to -2.03) | 0.000 | -4.44 (-7 to -1.81) | 0.001 | -4.98 (-6.57 to -3.37) | 0.000 |
| Israel | -8.65 (-9.08 to -8.21) | 0.000 | -6.62 (-7.03 to -6.2) | 0.000 | -3.06 (-3.91 to -2.2) | 0.000 |
| Italy | -3.4 (-3.47 to -3.33) | 0.000 | -3.4 (-3.47 to -3.33) | 0.000 | -2.19 (-2.7 to -1.67) | 0.000 |
| Jamaica | 0.27 (-0.64 to 1.18) | 0.541 | -1.28 (-3.1 to 0.57) | 0.173 | 2.06 (-1.89 to 6.18) | 0.311 |
| Japan | -1.03 (-1.69 to -0.37) | 0.002 | -1.11 (-1.32 to -0.9) | 0.000 | -2.89 (-3.83 to -1.93) | 0.000 |
| Jordan | -0.63 (-1.21 to -0.06) | 0.031 | -8.2 (-9.2 to -7.19) | 0.000 | -2.19 (-2.82 to -1.55) | 0.000 |
| Kazakhstan | 7.56 (6.99 to 8.13) | 0.000 | -6.29 (-8.77 to -3.74) | 0.000 | -6.1 (-8.3 to -3.86) | 0.000 |
| Kenya | 3.66 (3.03 to 4.3) | 0.000 | 3.03 (2.48 to 3.58) | 0.000 | -1.04 (-1.15 to -0.94) | 0.000 |
| Kiribati | -0.43 (-0.69 to -0.17) | 0.001 | -0.44 (-0.54 to -0.33) | 0.000 | -0.76 (-0.82 to -0.7) | 0.000 |
| Kuwait | -1.39 (-2.75 to -0.02) | 0.047 | -4.87 (-5.34 to -4.4) | 0.000 | -6.02 (-9.1 to -2.85) | 0.000 |
| Kyrgyzstan | 0.56 (-2.06 to 3.25) | 0.678 | -1.64 (-2.23 to -1.04) | 0.000 | -4.49 (-4.83 to -4.15) | 0.000 |
| Lao People's Democratic Republic | 0.22 (0.13 to 0.31) | 0.000 | -1.03 (-1.22 to -0.84) | 0.000 | -1.96 (-2.1 to -1.82) | 0.000 |
| Latvia | -1.4 (-4.4 to 1.7) | 0.371 | -4.28 (-6.56 to -1.95) | 0.000 | -4.17 (-7.12 to -1.13) | 0.007 |
| Lebanon | -4.04 (-4.26 to -3.81) | 0.000 | -1.98 (-2.46 to -1.5) | 0.000 | -0.01 (-0.16 to 0.13) | 0.850 |
| Lesotho | -0.47 (-1.31 to 0.38) | 0.281 | 8.57 (7.81 to 9.33) | 0.000 | -0.69 (-1.71 to 0.33) | 0.186 |
| Liberia | 0 (-0.25 to 0.25) | 0.989 | 0.25 (0.09 to 0.4) | 0.002 | -0.69 (-0.9 to -0.48) | 0.000 |
| Libya | -1.12 (-1.76 to -0.49) | 0.002 | 0.3 (-1.53 to 2.16) | 0.753 | -0.32 (-2.04 to 1.44) | 0.721 |
| Lithuania | -3.56 (-5.43 to -1.65) | 0.000 | -0.29 (-4.37 to 3.97) | 0.893 | -3.78 (-5.02 to -2.51) | 0.000 |
| Luxembourg | -3.16 (-4.13 to -2.19) | 0.000 | -5.71 (-5.92 to -5.5) | 0.000 | -4.16 (-4.67 to -3.65) | 0.000 |
| Madagascar | 1.31 (0.14 to 2.5) | 0.028 | -0.38 (-0.59 to -0.17) | 0.000 | -0.88 (-1.03 to -0.72) | 0.000 |
| Malawi | -0.06 (-0.34 to 0.22) | 0.646 | -4.03 (-4.37 to -3.69) | 0.000 | -1.18 (-1.61 to -0.74) | 0.000 |
| Malaysia | -0.9 (-1.27 to -0.53) | 0.000 | 0.55 (-0.07 to 1.17) | 0.083 | -0.85 (-1.59 to -0.11) | 0.027 |
| Maldives | -5.69 (-5.97 to -5.4) | 0.000 | -8.8 (-9.46 to -8.13) | 0.000 | -3.44 (-3.7 to -3.18) | 0.000 |
| Mali | -0.42 (-0.94 to 0.09) | 0.108 | -1.49 (-1.66 to -1.32) | 0.000 | -0.5 (-1.12 to 0.12) | 0.117 |
| Malta | -3.35 (-3.91 to -2.79) | 0.000 | -2.94 (-3.23 to -2.64) | 0.000 | -2.27 (-2.61 to -1.94) | 0.000 |
| Marshall Islands | 3.75 (3.55 to 3.95) | 0.000 | 1.1 (0.84 to 1.36) | 0.000 | -1.02 (-1.07 to -0.96) | 0.000 |
| Mauritania | -1.72 (-1.96 to -1.48) | 0.000 | -1.37 (-1.49 to -1.25) | 0.000 | -1.81 (-2.11 to -1.51) | 0.000 |
| Mauritius | -1.8 (-2.74 to -0.85) | 0.001 | -7.4 (-8.42 to -6.37) | 0.000 | -1.76 (-3.56 to 0.08) | 0.060 |
| Mexico | -1.7 (-2.01 to -1.39) | 0.000 | -0.81 (-1.08 to -0.54) | 0.000 | 0.99 (0.4 to 1.59) | 0.002 |
| Micronesia (Federated States of) | 0.15 (0.01 to 0.3) | 0.041 | 0.55 (0.49 to 0.6) | 0.000 | -0.89 (-1.06 to -0.72) | 0.000 |
| Monaco | -3.51 (-3.79 to -3.23) | 0.000 | -1.06 (-1.17 to -0.95) | 0.000 | -1.65 (-1.77 to -1.53) | 0.000 |
| Mongolia | 1.73 (0.75 to 2.72) | 0.001 | -5.77 (-6.14 to -5.4) | 0.000 | -3.08 (-4.67 to -1.47) | 0.000 |
| Montenegro | 3.2 (1.96 to 4.45) | 0.000 | -3.65 (-4.26 to -3.03) | 0.000 | -1.83 (-2.33 to -1.32) | 0.000 |
| Morocco | 0.15 (-0.34 to 0.64) | 0.555 | -0.91 (-1.06 to -0.77) | 0.000 | -2.15 (-2.25 to -2.05) | 0.000 |
| Mozambique | -2.44 (-2.58 to -2.3) | 0.000 | 1.87 (1.22 to 2.52) | 0.000 | 0.18 (-0.43 to 0.79) | 0.558 |
| Myanmar | -1.67 (-2.17 to -1.17) | 0.000 | -6.33 (-6.66 to -6) | 0.000 | -5.04 (-5.46 to -4.62) | 0.000 |
| Namibia | 1.39 (0.79 to 1.99) | 0.000 | -4.43 (-5.5 to -3.35) | 0.000 | -0.78 (-1.04 to -0.53) | 0.000 |
| Nauru | 3.68 (3.38 to 3.99) | 0.000 | 0.96 (0.75 to 1.17) | 0.000 | -2.35 (-2.65 to -2.05) | 0.000 |
| Nepal | -3.08 (-3.36 to -2.8) | 0.000 | -1.69 (-2.16 to -1.22) | 0.000 | -0.97 (-1.3 to -0.63) | 0.000 |
| Netherlands | 0.46 (0.12 to 0.8) | 0.011 | -7.98 (-9.46 to -6.47) | 0.000 | -3.4 (-4.22 to -2.59) | 0.000 |
| New Zealand | -4.85 (-6.34 to -3.34) | 0.000 | -3.96 (-4.15 to -3.77) | 0.000 | -2.66 (-3.72 to -1.59) | 0.000 |
| Nicaragua | -4.18 (-5.32 to -3.02) | 0.000 | 0.83 (0.42 to 1.24) | 0.000 | 0.83 (0.42 to 1.24) | 0.000 |
| Niger | 0.11 (-0.1 to 0.33) | 0.289 | -2.17 (-2.33 to -2.02) | 0.000 | 0.78 (0.05 to 1.52) | 0.036 |
| Nigeria | 1.49 (0.95 to 2.03) | 0.000 | -3.99 (-4.52 to -3.47) | 0.000 | -1 (-1.32 to -0.67) | 0.000 |
| Niue | 0.93 (0.46 to 1.4) | 0.000 | 0.06 (-0.33 to 0.46) | 0.754 | -1.2 (-1.4 to -0.99) | 0.000 |
| North Macedonia | 1.29 (0.15 to 2.45) | 0.026 | -4.7 (-5.97 to -3.42) | 0.000 | -1.51 (-2.15 to -0.87) | 0.000 |
| Northern Mariana Islands | -1.16 (-1.36 to -0.96) | 0.000 | 0.84 (0.56 to 1.11) | 0.000 | -0.25 (-0.81 to 0.31) | 0.376 |
| Norway | -4.12 (-5.19 to -3.04) | 0.000 | -6.02 (-6.19 to -5.86) | 0.000 | -4.45 (-5.39 to -3.51) | 0.000 |
| Oman | -0.81 (-1.02 to -0.59) | 0.000 | -2.58 (-2.77 to -2.4) | 0.000 | -4.86 (-5.77 to -3.94) | 0.000 |
| Pakistan | 3.1 (2.95 to 3.24) | 0.000 | -0.54 (-0.7 to -0.38) | 0.000 | -0.69 (-0.92 to -0.46) | 0.000 |
| Palau | -0.32 (-0.4 to -0.24) | 0.000 | 0.02 (-0.26 to 0.3) | 0.892 | -0.6 (-0.67 to -0.52) | 0.000 |
| Palestine | -1.78 (-2.03 to -1.53) | 0.000 | -3.95 (-4.06 to -3.84) | 0.000 | -0.67 (-1.58 to 0.24) | 0.150 |
| Panama | -5.85 (-9.4 to -2.16) | 0.002 | 2.29 (1.32 to 3.28) | 0.000 | -3.13 (-3.99 to -2.26) | 0.000 |
| Papua New Guinea | 0.94 (0.68 to 1.21) | 0.000 | 1.93 (1.64 to 2.22) | 0.000 | -0.44 (-0.66 to -0.22) | 0.000 |
| Paraguay | -3.27 (-5.41 to -1.08) | 0.004 | -1.04 (-2.07 to 0.01) | 0.051 | -1.1 (-2.67 to 0.49) | 0.174 |
| Peru | -5.19 (-7.36 to -2.96) | 0.000 | -2.38 (-4.55 to -0.17) | 0.035 | -4.14 (-5.29 to -2.98) | 0.000 |
| Philippines | 6.45 (5.18 to 7.73) | 0.000 | 5.4 (4.75 to 6.05) | 0.000 | -0.36 (-0.8 to 0.09) | 0.121 |
| Poland | -5.32 (-6.4 to -4.22) | 0.000 | -5.8 (-7.1 to -4.47) | 0.000 | -4.42 (-6.02 to -2.8) | 0.000 |
| Portugal | -2.91 (-3.22 to -2.61) | 0.000 | -6.3 (-8.08 to -4.49) | 0.000 | -2.64 (-3.33 to -1.95) | 0.000 |
| Puerto Rico | -3.58 (-3.79 to -3.36) | 0.000 | -3.58 (-3.79 to -3.36) | 0.000 | -3.58 (-3.79 to -3.36) | 0.000 |
| Qatar | -5.29 (-7.93 to -2.58) | 0.000 | -4.83 (-8.46 to -1.05) | 0.013 | -2.91 (-4.14 to -1.65) | 0.000 |
| Republic of Korea | -14.44 (-14.94 to -13.95) | 0.000 | -7.28 (-7.76 to -6.81) | 0.000 | -3.2 (-3.75 to -2.66) | 0.000 |
| Republic of Moldova | 1.28 (-2.88 to 5.62) | 0.552 | -1.36 (-3.02 to 0.32) | 0.111 | -4.38 (-5.55 to -3.2) | 0.000 |
| Romania | 2.06 (1.45 to 2.67) | 0.000 | -4.09 (-4.36 to -3.81) | 0.000 | -3.32 (-5.34 to -1.26) | 0.002 |
| Russian Federation | 5.95 (2.28 to 9.76) | 0.001 | -1.56 (-2.33 to -0.79) | 0.000 | -5.28 (-5.95 to -4.61) | 0.000 |
| Rwanda | 0.34 (-0.36 to 1.05) | 0.340 | -6.16 (-6.59 to -5.74) | 0.000 | 0.15 (-0.05 to 0.34) | 0.136 |
| Saint Kitts and Nevis | -6.79 (-7.19 to -6.38) | 0.000 | -7.05 (-7.44 to -6.66) | 0.000 | -3.41 (-3.65 to -3.17) | 0.000 |
| Saint Lucia | -6.4 (-6.71 to -6.09) | 0.000 | -2.61 (-3.28 to -1.94) | 0.000 | -2.18 (-2.91 to -1.45) | 0.000 |
| Saint Vincent and the Grenadines | -1.87 (-2.49 to -1.24) | 0.000 | -3.08 (-3.48 to -2.68) | 0.000 | -0.53 (-1.05 to 0) | 0.052 |
| Samoa | 1.31 (1.19 to 1.43) | 0.000 | 1.29 (1.21 to 1.37) | 0.000 | -0.11 (-0.15 to -0.07) | 0.000 |
| San Marino | -1.77 (-2.09 to -1.46) | 0.000 | -0.17 (-0.38 to 0.04) | 0.107 | -0.21 (-0.33 to -0.1) | 0.001 |
| Sao Tome and Principe | 5.07 (4.5 to 5.64) | 0.000 | -2.2 (-2.64 to -1.75) | 0.000 | -0.61 (-1.34 to 0.12) | 0.099 |
| Saudi Arabia | 0.26 (0.18 to 0.33) | 0.000 | 1.72 (1.3 to 2.14) | 0.000 | -2.48 (-2.7 to -2.25) | 0.000 |
| Senegal | 0.07 (-0.51 to 0.66) | 0.805 | -1.27 (-1.94 to -0.6) | 0.000 | -0.31 (-1.37 to 0.75) | 0.565 |
| Serbia | 0.4 (-1 to 1.83) | 0.576 | -4.82 (-5.15 to -4.5) | 0.000 | -1.99 (-3.33 to -0.64) | 0.004 |
| Seychelles | -2.31 (-2.68 to -1.94) | 0.000 | -0.92 (-1.81 to -0.02) | 0.045 | -1.94 (-2.37 to -1.51) | 0.000 |
| Sierra Leone | 1.41 (1.1 to 1.71) | 0.000 | 1.61 (1.51 to 1.71) | 0.000 | -1.09 (-1.2 to -0.98) | 0.000 |
| Singapore | -2.52 (-3.05 to -1.98) | 0.000 | -4.18 (-5.7 to -2.63) | 0.000 | -3.94 (-4.87 to -3.01) | 0.000 |
| Slovakia | -4.08 (-6.15 to -1.98) | 0.000 | -3.9 (-5.26 to -2.52) | 0.000 | -2.45 (-3.67 to -1.21) | 0.000 |
| Slovenia | -1.42 (-2.87 to 0.04) | 0.057 | -9.14 (-10.14 to -8.13) | 0.000 | -4.21 (-5.23 to -3.18) | 0.000 |
| Solomon Islands | 0.04 (-0.19 to 0.28) | 0.713 | 0.34 (0.2 to 0.48) | 0.000 | -0.79 (-1.02 to -0.56) | 0.000 |
| Somalia | 1.06 (0.87 to 1.25) | 0.000 | 1.55 (1.34 to 1.76) | 0.000 | -0.16 (-0.25 to -0.07) | 0.002 |
| South Africa | -2.49 (-5.27 to 0.38) | 0.089 | -2.43 (-3.46 to -1.38) | 0.000 | -5.95 (-6.85 to -5.04) | 0.000 |
| South Sudan | -0.08 (-0.32 to 0.17) | 0.524 | 1.44 (0.54 to 2.34) | 0.002 | -0.09 (-0.31 to 0.14) | 0.433 |
| Spain | -2.34 (-2.9 to -1.77) | 0.000 | -3.18 (-4.16 to -2.19) | 0.000 | -2.58 (-3.45 to -1.71) | 0.000 |
| Sri Lanka | -1.83 (-3.06 to -0.57) | 0.004 | -3.34 (-4 to -2.68) | 0.000 | -1.45 (-1.91 to -0.98) | 0.000 |
| Sudan | -1.57 (-1.88 to -1.26) | 0.000 | -2.77 (-2.94 to -2.6) | 0.000 | -1.57 (-1.62 to -1.51) | 0.000 |
| Suriname | -1.5 (-4.69 to 1.78) | 0.366 | -3.56 (-6 to -1.07) | 0.005 | 1.16 (0 to 2.34) | 0.050 |
| Sweden | -3.04 (-5 to -1.05) | 0.003 | -4.87 (-5.13 to -4.61) | 0.000 | -3.67 (-5.05 to -2.28) | 0.000 |
| Switzerland | -2.78 (-3.48 to -2.07) | 0.000 | -5.53 (-7.22 to -3.81) | 0.000 | -3.72 (-5.7 to -1.7) | 0.000 |
| Syrian Arab Republic | -3.76 (-4.45 to -3.07) | 0.000 | -3.03 (-3.92 to -2.13) | 0.000 | 0.61 (0.05 to 1.17) | 0.034 |
| Taiwan (Province of China) | -4.37 (-4.72 to -4.02) | 0.000 | -1.63 (-2.91 to -0.33) | 0.014 | -1.25 (-1.73 to -0.77) | 0.000 |
| Tajikistan | 1.39 (0.54 to 2.25) | 0.001 | -2.39 (-2.75 to -2.02) | 0.000 | 1.34 (0.95 to 1.73) | 0.000 |
| Thailand | 1.19 (0.54 to 1.85) | 0.001 | -6.27 (-6.59 to -5.95) | 0.000 | -3.26 (-4.48 to -2.02) | 0.000 |
| Timor-Leste | -0.16 (-1.52 to 1.22) | 0.820 | -0.92 (-1.76 to -0.06) | 0.035 | 3.27 (2.54 to 4.01) | 0.000 |
| Togo | -0.58 (-0.75 to -0.4) | 0.000 | -0.08 (-0.89 to 0.74) | 0.849 | -0.49 (-1.45 to 0.47) | 0.315 |
| Tokelau | 0.52 (0.41 to 0.64) | 0.000 | 0.16 (0.07 to 0.25) | 0.001 | -0.64 (-0.74 to -0.55) | 0.000 |
| Tonga | 0.05 (-0.03 to 0.13) | 0.212 | -1.63 (-1.82 to -1.44) | 0.000 | -0.01 (-0.12 to 0.11) | 0.927 |
| Trinidad and Tobago | -1.06 (-2.19 to 0.08) | 0.069 | -4.51 (-5.72 to -3.28) | 0.000 | -3.32 (-3.94 to -2.69) | 0.000 |
| Tunisia | -0.63 (-0.91 to -0.36) | 0.000 | -2.52 (-2.68 to -2.37) | 0.000 | -1.5 (-1.88 to -1.12) | 0.000 |
| Turkey | -2.97 (-3.56 to -2.37) | 0.000 | -5.96 (-6.53 to -5.39) | 0.000 | -2.05 (-3.47 to -0.61) | 0.005 |
| Turkmenistan | 5.24 (4.03 to 6.47) | 0.000 | -3.33 (-4.89 to -1.76) | 0.000 | -1.02 (-2.14 to 0.11) | 0.074 |
| Tuvalu | 0.05 (-0.17 to 0.26) | 0.684 | 0.31 (0.26 to 0.37) | 0.000 | -0.56 (-0.71 to -0.41) | 0.000 |
| Uganda | 6.47 (5.76 to 7.19) | 0.000 | -2.61 (-3.42 to -1.79) | 0.000 | 0.95 (0.23 to 1.68) | 0.009 |
| Ukraine | 4.88 (-0.75 to 10.83) | 0.091 | 0.43 (-1.44 to 2.35) | 0.652 | 2.87 (0.26 to 5.54) | 0.031 |
| United Arab Emirates | -2.79 (-2.91 to -2.66) | 0.000 | -4.07 (-4.99 to -3.13) | 0.000 | 3.27 (2.25 to 4.31) | 0.000 |
| United Kingdom | -2.87 (-3.1 to -2.65) | 0.000 | -3.63 (-3.84 to -3.42) | 0.000 | -1.37 (-1.7 to -1.04) | 0.000 |
| United Republic of Tanzania | 1.62 (1.15 to 2.09) | 0.000 | 0.22 (-0.56 to 1.02) | 0.577 | 0.09 (-0.21 to 0.4) | 0.546 |
| United States of America | -0.26 (-0.45 to -0.07) | 0.007 | -1.19 (-1.45 to -0.93) | 0.000 | -1.24 (-1.99 to -0.5) | 0.001 |
| United States Virgin Islands | -2.43 (-2.99 to -1.87) | 0.000 | -3.81 (-3.94 to -3.68) | 0.000 | -0.9 (-1.27 to -0.52) | 0.000 |
| Uruguay | -3.65 (-5.43 to -1.83) | 0.000 | -4.42 (-5.59 to -3.23) | 0.000 | -2.41 (-3.13 to -1.69) | 0.000 |
| Uzbekistan | 5.25 (4.39 to 6.11) | 0.000 | 3.06 (1.92 to 4.2) | 0.000 | -1.78 (-2.48 to -1.08) | 0.000 |
| Vanuatu | 3.31 (2.87 to 3.76) | 0.000 | 0.35 (-0.48 to 1.2) | 0.410 | 0.06 (-0.1 to 0.23) | 0.435 |
| Venezuela (Bolivarian Republic of) | -2.36 (-3.36 to -1.36) | 0.000 | -2.34 (-3 to -1.68) | 0.000 | 1.8 (-0.01 to 3.65) | 0.051 |
| Viet Nam | -3.8 (-3.98 to -3.62) | 0.000 | -1.95 (-2.07 to -1.83) | 0.000 | -0.61 (-0.72 to -0.5) | 0.000 |
| Yemen | -1.46 (-1.76 to -1.16) | 0.000 | -1.88 (-1.97 to -1.79) | 0.000 | 0.24 (-0.05 to 0.53) | 0.111 |
| Zambia | 1.41 (0.64 to 2.17) | 0.000 | -2.6 (-2.97 to -2.23) | 0.000 | -2.42 (-2.94 to -1.9) | 0.000 |
| Zimbabwe | -4.08 (-6.37 to -1.74) | 0.001 | 10.5 (9.6 to 11.41) | 0.000 | -0.51 (-1.03 to 0.01) | 0.056 |

**Table S7.** APCs of global and SDI region in ASDR of IHD in WCBA. Abbreviations: APC, annual percentage change; IHD, ischemic heart disease; WCBA, women of childbearing age; SDI, sociodemographic index; CI, confidence interval.

| Ischemic heart disease | | | |
| --- | --- | --- | --- |
| Location | Year | APC (95% CI) | p value |
| Global | 1990 - 1995 | 0.27 (-0.24 to 0.78) | 0.272 |
| Global | 1995 - 2000 | -0.5 (-1.12 to 0.12) | 0.107 |
| Global | 2000 - 2007 | -2.01 (-2.31 to -1.72) | <0.001 |
| Global | 2007 - 2010 | -0.39 (-2.08 to 1.33) | 0.63 |
| Global | 2010 - 2014 | -1.46 (-2.33 to -0.58) | 0.003 |
| Global | 2014 - 2019 | -0.53 (-1.06 to 0) | 0.05 |
| High SDI | 1990 - 1992 | -2.96 (-4.1 to -1.81) | <0.001 |
| High SDI | 1992 - 1998 | -1.8 (-2.05 to -1.55) | <0.001 |
| High SDI | 1998 - 2002 | 0.21 (-0.36 to 0.77) | 0.442 |
| High SDI | 2002 - 2006 | -1.79 (-2.37 to -1.21) | <0.001 |
| High SDI | 2006 - 2013 | -2.53 (-2.78 to -2.28) | <0.001 |
| High SDI | 2013 - 2019 | -0.36 (-0.77 to 0.05) | 0.084 |
| High-middle SDI | 1990 - 1994 | 4.68 (2.79 to 6.6) | <0.001 |
| High-middle SDI | 1994 - 2002 | -1.34 (-1.95 to -0.72) | <0.001 |
| High-middle SDI | 2002 - 2013 | -3.95 (-4.27 to -3.63) | <0.001 |
| High-middle SDI | 2013 - 2019 | -2.12 (-3.08 to -1.15) | <0.001 |
| Low SDI | 1990 - 1995 | 0.67 (0.38 to 0.96) | <0.001 |
| Low SDI | 1995 - 2002 | -0.13 (-0.34 to 0.08) | 0.198 |
| Low SDI | 2002 - 2007 | -1.25 (-1.59 to -0.91) | <0.001 |
| Low SDI | 2007 - 2010 | -0.64 (-1.68 to 0.41) | 0.209 |
| Low SDI | 2010 - 2016 | -1.5 (-1.73 to -1.27) | <0.001 |
| Low SDI | 2016 - 2019 | -0.79 (-1.32 to -0.26) | 0.007 |
| Low-middle SDI | 1990 - 1999 | -0.18 (-0.43 to 0.07) | 0.153 |
| Low-middle SDI | 1999 - 2005 | -2.17 (-2.68 to -1.66) | <0.001 |
| Low-middle SDI | 2005 - 2019 | -0.29 (-0.41 to -0.18) | <0.001 |
| Middle SDI | 1990 - 2000 | -0.65 (-0.82 to -0.48) | <0.001 |
| Middle SDI | 2000 - 2003 | -3.23 (-4.97 to -1.46) | 0.002 |
| Middle SDI | 2003 - 2007 | -1.83 (-2.63 to -1.02) | <0.001 |
| Middle SDI | 2007 - 2010 | 0.23 (-1.44 to 1.92) | 0.774 |
| Middle SDI | 2010 - 2015 | -1.82 (-2.38 to -1.26) | <0.001 |
| Middle SDI | 2015 - 2019 | -0.75 (-1.57 to 0.08) | 0.071 |

**Table S8.** APCs of 204 countries in ASDR of IHD in WCBA. Abbreviations: APC, annual percentage change; IHD, ischemic heart disease; WCBA, women of childbearing age; CI, confidence interval.

| Ischemic heart disease | | | |
| --- | --- | --- | --- |
| Location | Year | APC (95% CI) | p value |
| Afghanistan | 1990 - 1995 | 0.66 (0.52 to 0.81) | 0 |
| Afghanistan | 2001 - 2000 | -2.72 (-2.84 to -2.6) | 0 |
| Afghanistan | 2014 - 2007 | -1.17 (-1.63 to -0.71) | 0 |
| Albania | 1990 - 2010 | -5.89 (-7.79 to -3.95) | 0 |
| Albania | 1994 - 2014 | 1.81 (1.34 to 2.29) | 0 |
| Albania | 2005 - 2019 | -2 (-2.41 to -1.58) | 0 |
| Algeria | 1990 - 1992 | -2.93 (-3.04 to -2.83) | 0 |
| Algeria | 2001 - 1998 | -4.27 (-4.53 to -4.01) | 0 |
| Algeria | 2008 - 2002 | -1.99 (-2.16 to -1.82) | 0 |
| Algeria | 2017 - 2006 | -0.57 (-2.23 to 1.11) | 0.48 |
| American Samoa | 1990 - 2013 | 0.99 (0.77 to 1.21) | 0 |
| American Samoa | 1998 - 2019 | 4.15 (2.21 to 6.13) | 0 |
| American Samoa | 2001 - 1994 | -0.97 (-1.85 to -0.09) | 0.034 |
| American Samoa | 2005 - 2002 | 2.41 (2.24 to 2.58) | 0 |
| American Samoa | 2015 - 2013 | 0.06 (-0.57 to 0.69) | 0.854 |
| Andorra | 1990 - 2019 | 0.1 (-0.56 to 0.77) | 0.75 |
| Andorra | 1994 - 1995 | -3.38 (-3.49 to -3.27) | 0 |
| Andorra | 2007 - 2002 | -0.63 (-0.75 to -0.51) | 0 |
| Angola | 1990 - 2007 | 0.66 (0.36 to 0.95) | 0 |
| Angola | 2004 - 2010 | -3.35 (-3.82 to -2.88) | 0 |
| Angola | 2015 - 2016 | 0.09 (-2.05 to 2.27) | 0.933 |
| Antigua and Barbuda | 1990 - 2019 | -2.82 (-3.08 to -2.56) | 0 |
| Antigua and Barbuda | 1998 - 1999 | -7.25 (-9.61 to -4.84) | 0 |
| Antigua and Barbuda | 2001 - 2005 | -3.96 (-4.28 to -3.64) | 0 |
| Antigua and Barbuda | 2009 - 2019 | -0.88 (-1.71 to -0.04) | 0.041 |
| Antigua and Barbuda | 2014 - 2000 | 0.21 (-0.45 to 0.88) | 0.509 |
| Argentina | 1990 - 2003 | -4.3 (-4.43 to -4.17) | 0 |
| Argentina | 2005 - 2007 | -0.2 (-1.28 to 0.9) | 0.708 |
| Argentina | 2010 - 2010 | -3.62 (-5.3 to -1.9) | 0 |
| Argentina | 2014 - 2015 | -0.27 (-1.2 to 0.66) | 0.548 |
| Armenia | 1990 - 2019 | 6.59 (1.39 to 12.05) | 0.015 |
| Armenia | 1993 - 1995 | -4.63 (-5.59 to -3.65) | 0 |
| Armenia | 2002 - 2000 | 3.12 (-1.52 to 7.98) | 0.179 |
| Armenia | 2006 - 2007 | -2.27 (-2.81 to -1.73) | 0 |
| Australia | 1990 - 2010 | -4.32 (-5.29 to -3.34) | 0 |
| Australia | 1995 - 2014 | 1.16 (-3.54 to 6.1) | 0.617 |
| Australia | 1998 - 2019 | -3.27 (-3.5 to -3.03) | 0 |
| Australia | 2012 - 1992 | -1.57 (-2.3 to -0.83) | 0 |
| Austria | 1990 - 1998 | 2.49 (0.35 to 4.67) | 0.026 |
| Austria | 1994 - 2002 | -3.03 (-4.44 to -1.59) | 0.001 |
| Austria | 2000 - 2006 | -9.8 (-11.24 to -8.34) | 0 |
| Austria | 2006 - 2013 | -0.56 (-4.41 to 3.44) | 0.763 |
| Austria | 2010 - 2019 | -4.8 (-6.12 to -3.46) | 0 |
| Austria | 2017 - 1994 | 0.29 (-8.65 to 10.1) | 0.948 |
| Azerbaijan | 1990 - 2002 | 7.7 (6.24 to 9.18) | 0 |
| Azerbaijan | 1994 - 2013 | -5.24 (-6.11 to -4.37) | 0 |
| Azerbaijan | 2000 - 2019 | -1.98 (-2.35 to -1.6) | 0 |
| Azerbaijan | 2012 - 1995 | -5.81 (-12.86 to 1.81) | 0.122 |
| Azerbaijan | 2015 - 2002 | 0.32 (-2.43 to 3.14) | 0.812 |
| Bahamas | 1990 - 2007 | -2.51 (-3.08 to -1.95) | 0 |
| Bahamas | 1999 - 2010 | -6.85 (-8.71 to -4.95) | 0 |
| Bahamas | 2004 - 2016 | -0.19 (-0.5 to 0.12) | 0.211 |
| Bahrain | 1990 - 2019 | -6.97 (-8.93 to -4.97) | 0 |
| Bahrain | 1995 - 1999 | 6.46 (-2.95 to 16.78) | 0.168 |
| Bahrain | 1998 - 2005 | -2.44 (-10.73 to 6.62) | 0.559 |
| Bahrain | 2001 - 2019 | -15.48 (-22.59 to -7.71) | 0.001 |
| Bahrain | 2004 - 2000 | -7.74 (-9.24 to -6.22) | 0 |
| Bahrain | 2011 - 2003 | 0.27 (-0.97 to 1.52) | 0.649 |
| Bangladesh | 1990 - 2007 | -5.15 (-5.88 to -4.42) | 0 |
| Bangladesh | 1999 - 2010 | 1.45 (0.24 to 2.68) | 0.021 |
| Bangladesh | 2006 - 2015 | -1.22 (-1.61 to -0.82) | 0 |
| Barbados | 1990 - 2019 | -1.93 (-2.78 to -1.08) | 0 |
| Barbados | 1996 - 1995 | -5.89 (-7.44 to -4.31) | 0 |
| Barbados | 2001 - 2000 | 1.06 (-4.15 to 6.56) | 0.678 |
| Barbados | 2004 - 2007 | -3.18 (-3.87 to -2.48) | 0 |
| Barbados | 2012 - 2010 | 0.07 (-0.82 to 0.98) | 0.865 |
| Belarus | 1990 - 2014 | 10.46 (5.33 to 15.85) | 0.001 |
| Belarus | 1993 - 2019 | 1.31 (0.7 to 1.92) | 0 |
| Belarus | 2005 - 1992 | -5.33 (-13.16 to 3.2) | 0.193 |
| Belarus | 2008 - 1998 | 1.49 (-6.74 to 10.45) | 0.711 |
| Belarus | 2011 - 2002 | -9.67 (-14.44 to -4.64) | 0.001 |
| Belarus | 2015 - 2006 | -0.71 (-6.6 to 5.55) | 0.805 |
| Belgium | 1990 - 2013 | -6.02 (-8.85 to -3.1) | 0 |
| Belgium | 1993 - 2019 | -1.39 (-3.19 to 0.45) | 0.129 |
| Belgium | 1998 - 1994 | -4.47 (-4.7 to -4.25) | 0 |
| Belgium | 2016 - 2002 | -0.03 (-4.15 to 4.27) | 0.99 |
| Belize | 1990 - 2013 | -1.65 (-5.17 to 2) | 0.352 |
| Belize | 1994 - 2019 | 8.47 (-2.23 to 20.33) | 0.118 |
| Belize | 1997 - 1995 | -8.69 (-18.22 to 1.95) | 0.1 |
| Belize | 2000 - 2002 | -3.04 (-3.39 to -2.68) | 0 |
| Benin | 1990 - 2007 | 0.73 (0.21 to 1.25) | 0.01 |
| Benin | 1993 - 2010 | 2.82 (1.76 to 3.89) | 0 |
| Benin | 1996 - 2016 | 0.21 (-0.3 to 0.73) | 0.395 |
| Benin | 2000 - 2019 | -1.47 (-1.62 to -1.33) | 0 |
| Benin | 2008 - 1999 | 0.35 (0.23 to 0.47) | 0 |
| Benin | 2017 - 2005 | -2.11 (-3.2 to -1.02) | 0.001 |
| Bermuda | 1990 - 2019 | -0.22 (-2.9 to 2.54) | 0.866 |
| Bermuda | 1992 - 2000 | -5.23 (-6.09 to -4.36) | 0 |
| Bermuda | 1997 - 2003 | -8.92 (-9.76 to -8.07) | 0 |
| Bermuda | 2002 - 2007 | -5.87 (-6.35 to -5.38) | 0 |
| Bermuda | 2009 - 2010 | -3.99 (-5.02 to -2.94) | 0 |
| Bermuda | 2014 - 2015 | 0.13 (-0.71 to 0.98) | 0.742 |
| Bhutan | 1990 - 2019 | 0.94 (0.34 to 1.54) | 0.004 |
| Bhutan | 1995 - 1995 | -2.55 (-2.67 to -2.43) | 0 |
| Bhutan | 2009 - 2000 | -1.16 (-1.36 to -0.97) | 0 |
| Bolivia (Plurinational State of) | 1990 - 2007 | -0.17 (-0.92 to 0.58) | 0.64 |
| Bolivia (Plurinational State of) | 1994 - 2010 | -4.27 (-4.52 to -4.01) | 0 |
| Bolivia (Plurinational State of) | 2003 - 2014 | -1.4 (-1.49 to -1.31) | 0 |
| Bosnia and Herzegovina | 1990 - 2019 | 5.59 (1.7 to 9.62) | 0.007 |
| Bosnia and Herzegovina | 1993 - 1992 | -4.08 (-4.41 to -3.74) | 0 |
| Bosnia and Herzegovina | 2011 - 1998 | -0.94 (-2.07 to 0.2) | 0.101 |
| Botswana | 1990 - 2002 | 0.94 (-0.12 to 2.01) | 0.078 |
| Botswana | 1994 - 2006 | -3.79 (-7.64 to 0.22) | 0.062 |
| Botswana | 1997 - 2013 | 7.04 (6 to 8.09) | 0 |
| Botswana | 2003 - 2019 | -6.7 (-10.42 to -2.82) | 0.003 |
| Botswana | 2006 - 1994 | 3.54 (2.71 to 4.37) | 0 |
| Botswana | 2012 - 2002 | -1.55 (-1.99 to -1.11) | 0 |
| Brazil | 1990 - 2013 | -1.76 (-2.29 to -1.24) | 0 |
| Brazil | 1996 - 2019 | -3.02 (-3.33 to -2.71) | 0 |
| Brazil | 2005 - 1995 | -1.56 (-2.98 to -0.12) | 0.036 |
| Brazil | 2009 - 2002 | -2.56 (-2.82 to -2.29) | 0 |
| Brunei Darussalam | 1990 - 2007 | -6.66 (-7.07 to -6.25) | 0 |
| Brunei Darussalam | 1995 - 2010 | -8.94 (-10.58 to -7.28) | 0 |
| Brunei Darussalam | 1998 - 2016 | -4.51 (-6.17 to -2.81) | 0 |
| Brunei Darussalam | 2001 - 2019 | 0.69 (-1 to 2.4) | 0.396 |
| Brunei Darussalam | 2004 - 1999 | -1.95 (-2.13 to -1.77) | 0 |
| Brunei Darussalam | 2013 - 2005 | 1.39 (1.07 to 1.71) | 0 |
| Bulgaria | 1990 - 2019 | 6.48 (5.54 to 7.42) | 0 |
| Bulgaria | 1997 - 2000 | -1.51 (-7.42 to 4.78) | 0.613 |
| Bulgaria | 2000 - 2003 | -4.61 (-5 to -4.21) | 0 |
| Bulgaria | 2013 - 2007 | 0.8 (-1.18 to 2.83) | 0.41 |
| Burkina Faso | 1990 - 2010 | -1.5 (-2.72 to -0.26) | 0.022 |
| Burkina Faso | 1994 - 2015 | 1.29 (0.04 to 2.56) | 0.044 |
| Burkina Faso | 1999 - 2019 | -0.71 (-1.36 to -0.06) | 0.035 |
| Burkina Faso | 2006 - 1995 | -1.8 (-3.02 to -0.56) | 0.008 |
| Burkina Faso | 2011 - 2000 | 1.75 (0.84 to 2.67) | 0.001 |
| Burkina Faso | 2017 - 2007 | -0.75 (-4.65 to 3.32) | 0.693 |
| Burundi | 1990 - 2010 | 2.68 (2.25 to 3.12) | 0 |
| Burundi | 1996 - 2014 | -0.52 (-2.8 to 1.82) | 0.637 |
| Burundi | 1999 - 2019 | -1.82 (-2.31 to -1.34) | 0 |
| Burundi | 2005 - 1992 | -3.69 (-5.75 to -1.59) | 0.002 |
| Burundi | 2008 - 1998 | -1.31 (-1.54 to -1.07) | 0 |
| Burundi | 2017 - 2002 | 0.02 (-2.19 to 2.28) | 0.986 |
| Cabo Verde | 1990 - 2006 | 4.17 (3.64 to 4.71) | 0 |
| Cabo Verde | 1998 - 2013 | -2.32 (-2.67 to -1.97) | 0 |
| Cabo Verde | 2009 - 2019 | -0.04 (-2.04 to 2) | 0.967 |
| Cabo Verde | 2013 - 1994 | -2.99 (-3.74 to -2.24) | 0 |
| Cambodia | 1990 - 2002 | -0.13 (-0.43 to 0.17) | 0.368 |
| Cambodia | 1995 - 2013 | -1.12 (-1.77 to -0.46) | 0.003 |
| Cambodia | 1999 - 2019 | -1.86 (-3.13 to -0.57) | 0.008 |
| Cambodia | 2002 - 1995 | -3.69 (-3.9 to -3.48) | 0 |
| Cambodia | 2009 - 2002 | -1.8 (-2.43 to -1.17) | 0 |
| Cambodia | 2013 - 2007 | -0.79 (-1.01 to -0.58) | 0 |
| Cameroon | 1990 - 2010 | 1.21 (0.86 to 1.56) | 0 |
| Cameroon | 1995 - 2016 | 4.04 (3.55 to 4.53) | 0 |
| Cameroon | 2000 - 2019 | 1.88 (0.4 to 3.39) | 0.016 |
| Cameroon | 2003 - 1999 | -1.24 (-1.56 to -0.91) | 0 |
| Cameroon | 2009 - 2005 | -0.69 (-1.05 to -0.34) | 0.001 |
| Cameroon | 2015 - 2019 | -2.41 (-2.92 to -1.89) | 0 |
| Canada | 1990 - 2000 | -2.36 (-2.47 to -2.26) | 0 |
| Canada | 2009 - 2003 | -4.73 (-8.1 to -1.24) | 0.011 |
| Canada | 2012 - 2007 | -0.91 (-1.52 to -0.29) | 0.006 |
| Central African Republic | 1990 - 2010 | -2.66 (-5.35 to 0.1) | 0.058 |
| Central African Republic | 1992 - 2015 | 1.99 (1.46 to 2.52) | 0 |
| Central African Republic | 1999 - 2019 | -0.09 (-0.37 to 0.2) | 0.521 |
| Central African Republic | 2009 - 1995 | 1.54 (0.07 to 3.03) | 0.041 |
| Central African Republic | 2013 - 2000 | -0.7 (-1.18 to -0.21) | 0.008 |
| Chad | 1990 - 2007 | 1.44 (1.28 to 1.6) | 0 |
| Chad | 2004 - 2010 | -0.43 (-0.57 to -0.28) | 0 |
| Chile | 1990 - 2014 | -7.39 (-14.18 to -0.06) | 0.048 |
| Chile | 1992 - 2019 | -2.73 (-3.39 to -2.07) | 0 |
| Chile | 2002 - 1992 | -1.34 (-1.62 to -1.06) | 0 |
| China | 1990 - 1998 | 0.73 (0.17 to 1.29) | 0.013 |
| China | 2000 - 2002 | -8.73 (-13.67 to -3.51) | 0.003 |
| China | 2003 - 2006 | -4.08 (-6.49 to -1.62) | 0.003 |
| China | 2007 - 2013 | -0.22 (-4.92 to 4.72) | 0.925 |
| China | 2010 - 2019 | -2.86 (-3.5 to -2.21) | 0 |
| Colombia | 1990 - 1994 | -2.24 (-4.21 to -0.23) | 0.031 |
| Colombia | 1993 - 2002 | -5.9 (-6.4 to -5.4) | 0 |
| Colombia | 2001 - 2013 | -3.28 (-3.6 to -2.96) | 0 |
| Colombia | 2012 - 2019 | 0.21 (-0.79 to 1.22) | 0.672 |
| Comoros | 1990 - 1995 | 1.97 (0.81 to 3.14) | 0.002 |
| Comoros | 2001 - 2002 | -2.28 (-3.28 to -1.27) | 0 |
| Comoros | 2012 - 2007 | 10 (-3.74 to 25.69) | 0.151 |
| Comoros | 2015 - 2010 | -3.48 (-7.73 to 0.97) | 0.116 |
| Congo | 1990 - 2016 | -2.31 (-6.83 to 2.43) | 0.312 |
| Congo | 1992 - 2019 | 5.29 (3.79 to 6.81) | 0 |
| Congo | 1997 - 1999 | -2.8 (-4.12 to -1.45) | 0 |
| Congo | 2002 - 2005 | 2.06 (-0.02 to 4.18) | 0.052 |
| Congo | 2006 - 2019 | -2.22 (-2.44 to -2) | 0 |
| Cook Islands | 1990 - 2000 | -4.3 (-4.98 to -3.63) | 0 |
| Cook Islands | 1994 - 2003 | -3.32 (-3.46 to -3.18) | 0 |
| Cook Islands | 2005 - 2007 | 0.18 (-1.53 to 1.91) | 0.832 |
| Cook Islands | 2008 - 2010 | 1.48 (0.93 to 2.03) | 0 |
| Cook Islands | 2013 - 2015 | -0.9 (-1.25 to -0.56) | 0 |
| Costa Rica | 1990 - 2019 | -4.24 (-4.69 to -3.78) | 0 |
| Costa Rica | 2008 - 1995 | 0.3 (-1.04 to 1.65) | 0.652 |
| Coted'Ivoire | 1990 - 2000 | -1.94 (-3.84 to -0.01) | 0.049 |
| Coted'Ivoire | 1992 - 2007 | 1.09 (0.43 to 1.74) | 0.003 |
| Coted'Ivoire | 1997 - 2010 | 2.72 (2.06 to 3.39) | 0 |
| Coted'Ivoire | 2002 - 2014 | -0.93 (-1.06 to -0.79) | 0 |
| Coted'Ivoire | 2014 - 2019 | -2.49 (-2.96 to -2.03) | 0 |
| Croatia | 1990 - 1992 | -1.68 (-3.15 to -0.18) | 0.03 |
| Croatia | 1996 - 1998 | -7.49 (-15.06 to 0.76) | 0.072 |
| Croatia | 1999 - 2002 | -2.8 (-3.45 to -2.15) | 0 |
| Croatia | 2010 - 2006 | -6.07 (-7.16 to -4.97) | 0 |
| Cuba | 1990 - 2013 | -3.21 (-4.18 to -2.23) | 0 |
| Cuba | 1996 - 2019 | -6.76 (-8.1 to -5.4) | 0 |
| Cuba | 2002 - 1994 | -4.53 (-5.15 to -3.9) | 0 |
| Cuba | 2012 - 2002 | -0.04 (-1.42 to 1.35) | 0.95 |
| Cyprus | 1990 - 2013 | -1.02 (-1.7 to -0.34) | 0.006 |
| Cyprus | 1999 - 2019 | -11.85 (-17.88 to -5.38) | 0.002 |
| Cyprus | 2002 - 1995 | -1.28 (-7.64 to 5.53) | 0.688 |
| Cyprus | 2005 - 2002 | -5.52 (-5.99 to -5.05) | 0 |
| Cyprus | 2016 - 2007 | 0.56 (-3.02 to 4.27) | 0.75 |
| Czechia | 1990 - 2010 | -5.29 (-5.53 to -5.06) | 0 |
| Czechia | 2010 - 2016 | -3.49 (-4.73 to -2.24) | 0 |
| Democratic People's Republic of Korea | 1990 - 2019 | 0.22 (-0.25 to 0.68) | 0.331 |
| Democratic People's Republic of Korea | 1993 - 1999 | 1.4 (1.2 to 1.6) | 0 |
| Democratic People's Republic of Korea | 1999 - 2005 | 1.83 (1.42 to 2.25) | 0 |
| Democratic People's Republic of Korea | 2003 - 2019 | -0.26 (-0.43 to -0.09) | 0.006 |
| Democratic People's Republic of Korea | 2009 - 2000 | -1.18 (-1.28 to -1.07) | 0 |
| Democratic People's Republic of Korea | 2017 - 2003 | -0.45 (-1.3 to 0.41) | 0.279 |
| Democratic Republic of the Congo | 1990 - 2007 | -0.01 (-0.42 to 0.4) | 0.959 |
| Democratic Republic of the Congo | 1998 - 2010 | 1.31 (0.17 to 2.46) | 0.026 |
| Democratic Republic of the Congo | 2003 - 2015 | -1.75 (-1.98 to -1.52) | 0 |
| Democratic Republic of the Congo | 2015 - 2019 | -0.05 (-1.18 to 1.1) | 0.931 |
| Denmark | 1990 - 1995 | -2.21 (-3.5 to -0.91) | 0.003 |
| Denmark | 1995 - 2000 | -6.61 (-12.55 to -0.27) | 0.043 |
| Denmark | 1998 - 2007 | -3.33 (-6.36 to -0.2) | 0.039 |
| Denmark | 2002 - 2010 | -7.8 (-8.85 to -6.74) | 0 |
| Denmark | 2009 - 2014 | -9.62 (-11.8 to -7.39) | 0 |
| Denmark | 2014 - 2019 | 0.96 (-1.21 to 3.17) | 0.36 |
| Djibouti | 1990 - 1992 | 2.08 (1.82 to 2.33) | 0 |
| Djibouti | 2005 - 1998 | -0.48 (-0.79 to -0.16) | 0.005 |
| Dominica | 1990 - 2002 | -3.35 (-3.89 to -2.81) | 0 |
| Dominica | 1998 - 2006 | -0.54 (-2.11 to 1.06) | 0.488 |
| Dominica | 2003 - 2013 | -4.19 (-5.3 to -3.07) | 0 |
| Dominica | 2009 - 2019 | -1.11 (-1.56 to -0.66) | 0 |
| Dominican Republic | 1990 - 1994 | 0.3 (-0.26 to 0.87) | 0.27 |
| Dominican Republic | 1997 - 2002 | -2.8 (-4.76 to -0.8) | 0.01 |
| Dominican Republic | 2001 - 2013 | 4.58 (3.19 to 5.98) | 0 |
| Dominican Republic | 2006 - 2019 | -2.26 (-3.84 to -0.66) | 0.009 |
| Dominican Republic | 2011 - 1995 | 6.03 (3.58 to 8.55) | 0 |
| Dominican Republic | 2015 - 2002 | -2.77 (-4.75 to -0.75) | 0.011 |
| Ecuador | 1990 - 2007 | -3.68 (-4.19 to -3.17) | 0 |
| Ecuador | 2001 - 2010 | 3.65 (-5.12 to 13.23) | 0.407 |
| Ecuador | 2004 - 2016 | -3.86 (-4.82 to -2.89) | 0 |
| Ecuador | 2013 - 2019 | 0.42 (-1.56 to 2.44) | 0.663 |
| Egypt | 1990 - 1999 | -2.88 (-3.22 to -2.53) | 0 |
| Egypt | 2004 - 2005 | -0.3 (-0.69 to 0.09) | 0.122 |
| El Salvador | 1990 - 2019 | -1.21 (-4.48 to 2.18) | 0.462 |
| El Salvador | 1994 - 2000 | -5.98 (-7.93 to -3.99) | 0 |
| El Salvador | 2000 - 2003 | -2.35 (-2.72 to -1.98) | 0 |
| Equatorial Guinea | 1990 - 2007 | -0.72 (-1.72 to 0.28) | 0.147 |
| Equatorial Guinea | 1996 - 2010 | -4.92 (-5.94 to -3.89) | 0 |
| Equatorial Guinea | 2003 - 2015 | -2.96 (-3.5 to -2.41) | 0 |
| Equatorial Guinea | 2014 - 2019 | -0.04 (-1.69 to 1.64) | 0.958 |
| Eritrea | 1990 - 1995 | -1.53 (-2.4 to -0.65) | 0.002 |
| Eritrea | 1994 - 2000 | 0.34 (-0.96 to 1.66) | 0.591 |
| Eritrea | 1998 - 2007 | 3.49 (3.06 to 3.93) | 0 |
| Eritrea | 2005 - 2010 | 0.31 (0.2 to 0.42) | 0 |
| Estonia | 1990 - 2014 | 11.11 (7.19 to 15.18) | 0 |
| Estonia | 1994 - 2019 | -9.83 (-19.66 to 1.21) | 0.075 |
| Estonia | 1997 - 1992 | 0.7 (-5.06 to 6.81) | 0.802 |
| Estonia | 2001 - 1998 | -14.18 (-24.07 to -3) | 0.018 |
| Estonia | 2004 - 2002 | -9.74 (-10.94 to -8.52) | 0 |
| Estonia | 2014 - 2006 | 1.24 (-3.54 to 6.25) | 0.592 |
| Eswatini | 1990 - 2013 | -1.4 (-1.9 to -0.89) | 0 |
| Eswatini | 1998 - 2019 | 13.24 (10.24 to 16.31) | 0 |
| Eswatini | 2002 - 1994 | 4.63 (-0.88 to 10.45) | 0.095 |
| Eswatini | 2005 - 2002 | -2.13 (-4.85 to 0.67) | 0.125 |
| Eswatini | 2009 - 2013 | -4.37 (-4.82 to -3.93) | 0 |
| Ethiopia | 1990 - 2019 | -0.21 (-0.55 to 0.14) | 0.216 |
| Ethiopia | 1996 - 1995 | -2.36 (-4.13 to -0.55) | 0.015 |
| Ethiopia | 1999 - 2002 | -7.13 (-8.76 to -5.47) | 0 |
| Ethiopia | 2002 - 2007 | -5.06 (-5.2 to -4.92) | 0 |
| Ethiopia | 2011 - 2010 | -2.97 (-4.2 to -1.73) | 0 |
| Ethiopia | 2014 - 2016 | -0.95 (-1.3 to -0.59) | 0 |
| Fiji | 1990 - 2019 | -0.59 (-0.83 to -0.35) | 0 |
| Fiji | 2000 - 1999 | -3.76 (-4.88 to -2.63) | 0 |
| Fiji | 2004 - 2005 | 3.96 (2.86 to 5.07) | 0 |
| Fiji | 2008 - 2019 | -4.04 (-6.12 to -1.91) | 0.001 |
| Fiji | 2011 - 2000 | -0.58 (-0.92 to -0.24) | 0.002 |
| Finland | 1990 - 2003 | -5.42 (-7.02 to -3.79) | 0 |
| Finland | 1994 - 2007 | -0.54 (-1.8 to 0.74) | 0.383 |
| Finland | 2000 - 2010 | -5.5 (-5.92 to -5.09) | 0 |
| Finland | 2012 - 2015 | -7.81 (-15.06 to 0.06) | 0.051 |
| Finland | 2015 - 2019 | 0.71 (-2.17 to 3.67) | 0.614 |
| France | 1990 - 1995 | -1.02 (-1.4 to -0.63) | 0 |
| France | 1997 - 2000 | 1.21 (0.27 to 2.16) | 0.015 |
| France | 2002 - 2007 | -2.1 (-2.47 to -1.72) | 0 |
| France | 2010 - 2010 | -4.42 (-4.96 to -3.89) | 0 |
| France | 2017 - 2014 | 1.08 (-3.25 to 5.6) | 0.611 |
| Gabon | 1990 - 2019 | -1.64 (-3.54 to 0.3) | 0.092 |
| Gabon | 1993 - 1992 | 2.52 (2.17 to 2.87) | 0 |
| Gabon | 2003 - 1998 | -2.57 (-3.74 to -1.37) | 0 |
| Gabon | 2008 - 2002 | -4.82 (-6.03 to -3.59) | 0 |
| Gabon | 2013 - 2006 | -1.44 (-2.15 to -0.73) | 0.001 |
| Gambia | 1990 - 2013 | 1.1 (0.51 to 1.69) | 0.001 |
| Gambia | 2002 - 2019 | 6.2 (-3.29 to 16.62) | 0.194 |
| Gambia | 2005 - 1994 | -1.51 (-3.04 to 0.05) | 0.057 |
| Gambia | 2012 - 2002 | 2.69 (1.39 to 4.01) | 0 |
| Georgia | 1990 - 2013 | 5.85 (3.58 to 8.18) | 0 |
| Georgia | 1994 - 2019 | -8.43 (-14.32 to -2.13) | 0.013 |
| Georgia | 1997 - 1995 | -1.73 (-3.82 to 0.41) | 0.104 |
| Georgia | 2002 - 2002 | -5.6 (-7.23 to -3.95) | 0 |
| Georgia | 2008 - 2007 | -14.82 (-16.53 to -13.09) | 0 |
| Georgia | 2014 - 2010 | 2.34 (-0.24 to 4.99) | 0.072 |
| Germany | 1990 - 2016 | -4.84 (-5.36 to -4.32) | 0 |
| Germany | 1996 - 2019 | -3.11 (-3.62 to -2.6) | 0 |
| Germany | 2003 - 1999 | -4.93 (-5.17 to -4.68) | 0 |
| Germany | 2014 - 2005 | -0.01 (-0.99 to 0.99) | 0.99 |
| Ghana | 1990 - 2019 | -0.73 (-0.84 to -0.62) | 0 |
| Ghana | 2007 - 2000 | -1.92 (-2.1 to -1.74) | 0 |
| Greece | 1990 - 2003 | 0.73 (0.55 to 0.9) | 0 |
| Greece | 2011 - 2007 | -6.27 (-9.33 to -3.12) | 0.001 |
| Greece | 2015 - 2010 | 1.26 (-0.92 to 3.48) | 0.247 |
| Greenland | 1990 - 2015 | -1.94 (-2.22 to -1.65) | 0 |
| Greenland | 1999 - 2019 | -5.25 (-8.04 to -2.38) | 0.001 |
| Greenland | 2002 - 1995 | -2.22 (-5.16 to 0.81) | 0.139 |
| Greenland | 2005 - 2000 | -5.94 (-6.33 to -5.55) | 0 |
| Greenland | 2013 - 2007 | -1.84 (-2.4 to -1.27) | 0 |
| Grenada | 1990 - 2010 | -0.19 (-1.68 to 1.32) | 0.788 |
| Grenada | 1993 - 2014 | -6.16 (-9.18 to -3.05) | 0.001 |
| Grenada | 1996 - 2019 | -8.42 (-9.85 to -6.97) | 0 |
| Grenada | 2000 - 1992 | -2.38 (-2.71 to -2.05) | 0 |
| Grenada | 2009 - 1998 | 0.31 (-0.11 to 0.74) | 0.136 |
| Grenada | 2017 - 2002 | -2.68 (-6.23 to 1) | 0.138 |
| Guam | 1990 - 2006 | 6.65 (4.88 to 8.45) | 0 |
| Guam | 1995 - 2013 | 1.31 (1.17 to 1.44) | 0 |
| Guatemala | 1990 - 2019 | 0.76 (-4.6 to 6.41) | 0.773 |
| Guatemala | 1993 - 1994 | -11.52 (-20.76 to -1.21) | 0.032 |
| Guatemala | 1996 - 2002 | -2.39 (-12.54 to 8.93) | 0.646 |
| Guatemala | 1999 - 2013 | -10.22 (-15.14 to -5.02) | 0.001 |
| Guatemala | 2003 - 2019 | -1.43 (-1.89 to -0.97) | 0 |
| Guinea | 1990 - 1995 | -0.35 (-0.55 to -0.14) | 0.003 |
| Guinea | 1997 - 2002 | 1.05 (0.79 to 1.3) | 0 |
| Guinea | 2004 - 2007 | 3.38 (1.86 to 4.92) | 0 |
| Guinea | 2007 - 2010 | 1.06 (0.86 to 1.26) | 0 |
| Guinea | 2015 - 2016 | -1.48 (-1.95 to -1.01) | 0 |
| Guinea-Bissau | 1990 - 2019 | 1.07 (0.99 to 1.15) | 0 |
| Guinea-Bissau | 2001 - 1999 | 2.33 (1.22 to 3.46) | 0.001 |
| Guinea-Bissau | 2004 - 2005 | 0.81 (0.47 to 1.16) | 0 |
| Guinea-Bissau | 2009 - 2019 | -1.66 (-2.18 to -1.14) | 0 |
| Guinea-Bissau | 2013 - 2000 | -0.63 (-1.7 to 0.45) | 0.229 |
| Guinea-Bissau | 2016 - 2003 | -2.21 (-2.74 to -1.67) | 0 |
| Guyana | 1990 - 2007 | 0.9 (-0.28 to 2.1) | 0.125 |
| Guyana | 1996 - 2010 | -2.34 (-3.89 to -0.76) | 0.007 |
| Guyana | 2002 - 2015 | 1.78 (-5.14 to 9.19) | 0.603 |
| Guyana | 2005 - 2019 | -4.18 (-5.66 to -2.67) | 0 |
| Guyana | 2011 - 1995 | -1.95 (-2.92 to -0.97) | 0.001 |
| Haiti | 1990 - 2000 | -2.31 (-2.99 to -1.63) | 0 |
| Haiti | 1994 - 2007 | -3.6 (-4.29 to -2.91) | 0 |
| Haiti | 1999 - 2010 | 0.8 (0.24 to 1.36) | 0.007 |
| Haiti | 2005 - 2014 | -0.99 (-1.12 to -0.86) | 0 |
| Honduras | 1990 - 2019 | -1.67 (-1.96 to -1.38) | 0 |
| Honduras | 2007 - 1992 | -4.16 (-6.15 to -2.12) | 0 |
| Honduras | 2013 - 1998 | -0.44 (-2.12 to 1.26) | 0.592 |
| Hungary | 1990 - 2002 | 2.57 (-1.19 to 6.48) | 0.171 |
| Hungary | 1993 - 2006 | -3.95 (-4.33 to -3.57) | 0 |
| Hungary | 2007 - 2013 | -6.59 (-7.41 to -5.76) | 0 |
| Hungary | 2016 - 2019 | 0.66 (-6.47 to 8.33) | 0.854 |
| Iceland | 1990 - 1994 | -2.87 (-3.15 to -2.6) | 0 |
| Iceland | 1997 - 2002 | -5.47 (-6.02 to -4.91) | 0 |
| Iceland | 2002 - 2013 | -6.66 (-7.02 to -6.31) | 0 |
| Iceland | 2009 - 2019 | -1.89 (-2.07 to -1.7) | 0 |
| India | 1990 - 1995 | -0.98 (-1.89 to -0.06) | 0.039 |
| India | 1995 - 2002 | 1.34 (-0.58 to 3.29) | 0.158 |
| India | 1999 - 2007 | -3.01 (-3.76 to -2.26) | 0 |
| India | 2005 - 2010 | 1.48 (0.78 to 2.2) | 0.001 |
| India | 2011 - 2016 | -1.98 (-3.46 to -0.47) | 0.014 |
| India | 2015 - 2019 | -0.43 (-1.84 to 1) | 0.53 |
| Indonesia | 1990 - 1999 | -1.46 (-3.02 to 0.12) | 0.067 |
| Indonesia | 1992 - 2005 | -0.13 (-0.38 to 0.12) | 0.29 |
| Indonesia | 1999 - 2019 | 1.33 (-0.15 to 2.83) | 0.074 |
| Indonesia | 2002 - 2000 | -0.76 (-0.85 to -0.67) | 0 |
| Indonesia | 2015 - 2003 | -2.54 (-3.12 to -1.96) | 0 |
| Iran (Islamic Republic of) | 1990 - 2007 | -1.63 (-1.73 to -1.52) | 0 |
| Iran (Islamic Republic of) | 2000 - 2010 | -4.27 (-5.2 to -3.33) | 0 |
| Iran (Islamic Republic of) | 2003 - 2015 | -8.82 (-9.22 to -8.43) | 0 |
| Iran (Islamic Republic of) | 2007 - 2019 | -5.87 (-6.69 to -5.04) | 0 |
| Iran (Islamic Republic of) | 2010 - 1995 | -1.68 (-1.78 to -1.58) | 0 |
| Iraq | 1990 - 2000 | 1.84 (1.58 to 2.1) | 0 |
| Iraq | 1997 - 2007 | -2.66 (-2.81 to -2.51) | 0 |
| Iraq | 2008 - 2010 | -5.46 (-5.87 to -5.05) | 0 |
| Iraq | 2014 - 2014 | -2.21 (-2.66 to -1.75) | 0 |
| Ireland | 1990 - 2019 | -2.63 (-3.24 to -2.03) | 0 |
| Ireland | 2000 - 1992 | -6.58 (-13.82 to 1.26) | 0.092 |
| Ireland | 2003 - 1998 | -3.35 (-5.04 to -1.63) | 0.001 |
| Ireland | 2009 - 2002 | -7.82 (-10.54 to -5.02) | 0 |
| Ireland | 2014 - 2006 | -2.65 (-4.8 to -0.45) | 0.022 |
| Israel | 1990 - 2013 | -8.65 (-9.08 to -8.21) | 0 |
| Israel | 2000 - 2019 | -6.62 (-7.03 to -6.2) | 0 |
| Israel | 2013 - 1994 | -1.23 (-2.59 to 0.15) | 0.078 |
| Italy | 1990 - 2002 | -3.4 (-3.47 to -3.33) | 0 |
| Italy | 2014 - 2013 | -1.2 (-2.18 to -0.22) | 0.019 |
| Jamaica | 1990 - 2019 | 0.27 (-0.64 to 1.18) | 0.541 |
| Jamaica | 2000 - 1995 | -4.72 (-7.85 to -1.47) | 0.007 |
| Jamaica | 2005 - 2002 | 3.19 (1.46 to 4.95) | 0.001 |
| Jamaica | 2012 - 2007 | 8.91 (-2.12 to 21.19) | 0.11 |
| Jamaica | 2015 - 2010 | -3.32 (-8.28 to 1.91) | 0.193 |
| Japan | 1990 - 2016 | -2.12 (-2.92 to -1.31) | 0 |
| Japan | 1994 - 2019 | 0.08 (-1.31 to 1.49) | 0.903 |
| Japan | 1998 - 1999 | -1.11 (-1.32 to -0.9) | 0 |
| Japan | 2011 - 2005 | -4.03 (-4.85 to -3.2) | 0 |
| Japan | 2017 - 2019 | -0.27 (-4.2 to 3.82) | 0.887 |
| Jordan | 1990 - 2000 | -0.39 (-1.08 to 0.3) | 0.243 |
| Jordan | 1998 - 2003 | -2.55 (-3.71 to -1.38) | 0 |
| Jordan | 2004 - 2007 | -13.72 (-15.53 to -11.86) | 0 |
| Jordan | 2008 - 2010 | -7.34 (-11.18 to -3.35) | 0.001 |
| Jordan | 2011 - 2015 | -1.52 (-2.1 to -0.94) | 0 |
| Kazakhstan | 1990 - 2019 | 15.81 (14.67 to 16.96) | 0 |
| Kazakhstan | 1995 - 1995 | -1.94 (-2.3 to -1.58) | 0 |
| Kazakhstan | 2006 - 2000 | -14.43 (-21.54 to -6.67) | 0.002 |
| Kazakhstan | 2009 - 2007 | -8.17 (-9.38 to -6.96) | 0 |
| Kazakhstan | 2017 - 2010 | 1.52 (-8.64 to 12.79) | 0.766 |
| Kenya | 1990 - 2014 | 3.06 (2.73 to 3.4) | 0 |
| Kenya | 1996 - 2019 | 4.88 (2.92 to 6.88) | 0 |
| Kenya | 1999 - 1992 | 6.73 (6.13 to 7.32) | 0 |
| Kenya | 2004 - 1998 | 0.97 (-0.6 to 2.56) | 0.211 |
| Kenya | 2007 - 2002 | -1.04 (-1.15 to -0.94) | 0 |
| Kiribati | 1990 - 2006 | 0.01 (-0.3 to 0.32) | 0.946 |
| Kiribati | 1993 - 2013 | -1.59 (-2.16 to -1.03) | 0 |
| Kiribati | 1996 - 2019 | 0.3 (-0.27 to 0.88) | 0.276 |
| Kiribati | 1999 - 1994 | -1.05 (-1.18 to -0.92) | 0 |
| Kiribati | 2005 - 2002 | 0.33 (0.13 to 0.53) | 0.003 |
| Kiribati | 2010 - 2013 | -0.76 (-0.82 to -0.7) | 0 |
| Kuwait | 1990 - 2019 | 1.48 (-1.15 to 4.18) | 0.256 |
| Kuwait | 1995 - 1995 | -4.87 (-5.34 to -4.4) | 0 |
| Kuwait | 2010 - 2002 | -12.31 (-17.42 to -6.9) | 0 |
| Kuwait | 2014 - 2007 | -0.67 (-4.78 to 3.62) | 0.743 |
| Kyrgyzstan | 1990 - 2010 | 3.86 (-2.78 to 10.95) | 0.242 |
| Kyrgyzstan | 1992 - 2016 | 12.04 (5.21 to 19.32) | 0.001 |
| Kyrgyzstan | 1995 - 2019 | -8.75 (-11.33 to -6.11) | 0 |
| Kyrgyzstan | 1999 - 1999 | -0.18 (-1.13 to 0.79) | 0.7 |
| Kyrgyzstan | 2006 - 2005 | -4.49 (-4.83 to -4.15) | 0 |
| Lao People's Democratic Republic | 1990 - 2019 | 0.06 (-0.02 to 0.13) | 0.146 |
| Lao People's Democratic Republic | 1998 - 2000 | 1.54 (0.88 to 2.2) | 0 |
| Lao People's Democratic Republic | 2001 - 2003 | -0.67 (-0.98 to -0.35) | 0.001 |
| Lao People's Democratic Republic | 2005 - 2007 | -2.02 (-2.33 to -1.71) | 0 |
| Lao People's Democratic Republic | 2009 - 2010 | -3.36 (-3.99 to -2.73) | 0 |
| Lao People's Democratic Republic | 2012 - 2015 | -1.56 (-1.64 to -1.47) | 0 |
| Latvia | 1990 - 2019 | 17.17 (11.92 to 22.65) | 0 |
| Latvia | 1994 - 1995 | -14.11 (-18.12 to -9.91) | 0 |
| Latvia | 1999 - 2000 | -2.26 (-4.94 to 0.5) | 0.101 |
| Latvia | 2007 - 2007 | -11.04 (-16.65 to -5.04) | 0.002 |
| Latvia | 2012 - 2010 | -2.12 (-5.88 to 1.8) | 0.265 |
| Lebanon | 1990 - 2014 | -2.46 (-3.1 to -1.82) | 0 |
| Lebanon | 1993 - 2019 | -4.82 (-5 to -4.64) | 0 |
| Lebanon | 2001 - 1992 | -2.79 (-4.22 to -1.34) | 0.001 |
| Lebanon | 2004 - 1998 | -0.92 (-1.23 to -0.6) | 0 |
| Lebanon | 2010 - 2002 | -0.01 (-0.16 to 0.13) | 0.85 |
| Lesotho | 1990 - 2006 | -4.61 (-5.66 to -3.56) | 0 |
| Lesotho | 1997 - 2013 | 15.51 (13.88 to 17.16) | 0 |
| Lesotho | 2004 - 2019 | 3.31 (2.58 to 4.05) | 0 |
| Lesotho | 2014 - 1994 | -3.78 (-5.59 to -1.94) | 0 |
| Liberia | 1990 - 2002 | 1.35 (1.01 to 1.69) | 0 |
| Liberia | 1996 - 2013 | -2.64 (-3.06 to -2.22) | 0 |
| Liberia | 2002 - 2019 | 1.09 (0.91 to 1.27) | 0 |
| Liberia | 2012 - 1995 | -1.19 (-1.47 to -0.9) | 0 |
| Libya | 1990 - 2002 | -1.12 (-1.76 to -0.49) | 0.002 |
| Libya | 2000 - 2007 | -5.28 (-7.43 to -3.09) | 0 |
| Libya | 2005 - 2010 | 7.73 (4.09 to 11.5) | 0 |
| Libya | 2009 - 2016 | -3.8 (-10.68 to 3.61) | 0.285 |
| Libya | 2012 - 2019 | 0.7 (-0.48 to 1.9) | 0.229 |
| Lithuania | 1990 - 1999 | 10.99 (7.74 to 14.34) | 0 |
| Lithuania | 1994 - 2005 | -13.81 (-16.43 to -11.12) | 0 |
| Lithuania | 1999 - 2019 | -1.88 (-5.2 to 1.56) | 0.255 |
| Lithuania | 2004 - 2000 | 8.8 (-2.27 to 21.11) | 0.113 |
| Lithuania | 2007 - 2003 | -9.66 (-19.2 to 1.02) | 0.071 |
| Lithuania | 2010 - 2007 | -3.78 (-5.02 to -2.51) | 0 |
| Luxembourg | 1990 - 2010 | -3.73 (-4.54 to -2.92) | 0 |
| Luxembourg | 1994 - 2015 | -0.29 (-3.27 to 2.78) | 0.842 |
| Luxembourg | 1997 - 2019 | -6.21 (-6.66 to -5.76) | 0 |
| Luxembourg | 2004 - 1995 | -5.31 (-5.5 to -5.11) | 0 |
| Luxembourg | 2016 - 2000 | -1.83 (-3.46 to -0.18) | 0.032 |
| Madagascar | 1990 - 2007 | 6.82 (5.04 to 8.62) | 0 |
| Madagascar | 1993 - 2010 | -3.25 (-6.37 to -0.03) | 0.048 |
| Madagascar | 1996 - 2014 | 0.63 (0.03 to 1.23) | 0.04 |
| Madagascar | 2003 - 2019 | -0.88 (-1.03 to -0.72) | 0 |
| Malawi | 1990 - 1992 | -0.06 (-0.34 to 0.22) | 0.646 |
| Malawi | 1999 - 1998 | -4.85 (-5.35 to -4.35) | 0 |
| Malawi | 2006 - 2002 | -2.38 (-2.8 to -1.96) | 0 |
| Malawi | 2014 - 2006 | -0.2 (-0.97 to 0.56) | 0.585 |
| Malaysia | 1990 - 2013 | -0.9 (-1.27 to -0.53) | 0 |
| Malaysia | 2004 - 2019 | 1.72 (0.57 to 2.88) | 0.005 |
| Malaysia | 2010 - 1994 | -0.85 (-1.59 to -0.11) | 0.027 |
| Maldives | 1990 - 2002 | -4.47 (-5.08 to -3.84) | 0 |
| Maldives | 1994 - 2013 | -6.65 (-6.94 to -6.37) | 0 |
| Maldives | 2001 - 2019 | -11.51 (-13 to -9.99) | 0 |
| Maldives | 2004 - 1995 | -8.93 (-10.46 to -7.37) | 0 |
| Maldives | 2007 - 2002 | -5.48 (-5.7 to -5.27) | 0 |
| Maldives | 2015 - 2007 | -0.82 (-1.42 to -0.22) | 0.012 |
| Mali | 1990 - 2010 | -0.99 (-2 to 0.03) | 0.056 |
| Mali | 1993 - 2016 | 0.79 (-0.24 to 1.82) | 0.122 |
| Mali | 1997 - 2019 | -1.97 (-2.23 to -1.71) | 0 |
| Mali | 2005 - 1999 | -0.9 (-1.17 to -0.63) | 0 |
| Mali | 2013 - 2005 | 0.2 (-0.87 to 1.29) | 0.694 |
| Mali | 2017 - 2019 | -1.29 (-3.41 to 0.88) | 0.221 |
| Malta | 1990 - 2000 | -6.07 (-6.59 to -5.55) | 0 |
| Malta | 1995 - 2003 | 0.16 (-1.08 to 1.41) | 0.792 |
| Malta | 1999 - 2007 | -2.42 (-2.96 to -1.89) | 0 |
| Malta | 2005 - 2010 | -3.57 (-3.85 to -3.29) | 0 |
| Malta | 2014 - 2015 | -1.22 (-1.84 to -0.6) | 0.001 |
| Marshall Islands | 1990 - 2019 | 5.37 (4.7 to 6.04) | 0 |
| Marshall Islands | 1992 - 1995 | 4.19 (3.85 to 4.53) | 0 |
| Marshall Islands | 1996 - 2000 | 2.11 (1.95 to 2.27) | 0 |
| Marshall Islands | 2002 - 2007 | 1.58 (1.22 to 1.93) | 0 |
| Marshall Islands | 2006 - 2010 | -0.19 (-0.88 to 0.5) | 0.561 |
| Marshall Islands | 2009 - 2014 | -1.02 (-1.07 to -0.96) | 0 |
| Mauritania | 1990 - 2019 | -1.76 (-2.04 to -1.48) | 0 |
| Mauritania | 1998 - 1992 | -1.37 (-1.49 to -1.25) | 0 |
| Mauritania | 2013 - 1998 | -2.03 (-2.5 to -1.55) | 0 |
| Mauritius | 1990 - 2002 | -1.8 (-2.74 to -0.85) | 0.001 |
| Mauritius | 2000 - 2006 | -7.4 (-8.42 to -6.37) | 0 |
| Mauritius | 2011 - 2013 | -1.03 (-3.18 to 1.17) | 0.34 |
| Mexico | 1990 - 2019 | -1.7 (-2.01 to -1.39) | 0 |
| Mexico | 2006 - 1994 | 0.99 (0.4 to 1.59) | 0.002 |
| Micronesia (Federated States of) | 1990 - 2002 | 0.46 (-0.19 to 1.12) | 0.154 |
| Micronesia (Federated States of) | 1992 - 2013 | 0.07 (-0.02 to 0.15) | 0.117 |
| Micronesia (Federated States of) | 2000 - 2019 | 0.55 (0.49 to 0.6) | 0 |
| Micronesia (Federated States of) | 2010 - 1995 | -0.24 (-0.58 to 0.11) | 0.163 |
| Micronesia (Federated States of) | 2014 - 2002 | -1.41 (-1.6 to -1.22) | 0 |
| Monaco | 1990 - 2007 | -1.65 (-2.05 to -1.26) | 0 |
| Monaco | 1993 - 2010 | -2.81 (-3.6 to -2.01) | 0 |
| Monaco | 1996 - 2016 | -6.01 (-6.26 to -5.75) | 0 |
| Monaco | 2001 - 2019 | -0.53 (-0.68 to -0.39) | 0 |
| Monaco | 2008 - 1999 | 0.34 (-0.08 to 0.77) | 0.104 |
| Monaco | 2012 - 2005 | -2.21 (-2.33 to -2.1) | 0 |
| Mongolia | 1990 - 2019 | 4.26 (1.93 to 6.64) | 0.002 |
| Mongolia | 1993 - 2000 | 1.87 (0.49 to 3.26) | 0.012 |
| Mongolia | 1998 - 2003 | -6.15 (-6.53 to -5.77) | 0 |
| Mongolia | 2008 - 2007 | -2.73 (-4.84 to -0.58) | 0.017 |
| Mongolia | 2012 - 2010 | -6.25 (-10.6 to -1.69) | 0.012 |
| Mongolia | 2015 - 2015 | -0.81 (-2.48 to 0.89) | 0.321 |
| Montenegro | 1990 - 2019 | -1.93 (-6.85 to 3.25) | 0.435 |
| Montenegro | 1992 - 1995 | 4.71 (3.88 to 5.55) | 0 |
| Montenegro | 1999 - 2000 | -1.41 (-2.75 to -0.06) | 0.042 |
| Montenegro | 2004 - 2007 | -5.4 (-5.95 to -4.84) | 0 |
| Montenegro | 2012 - 2010 | -0.79 (-1.47 to -0.1) | 0.028 |
| Morocco | 1990 - 2014 | 1.6 (1.2 to 1.99) | 0 |
| Morocco | 1994 - 2019 | -0.24 (-1.46 to 1) | 0.687 |
| Morocco | 1997 - 1992 | -2.13 (-3.37 to -0.87) | 0.003 |
| Morocco | 2000 - 1998 | -0.76 (-0.93 to -0.58) | 0 |
| Morocco | 2008 - 2002 | -2.15 (-2.25 to -2.05) | 0 |
| Mozambique | 1990 - 2006 | -2.44 (-2.58 to -2.3) | 0 |
| Mozambique | 2002 - 2013 | 1.87 (0.62 to 3.14) | 0.006 |
| Mozambique | 2006 - 2019 | 4.84 (3.53 to 6.17) | 0 |
| Mozambique | 2010 - 1994 | 1.91 (0.62 to 3.22) | 0.006 |
| Mozambique | 2014 - 2002 | -1.18 (-1.78 to -0.57) | 0.001 |
| Myanmar | 1990 - 2013 | -1.67 (-2.17 to -1.17) | 0 |
| Myanmar | 1999 - 2019 | -6.33 (-6.66 to -6) | 0 |
| Myanmar | 2011 - 1995 | -4.88 (-5.38 to -4.37) | 0 |
| Namibia | 1990 - 2002 | -0.02 (-0.37 to 0.33) | 0.911 |
| Namibia | 1997 - 2007 | 6.46 (3.66 to 9.33) | 0 |
| Namibia | 2000 - 2010 | -1.49 (-3.88 to 0.96) | 0.21 |
| Namibia | 2003 - 2016 | -7.09 (-9.55 to -4.56) | 0 |
| Namibia | 2006 - 2019 | -4.64 (-5.43 to -3.84) | 0 |
| Namibia | 2011 - 1999 | -0.29 (-0.59 to 0.01) | 0.058 |
| Nauru | 1990 - 2005 | 2.21 (1.54 to 2.89) | 0 |
| Nauru | 1993 - 2019 | 4.66 (4.23 to 5.09) | 0 |
| Nauru | 1998 - 2000 | 3.24 (2.57 to 3.91) | 0 |
| Nauru | 2002 - 2003 | 0.32 (0.09 to 0.55) | 0.009 |
| Nauru | 2009 - 2007 | -2.67 (-2.85 to -2.5) | 0 |
| Nauru | 2017 - 2010 | -1.21 (-2.57 to 0.17) | 0.081 |
| Nepal | 1990 - 2015 | -0.89 (-1.72 to -0.05) | 0.04 |
| Nepal | 1993 - 2019 | -4.16 (-4.36 to -3.95) | 0 |
| Nepal | 2001 - 1995 | -2.04 (-3.47 to -0.59) | 0.01 |
| Nepal | 2004 - 2000 | -0.98 (-1.3 to -0.66) | 0 |
| Nepal | 2010 - 2007 | -1.47 (-1.81 to -1.12) | 0 |
| Nepal | 2016 - 2010 | 0.05 (-0.81 to 0.91) | 0.911 |
| Netherlands | 1990 - 2014 | 0.46 (0.12 to 0.8) | 0.011 |
| Netherlands | 2000 - 2019 | -3.38 (-7.32 to 0.73) | 0.099 |
| Netherlands | 2003 - 1992 | -11.62 (-13.71 to -9.48) | 0 |
| Netherlands | 2007 - 1998 | -7.25 (-7.9 to -6.59) | 0 |
| Netherlands | 2015 - 2002 | 1.62 (-0.24 to 3.52) | 0.084 |
| New Zealand | 1990 - 2006 | -7.9 (-14.54 to -0.76) | 0.032 |
| New Zealand | 1992 - 2013 | -3.96 (-4.15 to -3.77) | 0 |
| New Zealand | 2014 - 2019 | -1.61 (-3.62 to 0.45) | 0.119 |
| Nicaragua | 1990 - 1994 | -4.18 (-5.32 to -3.02) | 0 |
| Nicaragua | 2000 - 2002 | 0.83 (0.42 to 1.24) | 0 |
| Niger | 1990 - 2013 | 0.8 (0.44 to 1.16) | 0 |
| Niger | 1995 - 2019 | -0.74 (-1.01 to -0.47) | 0 |
| Niger | 2002 - 1995 | -2.58 (-2.78 to -2.38) | 0 |
| Niger | 2010 - 2002 | -0.56 (-2.14 to 1.05) | 0.465 |
| Niger | 2013 - 2007 | 2.91 (1.28 to 4.55) | 0.002 |
| Niger | 2016 - 2010 | 0.03 (-0.79 to 0.85) | 0.942 |
| Nigeria | 1990 - 2016 | 0.62 (0.13 to 1.1) | 0.016 |
| Nigeria | 1995 - 2019 | 2.59 (1.4 to 3.79) | 0 |
| Nigeria | 1999 - 1999 | -1.66 (-3.97 to 0.7) | 0.151 |
| Nigeria | 2002 - 2005 | -5.33 (-5.75 to -4.92) | 0 |
| Nigeria | 2008 - 2019 | -0.44 (-0.7 to -0.19) | 0.002 |
| Nigeria | 2016 - 2000 | -2.09 (-3.02 to -1.15) | 0 |
| Niue | 1990 - 2003 | 4.88 (3.45 to 6.32) | 0 |
| Niue | 1993 - 2007 | -0.98 (-1.28 to -0.69) | 0 |
| Niue | 2002 - 2010 | 0.63 (0.01 to 1.25) | 0.048 |
| Niue | 2008 - 2015 | -1.2 (-1.4 to -0.99) | 0 |
| North Macedonia | 1990 - 2019 | -0.19 (-2.86 to 2.56) | 0.884 |
| North Macedonia | 1993 - 1995 | 3.88 (2.35 to 5.43) | 0 |
| North Macedonia | 1998 - 2000 | -6.66 (-8.77 to -4.51) | 0 |
| North Macedonia | 2002 - 2007 | -2.93 (-4.36 to -1.47) | 0.001 |
| North Macedonia | 2007 - 2010 | -7.07 (-11.61 to -2.3) | 0.007 |
| North Macedonia | 2010 - 2014 | -1.51 (-2.15 to -0.87) | 0 |
| Northern Mariana Islands | 1990 - 2019 | -1.63 (-1.85 to -1.41) | 0 |
| Northern Mariana Islands | 1998 - 1992 | 2.68 (1.75 to 3.61) | 0 |
| Northern Mariana Islands | 2002 - 1998 | 0.32 (0.03 to 0.6) | 0.034 |
| Northern Mariana Islands | 2009 - 2002 | -1.07 (-2.69 to 0.59) | 0.186 |
| Northern Mariana Islands | 2012 - 2006 | 0.68 (-0.19 to 1.56) | 0.115 |
| Northern Mariana Islands | 2016 - 2013 | -0.94 (-1.85 to -0.02) | 0.045 |
| Norway | 1990 - 2019 | -4.96 (-5.49 to -4.43) | 0 |
| Norway | 1996 - 1994 | -2.42 (-5.68 to 0.96) | 0.149 |
| Norway | 1999 - 2002 | -6.02 (-6.19 to -5.86) | 0 |
| Norway | 2016 - 2013 | -1.23 (-4.27 to 1.9) | 0.417 |
| Oman | 1990 - 2019 | -0.81 (-1.02 to -0.59) | 0 |
| Oman | 2006 - 1995 | -6.04 (-6.44 to -5.65) | 0 |
| Oman | 2016 - 2002 | -2.45 (-5.27 to 0.46) | 0.094 |
| Pakistan | 1990 - 2007 | 5.13 (4.86 to 5.39) | 0 |
| Pakistan | 1995 - 2010 | 0.61 (0.46 to 0.76) | 0 |
| Pakistan | 2003 - 2016 | -1.11 (-1.35 to -0.86) | 0 |
| Pakistan | 2009 - 2019 | -0.06 (-0.46 to 0.35) | 0.767 |
| Pakistan | 2014 - 1999 | -1.19 (-1.5 to -0.88) | 0 |
| Palau | 1990 - 2005 | -0.32 (-0.4 to -0.24) | 0 |
| Palau | 1999 - 2019 | -1.84 (-2.23 to -1.45) | 0 |
| Palau | 2003 - 2000 | 1.44 (0.65 to 2.24) | 0.001 |
| Palau | 2006 - 2003 | 0.49 (0.25 to 0.74) | 0.001 |
| Palau | 2011 - 2007 | -0.73 (-0.82 to -0.65) | 0 |
| Palestine | 1990 - 2010 | -1.51 (-1.8 to -1.2) | 0 |
| Palestine | 1998 - 2015 | -3.95 (-4.06 to -3.84) | 0 |
| Palestine | 2011 - 2019 | -5.47 (-7.11 to -3.81) | 0 |
| Palestine | 2014 - 1995 | 5.13 (3.17 to 7.13) | 0 |
| Palestine | 2017 - 2000 | -0.08 (-2.33 to 2.22) | 0.942 |
| Panama | 1990 - 2007 | -7.19 (-9.84 to -4.47) | 0 |
| Panama | 1994 - 2010 | 0.44 (-9.06 to 10.93) | 0.926 |
| Panama | 1997 - 2014 | -12.06 (-20.21 to -3.07) | 0.013 |
| Panama | 2000 - 2019 | 2.29 (1.32 to 3.28) | 0 |
| Panama | 2009 - 1992 | -3.13 (-3.99 to -2.26) | 0 |
| Papua New Guinea | 1990 - 1998 | 0.46 (0.1 to 0.82) | 0.015 |
| Papua New Guinea | 1997 - 2002 | 2.66 (2.39 to 2.94) | 0 |
| Papua New Guinea | 2006 - 2006 | 0.47 (-0.26 to 1.21) | 0.195 |
| Papua New Guinea | 2011 - 2013 | -0.55 (-0.8 to -0.31) | 0 |
| Paraguay | 1990 - 2019 | 0.16 (-1.48 to 1.83) | 0.833 |
| Paraguay | 1995 - 1994 | -8.98 (-14.96 to -2.59) | 0.01 |
| Paraguay | 1998 - 2002 | -2.46 (-5.69 to 0.88) | 0.134 |
| Paraguay | 2002 - 2013 | -0.63 (-1.75 to 0.51) | 0.252 |
| Paraguay | 2009 - 2019 | -4.47 (-10.64 to 2.12) | 0.162 |
| Paraguay | 2012 - 1995 | -0.11 (-1.32 to 1.11) | 0.842 |
| Peru | 1990 - 2002 | -1.26 (-3.53 to 1.07) | 0.267 |
| Peru | 1995 - 2007 | -9.88 (-14.12 to -5.42) | 0 |
| Peru | 1999 - 2010 | -3.87 (-5.1 to -2.62) | 0 |
| Peru | 2007 - 2016 | 3 (-6.77 to 13.78) | 0.539 |
| Peru | 2010 - 2019 | -4.14 (-5.29 to -2.98) | 0 |
| Philippines | 1990 - 1999 | -0.94 (-2.14 to 0.29) | 0.122 |
| Philippines | 1994 - 2005 | 7.86 (4.16 to 11.68) | 0 |
| Philippines | 1997 - 2019 | 20.51 (18.73 to 22.31) | 0 |
| Philippines | 2001 - 2000 | 5.63 (4.16 to 7.13) | 0 |
| Philippines | 2005 - 2003 | 1.7 (1.22 to 2.19) | 0 |
| Philippines | 2012 - 2007 | -0.94 (-1.55 to -0.32) | 0.006 |
| Poland | 1990 - 2010 | -1.89 (-6.6 to 3.06) | 0.417 |
| Poland | 1992 - 2015 | -6.27 (-7.05 to -5.49) | 0 |
| Poland | 1999 - 2019 | -11.32 (-15.83 to -6.57) | 0 |
| Poland | 2002 - 1995 | -2.97 (-4.64 to -1.28) | 0.002 |
| Poland | 2007 - 2000 | -7.05 (-8.36 to -5.73) | 0 |
| Poland | 2013 - 2007 | -3.08 (-5.65 to -0.43) | 0.026 |
| Portugal | 1990 - 2010 | -2.91 (-3.22 to -2.61) | 0 |
| Portugal | 2002 - 2014 | -9.91 (-15.2 to -4.29) | 0.002 |
| Portugal | 2005 - 2019 | -5.21 (-5.88 to -4.53) | 0 |
| Portugal | 2013 - 1992 | -1.33 (-2.38 to -0.26) | 0.017 |
| Puerto Rico | 1990 - 1998 | -3.58 (-3.79 to -3.36) | 0 |
| Qatar | 1990 - 2002 | 1.73 (-1 to 4.54) | 0.197 |
| Qatar | 1995 - 2006 | -13.39 (-18.54 to -7.92) | 0 |
| Qatar | 1999 - 2013 | -6.19 (-9.72 to -2.52) | 0.003 |
| Qatar | 2004 - 2019 | 0.3 (-10.28 to 12.13) | 0.954 |
| Qatar | 2007 - 1994 | -9.47 (-14.56 to -4.08) | 0.003 |
| Qatar | 2011 - 2002 | -2.05 (-3.43 to -0.66) | 0.007 |
| Republic of Korea | 1990 - 2013 | -15.56 (-16.2 to -14.92) | 0 |
| Republic of Korea | 1995 - 2019 | -13.03 (-13.94 to -12.11) | 0 |
| Republic of Korea | 2000 - 1995 | -7.82 (-8.52 to -7.11) | 0 |
| Republic of Korea | 2006 - 2002 | -6.2 (-6.82 to -5.59) | 0 |
| Republic of Korea | 2013 - 2007 | -1.67 (-2.5 to -0.83) | 0.001 |
| Republic of Moldova | 1990 - 2010 | 8.26 (3.99 to 12.71) | 0.001 |
| Republic of Moldova | 1995 - 2016 | -6.82 (-14.6 to 1.68) | 0.107 |
| Republic of Moldova | 1999 - 2019 | -0.48 (-2.74 to 1.82) | 0.663 |
| Republic of Moldova | 2007 - 1999 | -4.38 (-5.55 to -3.2) | 0 |
| Romania | 1990 - 2005 | 5.27 (4.28 to 6.28) | 0 |
| Romania | 1996 - 2019 | -4.09 (-4.36 to -3.81) | 0 |
| Romania | 2010 - 2000 | -7.75 (-13.2 to -1.97) | 0.012 |
| Romania | 2013 - 2003 | -1.03 (-2.48 to 0.44) | 0.159 |
| Russian Federation | 1990 - 2007 | 22.67 (18.28 to 27.22) | 0 |
| Russian Federation | 1994 - 2010 | -13.06 (-21.42 to -3.8) | 0.009 |
| Russian Federation | 1997 - 2015 | 6.34 (4.16 to 8.56) | 0 |
| Russian Federation | 2003 - 2019 | -5.28 (-5.95 to -4.61) | 0 |
| Rwanda | 1990 - 1995 | 2.59 (2.1 to 3.08) | 0 |
| Rwanda | 1995 - 2000 | -0.87 (-2.97 to 1.27) | 0.399 |
| Rwanda | 1998 - 2007 | -6.82 (-7.03 to -6.61) | 0 |
| Rwanda | 2007 - 2010 | -3.82 (-5.78 to -1.82) | 0.001 |
| Rwanda | 2010 - 2014 | 0.15 (-0.05 to 0.34) | 0.136 |
| Saint Kitts and Nevis | 1990 - 2019 | -2.66 (-4.31 to -0.98) | 0.004 |
| Saint Kitts and Nevis | 1992 - 1992 | -7.69 (-8.06 to -7.32) | 0 |
| Saint Kitts and Nevis | 1998 - 1998 | -9.42 (-9.98 to -8.86) | 0 |
| Saint Kitts and Nevis | 2003 - 2002 | -6.32 (-7 to -5.64) | 0 |
| Saint Kitts and Nevis | 2008 - 2006 | -3.41 (-3.65 to -3.17) | 0 |
| Saint Lucia | 1990 - 2013 | -6.4 (-6.71 to -6.09) | 0 |
| Saint Lucia | 2004 - 2019 | 0.53 (-0.75 to 1.82) | 0.402 |
| Saint Lucia | 2011 - 1994 | -2.52 (-3.37 to -1.66) | 0 |
| Saint Vincent and the Grenadines | 1990 - 2002 | 0.92 (-0.13 to 1.98) | 0.083 |
| Saint Vincent and the Grenadines | 1995 - 2013 | -5.24 (-5.98 to -4.49) | 0 |
| Saint Vincent and the Grenadines | 2002 - 2019 | -2.45 (-2.96 to -1.95) | 0 |
| Saint Vincent and the Grenadines | 2011 - 1995 | -0.28 (-0.91 to 0.35) | 0.363 |
| Samoa | 1990 - 2002 | 1.46 (1.06 to 1.86) | 0 |
| Samoa | 1992 - 2007 | 1.02 (0.81 to 1.22) | 0 |
| Samoa | 1996 - 2010 | 1.61 (1.48 to 1.74) | 0 |
| Samoa | 2001 - 2016 | 0.97 (0.84 to 1.09) | 0 |
| Samoa | 2006 - 2019 | 1.73 (1.6 to 1.85) | 0 |
| Samoa | 2011 - 1999 | -0.34 (-0.38 to -0.29) | 0 |
| San Marino | 1990 - 2005 | -1.18 (-1.68 to -0.67) | 0 |
| San Marino | 1993 - 2019 | -2.82 (-3.3 to -2.34) | 0 |
| San Marino | 1997 - 2000 | -0.55 (-1.54 to 0.44) | 0.249 |
| San Marino | 2000 - 2003 | 1.6 (1.29 to 1.92) | 0 |
| San Marino | 2005 - 2007 | -2.34 (-2.67 to -2.01) | 0 |
| San Marino | 2010 - 2010 | -0.21 (-0.33 to -0.1) | 0.001 |
| Sao Tome and Principe | 1990 - 2015 | 5.07 (4.5 to 5.64) | 0 |
| Sao Tome and Principe | 2000 - 2019 | -2.2 (-2.64 to -1.75) | 0 |
| Sao Tome and Principe | 2012 - 1995 | -0.16 (-1.14 to 0.83) | 0.746 |
| Saudi Arabia | 1990 - 2000 | 0.26 (0.18 to 0.33) | 0 |
| Saudi Arabia | 2003 - 2007 | 6.25 (5.23 to 7.27) | 0 |
| Saudi Arabia | 2006 - 2010 | -1.2 (-2.13 to -0.25) | 0.016 |
| Saudi Arabia | 2009 - 2014 | -4.39 (-4.91 to -3.87) | 0 |
| Saudi Arabia | 2013 - 2019 | -1.5 (-1.75 to -1.26) | 0 |
| Senegal | 1990 - 1992 | 0.07 (-0.51 to 0.66) | 0.805 |
| Senegal | 2002 - 1998 | -1.65 (-2.54 to -0.76) | 0.001 |
| Senegal | 2012 - 2002 | 0.07 (-1.34 to 1.51) | 0.916 |
| Serbia | 1990 - 2006 | 3.12 (0.85 to 5.45) | 0.009 |
| Serbia | 1996 - 2013 | -4.82 (-5.15 to -4.5) | 0 |
| Serbia | 2013 - 2019 | -0.54 (-2.68 to 1.64) | 0.608 |
| Seychelles | 1990 - 1994 | -3.15 (-3.82 to -2.48) | 0 |
| Seychelles | 1995 - 2002 | -1.26 (-1.58 to -0.94) | 0 |
| Seychelles | 2004 - 2013 | 0.96 (-1.91 to 3.91) | 0.493 |
| Seychelles | 2007 - 2019 | -3.01 (-3.5 to -2.52) | 0 |
| Seychelles | 2014 - 1995 | -1.08 (-1.82 to -0.34) | 0.007 |
| Sierra Leone | 1990 - 2002 | -0.54 (-1.23 to 0.16) | 0.122 |
| Sierra Leone | 1994 - 2007 | 2.99 (2.84 to 3.14) | 0 |
| Sierra Leone | 2006 - 2010 | -1.09 (-1.2 to -0.98) | 0 |
| Singapore | 1990 - 2016 | -2.52 (-3.05 to -1.98) | 0 |
| Singapore | 1999 - 2019 | -8.77 (-14.12 to -3.09) | 0.005 |
| Singapore | 2002 - 1999 | -1.74 (-3.61 to 0.18) | 0.072 |
| Singapore | 2007 - 2005 | -5.48 (-6.52 to -4.44) | 0 |
| Singapore | 2014 - 2019 | -2.69 (-4.3 to -1.06) | 0.003 |
| Slovakia | 1990 - 2000 | -5.83 (-7.41 to -4.22) | 0 |
| Slovakia | 1995 - 2003 | -0.51 (-6.79 to 6.18) | 0.867 |
| Slovakia | 1998 - 2007 | -5.78 (-8.85 to -2.61) | 0.002 |
| Slovakia | 2002 - 2010 | -1.75 (-3.96 to 0.51) | 0.118 |
| Slovakia | 2007 - 2015 | -7.26 (-9.58 to -4.88) | 0 |
| Slovakia | 2012 - 2019 | -1.03 (-2.63 to 0.6) | 0.195 |
| Slovenia | 1990 - 1995 | -1.42 (-2.87 to 0.04) | 0.057 |
| Slovenia | 1999 - 2000 | -9.74 (-10.91 to -8.55) | 0 |
| Slovenia | 2008 - 2007 | -4.21 (-5.23 to -3.18) | 0 |
| Solomon Islands | 1990 - 2010 | -0.33 (-0.68 to 0.02) | 0.06 |
| Solomon Islands | 1996 - 2014 | 0.8 (0.53 to 1.06) | 0 |
| Solomon Islands | 2004 - 2019 | -0.02 (-0.19 to 0.15) | 0.816 |
| Solomon Islands | 2014 - 1992 | -1.4 (-1.81 to -0.98) | 0 |
| Somalia | 1990 - 1998 | -0.11 (-0.53 to 0.31) | 0.598 |
| Somalia | 1994 - 2002 | 2.01 (1.89 to 2.13) | 0 |
| Somalia | 2004 - 2006 | 1.19 (0.79 to 1.58) | 0 |
| Somalia | 2009 - 2013 | -0.16 (-0.25 to -0.07) | 0.002 |
| South Africa | 1990 - 2019 | 2.13 (-7.31 to 12.52) | 0.655 |
| South Africa | 1992 - 1994 | -9.87 (-13.86 to -5.69) | 0 |
| South Africa | 1996 - 2002 | 5.02 (2.08 to 8.05) | 0.002 |
| South Africa | 2003 - 2013 | -5.95 (-6.85 to -5.04) | 0 |
| South Sudan | 1990 - 2019 | -0.08 (-0.32 to 0.17) | 0.524 |
| South Sudan | 2002 - 1995 | 3.37 (1.22 to 5.57) | 0.003 |
| South Sudan | 2006 - 2002 | -0.09 (-0.31 to 0.14) | 0.433 |
| Spain | 1990 - 2007 | -4.38 (-6.09 to -2.64) | 0 |
| Spain | 1993 - 2010 | -1.3 (-1.61 to -0.99) | 0 |
| Spain | 2003 - 2016 | -3.22 (-4.95 to -1.46) | 0.002 |
| Spain | 2007 - 2019 | -5.86 (-9.11 to -2.5) | 0.003 |
| Spain | 2010 - 1999 | -4.09 (-4.89 to -3.29) | 0 |
| Spain | 2016 - 2005 | 0.52 (-1.9 to 2.99) | 0.656 |
| Sri Lanka | 1990 - 2019 | 0.54 (-0.98 to 2.09) | 0.471 |
| Sri Lanka | 1997 - 2000 | -9.68 (-12.18 to -7.12) | 0 |
| Sri Lanka | 2002 - 2003 | -1.45 (-1.91 to -0.98) | 0 |
| Sudan | 1990 - 2007 | -1.91 (-2.53 to -1.3) | 0 |
| Sudan | 1992 - 2010 | -0.89 (-1.53 to -0.25) | 0.01 |
| Sudan | 1995 - 2015 | -1.42 (-2.09 to -0.75) | 0.001 |
| Sudan | 1998 - 2019 | -3.33 (-3.5 to -3.17) | 0 |
| Sudan | 2004 - 1995 | -2.5 (-2.89 to -2.11) | 0 |
| Sudan | 2008 - 2000 | -1.57 (-1.62 to -1.51) | 0 |
| Suriname | 1990 - 2007 | 4.24 (-0.35 to 9.05) | 0.068 |
| Suriname | 1993 - 2010 | -7.77 (-16.27 to 1.6) | 0.094 |
| Suriname | 1996 - 2014 | -0.61 (-2.58 to 1.4) | 0.519 |
| Suriname | 2002 - 2019 | -7.36 (-14.65 to 0.55) | 0.065 |
| Suriname | 2005 - 1992 | -2.1 (-3.22 to -0.97) | 0.002 |
| Suriname | 2013 - 1998 | 2.83 (0.97 to 4.73) | 0.006 |
| Sweden | 1990 - 2002 | -4.54 (-5.49 to -3.57) | 0 |
| Sweden | 1996 - 2006 | 0.01 (-6.01 to 6.42) | 0.997 |
| Sweden | 1999 - 2013 | -4.87 (-5.13 to -4.61) | 0 |
| Sweden | 2016 - 2019 | -1.23 (-5.65 to 3.39) | 0.577 |
| Switzerland | 1990 - 1994 | -0.58 (-2.26 to 1.14) | 0.476 |
| Switzerland | 1994 - 2002 | -4.5 (-4.91 to -4.1) | 0 |
| Switzerland | 2005 - 2013 | -7.82 (-12.87 to -2.48) | 0.008 |
| Switzerland | 2008 - 2019 | -3.66 (-8.88 to 1.87) | 0.173 |
| Switzerland | 2011 - 1995 | -8.97 (-14.32 to -3.28) | 0.005 |
| Switzerland | 2014 - 2002 | -0.44 (-2.02 to 1.16) | 0.558 |
| Syrian Arab Republic | 1990 - 2007 | -4.29 (-5.2 to -3.38) | 0 |
| Syrian Arab Republic | 1997 - 2010 | -1.86 (-2.76 to -0.94) | 0 |
| Syrian Arab Republic | 2004 - 2016 | -3.96 (-5.49 to -2.39) | 0 |
| Syrian Arab Republic | 2009 - 2019 | 0.61 (0.05 to 1.17) | 0.034 |
| Taiwan (Province of China) | 1990 - 1999 | -4.37 (-4.72 to -4.02) | 0 |
| Taiwan (Province of China) | 2000 - 2005 | -0.29 (-1.83 to 1.28) | 0.701 |
| Taiwan (Province of China) | 2005 - 2019 | -3.29 (-5.65 to -0.86) | 0.011 |
| Taiwan (Province of China) | 2009 - 2000 | -1.25 (-1.73 to -0.77) | 0 |
| Tajikistan | 1990 - 2003 | 8.51 (6.91 to 10.13) | 0 |
| Tajikistan | 1995 - 2007 | -6.85 (-7.54 to -6.15) | 0 |
| Tajikistan | 2004 - 2010 | 1.34 (0.95 to 1.73) | 0 |
| Thailand | 1990 - 2015 | 1.19 (0.54 to 1.85) | 0.001 |
| Thailand | 2000 - 2019 | -6.27 (-6.59 to -5.95) | 0 |
| Thailand | 2015 - 1995 | 0.65 (-2.32 to 3.71) | 0.658 |
| Timor-Leste | 1990 - 2000 | 0.37 (-0.86 to 1.62) | 0.527 |
| Timor-Leste | 1994 - 2007 | -1.89 (-5.9 to 2.29) | 0.341 |
| Timor-Leste | 1997 - 2010 | 1.41 (0.35 to 2.48) | 0.013 |
| Timor-Leste | 2003 - 2014 | -3.46 (-4.96 to -1.94) | 0 |
| Timor-Leste | 2008 - 2019 | 5.23 (4.04 to 6.44) | 0 |
| Timor-Leste | 2014 - 1992 | 1.73 (0.64 to 2.83) | 0.004 |
| Togo | 1990 - 1998 | -0.58 (-0.75 to -0.4) | 0 |
| Togo | 2000 - 2002 | -1.16 (-1.69 to -0.62) | 0 |
| Togo | 2006 - 2006 | 2.11 (-0.38 to 4.66) | 0.09 |
| Togo | 2009 - 2013 | -1 (-1.77 to -0.23) | 0.015 |
| Togo | 2014 - 2019 | 0.66 (-1.83 to 3.21) | 0.581 |
| Togo | 2017 - 1994 | -1.19 (-3.66 to 1.34) | 0.326 |
| Tokelau | 1990 - 2002 | 0.77 (0.28 to 1.27) | 0.005 |
| Tokelau | 1992 - 2013 | 0.45 (0.37 to 0.53) | 0 |
| Tokelau | 1999 - 2019 | 0.87 (0.44 to 1.3) | 0.001 |
| Tokelau | 2002 - 1995 | -0.04 (-0.09 to 0.01) | 0.088 |
| Tokelau | 2011 - 2002 | -0.83 (-0.97 to -0.69) | 0 |
| Tokelau | 2016 - 2007 | -0.52 (-0.74 to -0.31) | 0 |
| Tonga | 1990 - 2010 | 0.05 (-0.03 to 0.13) | 0.212 |
| Tonga | 2000 - 2016 | -3.8 (-4.1 to -3.49) | 0 |
| Tonga | 2005 - 2019 | 1.15 (0.9 to 1.39) | 0 |
| Tonga | 2011 - 1999 | -0.15 (-0.29 to -0.01) | 0.035 |
| Trinidad and Tobago | 1990 - 2005 | -0.08 (-1.24 to 1.09) | 0.89 |
| Trinidad and Tobago | 1998 - 2019 | -8.55 (-13.58 to -3.24) | 0.003 |
| Trinidad and Tobago | 2002 - 2000 | -3.32 (-3.94 to -2.69) | 0 |
| Tunisia | 1990 - 2003 | -0.08 (-0.46 to 0.29) | 0.644 |
| Tunisia | 1997 - 2007 | -2.52 (-2.68 to -2.37) | 0 |
| Tunisia | 2012 - 2010 | -1.21 (-1.72 to -0.69) | 0 |
| Turkey | 1990 - 2015 | -2.46 (-3.18 to -1.74) | 0 |
| Turkey | 1998 - 2019 | -6.89 (-7.65 to -6.12) | 0 |
| Turkey | 2006 - 1995 | -4.08 (-5.1 to -3.05) | 0 |
| Turkey | 2012 - 2000 | 0.77 (-3.21 to 4.9) | 0.692 |
| Turkey | 2015 - 2007 | -3.11 (-4.83 to -1.35) | 0.002 |
| Turkmenistan | 1990 - 2010 | 10.19 (8.63 to 11.78) | 0 |
| Turkmenistan | 1996 - 2014 | -4 (-6.3 to -1.64) | 0.003 |
| Turkmenistan | 2001 - 2019 | 0.75 (-1.41 to 2.95) | 0.475 |
| Turkmenistan | 2006 - 1992 | -9.57 (-12.88 to -6.13) | 0 |
| Turkmenistan | 2010 - 1998 | -1.02 (-2.14 to 0.11) | 0.074 |
| Tuvalu | 1990 - 2002 | -0.29 (-0.8 to 0.23) | 0.257 |
| Tuvalu | 1994 - 2006 | 0.31 (0.26 to 0.37) | 0 |
| Tuvalu | 2012 - 2013 | -0.81 (-1.02 to -0.61) | 0 |
| Uganda | 1990 - 2019 | 4.87 (2.78 to 7) | 0 |
| Uganda | 1993 - 1994 | 7.28 (6.76 to 7.81) | 0 |
| Uganda | 2001 - 2002 | -0.7 (-2.41 to 1.04) | 0.397 |
| Uganda | 2005 - 2013 | -6.76 (-7.81 to -5.7) | 0 |
| Uganda | 2010 - 2019 | 1.53 (0.74 to 2.33) | 0.001 |
| Uganda | 2016 - 1995 | -0.2 (-1.96 to 1.59) | 0.812 |
| Ukraine | 1990 - 2002 | 12.09 (7.45 to 16.92) | 0 |
| Ukraine | 1995 - 2007 | -5.38 (-19.75 to 11.55) | 0.486 |
| Ukraine | 1998 - 2010 | 2.45 (0.98 to 3.94) | 0.003 |
| Ukraine | 2008 - 2016 | -14.33 (-25.71 to -1.21) | 0.035 |
| Ukraine | 2011 - 2019 | 5.24 (2.58 to 7.98) | 0.001 |
| United Arab Emirates | 1990 - 1999 | -2.79 (-2.91 to -2.66) | 0 |
| United Arab Emirates | 2003 - 2005 | -7.08 (-9.16 to -4.96) | 0 |
| United Arab Emirates | 2006 - 2019 | -2.27 (-4.46 to -0.02) | 0.049 |
| United Arab Emirates | 2009 - 2000 | 1.68 (-0.85 to 4.27) | 0.176 |
| United Arab Emirates | 2012 - 2003 | 9.74 (6.92 to 12.63) | 0 |
| United Arab Emirates | 2015 - 2007 | -0.55 (-1.4 to 0.31) | 0.188 |
| United Kingdom | 1990 - 2010 | -3.07 (-3.31 to -2.82) | 0 |
| United Kingdom | 1997 - 2015 | -2.2 (-2.83 to -1.56) | 0 |
| United Kingdom | 2002 - 2019 | -4.04 (-4.27 to -3.81) | 0 |
| United Kingdom | 2012 - 1995 | -0.59 (-1.04 to -0.14) | 0.013 |
| United Republic of Tanzania | 1990 - 2000 | -0.9 (-1.51 to -0.29) | 0.007 |
| United Republic of Tanzania | 1995 - 2007 | 4.85 (4 to 5.71) | 0 |
| United Republic of Tanzania | 2000 - 2010 | -2.15 (-4.58 to 0.34) | 0.085 |
| United Republic of Tanzania | 2003 - 2014 | 1.44 (1.19 to 1.68) | 0 |
| United Republic of Tanzania | 2013 - 2019 | -0.57 (-1.04 to -0.1) | 0.022 |
| United States Virgin Islands | 1990 - 1992 | -5.32 (-6.8 to -3.83) | 0 |
| United States Virgin Islands | 1992 - 1998 | -2.37 (-3.84 to -0.87) | 0.005 |
| United States Virgin Islands | 1995 - 2002 | -1.01 (-1.34 to -0.68) | 0 |
| United States Virgin Islands | 2001 - 2006 | -4.15 (-4.31 to -4) | 0 |
| United States Virgin Islands | 2010 - 2013 | -1.41 (-1.79 to -1.03) | 0 |
| United States Virgin Islands | 2016 - 2019 | 0.13 (-0.86 to 1.13) | 0.779 |
| United States of America | 1990 - 1994 | -0.62 (-0.81 to -0.43) | 0 |
| United States of America | 1998 - 2002 | 2.64 (1.55 to 3.73) | 0 |
| United States of America | 2002 - 2013 | -2.26 (-2.46 to -2.05) | 0 |
| United States of America | 2012 - 2019 | -0.05 (-1.39 to 1.3) | 0.934 |
| United States of America | 2016 - 1995 | -2.14 (-3.75 to -0.5) | 0.014 |
| Uruguay | 1990 - 2002 | -2.7 (-4.34 to -1.04) | 0.004 |
| Uruguay | 1994 - 2007 | -5.82 (-10.99 to -0.35) | 0.039 |
| Uruguay | 1997 - 2010 | -2.24 (-3.81 to -0.63) | 0.01 |
| Uruguay | 2002 - 2016 | -5.98 (-8.61 to -3.27) | 0 |
| Uruguay | 2006 - 2019 | -3.75 (-4.78 to -2.71) | 0 |
| Uruguay | 2013 - 1999 | -1.73 (-2.81 to -0.65) | 0.004 |
| Uzbekistan | 1990 - 2005 | 10.77 (9.38 to 12.18) | 0 |
| Uzbekistan | 1995 - 2019 | -1.28 (-2.47 to -0.07) | 0.039 |
| Uzbekistan | 2001 - 2000 | 7.22 (4.48 to 10.03) | 0 |
| Uzbekistan | 2005 - 2003 | 0.12 (-0.53 to 0.78) | 0.694 |
| Uzbekistan | 2013 - 2007 | -2.72 (-3.8 to -1.63) | 0 |
| Vanuatu | 1990 - 2010 | 6.44 (5.59 to 7.3) | 0 |
| Vanuatu | 1994 - 2015 | 0.88 (0.33 to 1.43) | 0.004 |
| Vanuatu | 2000 - 2019 | -3.15 (-5.45 to -0.8) | 0.012 |
| Vanuatu | 2003 - 1995 | 2.58 (1.79 to 3.37) | 0 |
| Vanuatu | 2008 - 2000 | 0.06 (-0.1 to 0.23) | 0.435 |
| Venezuela (Bolivarian Republic of) | 1990 - 2007 | -1.33 (-3.16 to 0.54) | 0.152 |
| Venezuela (Bolivarian Republic of) | 1995 - 2010 | -3.64 (-4.33 to -2.96) | 0 |
| Venezuela (Bolivarian Republic of) | 2005 - 2014 | -0.69 (-2.03 to 0.67) | 0.299 |
| Venezuela (Bolivarian Republic of) | 2012 - 2019 | 2.53 (0.06 to 5.05) | 0.045 |
| Viet Nam | 1990 - 1992 | -3.46 (-3.96 to -2.96) | 0 |
| Viet Nam | 1993 - 1998 | -3.97 (-4.13 to -3.81) | 0 |
| Viet Nam | 2000 - 2002 | -2.13 (-2.28 to -1.98) | 0 |
| Viet Nam | 2007 - 2006 | -1.32 (-1.59 to -1.06) | 0 |
| Viet Nam | 2012 - 2013 | -0.41 (-0.54 to -0.27) | 0 |
| Yemen | 1990 - 2019 | -1.12 (-1.69 to -0.55) | 0 |
| Yemen | 1995 - 1994 | -1.88 (-1.97 to -1.79) | 0 |
| Yemen | 2013 - 2002 | 1.31 (0.85 to 1.78) | 0 |
| Zambia | 1990 - 2013 | 2.86 (2.44 to 3.28) | 0 |
| Zambia | 1996 - 2019 | -1.44 (-3.74 to 0.91) | 0.205 |
| Zambia | 1999 - 1995 | -3.9 (-4.99 to -2.8) | 0 |
| Zambia | 2003 - 2002 | -1.94 (-2.22 to -1.67) | 0 |
| Zambia | 2011 - 2007 | -3.12 (-4.16 to -2.06) | 0 |
| Zambia | 2015 - 2010 | -1.84 (-2.58 to -1.09) | 0 |
| Zimbabwe | 1990 - 2016 | -7.02 (-8.77 to -5.24) | 0 |
| Zimbabwe | 1994 - 2019 | -13.01 (-18.89 to -6.71) | 0.001 |
| Zimbabwe | 1997 - 1999 | 18.21 (13.94 to 22.64) | 0 |
| Zimbabwe | 2001 - 2005 | 9.57 (8.61 to 10.55) | 0 |
| Zimbabwe | 2009 - 2019 | -0.51 (-1.03 to 0.01) | 0.056 |
