# Supplementary material for: Temporal trends in the prevalence and death of ischemic heart disease in women of childbearing age from 1990 to 2019: a multilevel analysis based on the Global Burden of Disease Study 2019
Source: Front Cardiovasc Med. 2024 Apr 22;11:1366832. doi: 10.3389/fcvm.2024.1366832 (PMC11070499; doi:10.3389/fcvm.2024.1366832)
Supplement: Supplementary file 2 [file Datasheet1.docx]

**Supplementary Method**

**Data sources**

Overview

The Global Burden of Disease (GBD) is a global epidemiological project that systematically and scientifically describes health loss owing to diseases, injuries, and risk factors at specific times. It quantifies the comparative magnitude of health loss by age, sex, and geography. Published in The Lancet in October 2020, GBD2019 utilizes, for the first time, a standardized and replicable method to provide an independent estimation for each of 204 countries and territories, as well as updated comprehensive data on fertility and migration(1). GBD 2019 contains major data additions, improvements, and methodological refinements. Mortality and life expectancy estimates have expanded to a total of 990 locations at the most detailed level, and new causes have been added to the fatal and nonfatal cause lists, for a total of 369 diseases and injuries (http://www.healthdata.org/gbd/about/protocol). In GBD 2019, each epidemiological indicator (incidence, prevalence, mortality, years lived with disability, years of life lost, and disability-adjusted life-years) of interest was evaluated according to age (23 age groups); gender (male, female, and combined); and geography (204 countries and territories that were grouped into 21 regions and seven super-regions). The GBD 2019 location hierarchy now includes all WHO member states. The GBD disease and injury analytical framework generated estimates for every year from 1990 to 2019. Diseases and injuries were organized into a leveled cause hierarchy from Level 1 to Level 4. Level 1 included three causes (communicable, maternal, neonatal, and nutritional diseases; noncommunicable diseases; and injuries). There are 22 Level 2 causes (including cardiovascular disease (CVD)), 174 Level 3 causes, and 301 Level 4 causes (including 131 Level 3 causes that are not further disaggregated at Level 4). In total, 364 causes are nonfatal, and 286 are fatal(1).

The GBD estimation process is based on identifying multiple relevant data sources for each disease or injury, including censuses, household surveys, civil registration and vital statistics, disease registries, health service use, air pollution monitors, satellite imaging, disease notifications, and other sources. Each of these types of data is identified from a systematic review of published studies, searches of government and international organization websites, published reports, primary data sources such as the Demographic and Health Surveys, and contributions of datasets by GBD collaborators. The analysis for CVD used 5507 data sources, including 3901 reporting deaths, 599 reporting incidence, 646 reporting prevalence, and 361 reporting other indicators. Each newly identified and obtained data source is given a unique identifier by a team of librarians and included in the Global Health Data Exchange (GHDx; http://ghdx.healthdata.org/). The GHDx makes publicly available the metadata for each source included in GBD as well as the data, where allowed by the data provider. The GHDx source (http://ghdx.healthdata.org/gbd-2019/data-input-sources) tool can be used to identify which sources were used for estimating any disease or injury outcome in any given location.

IHD prevalence and mortality were estimated using extensive data representing broad population segments. These data were derived from literature reviews and corroborated through research collaborations encompassing published scientific reports on registries and cohorts, data from cohort studies and registries, administrative health data, and population surveys (2). DisMod-MR software V2.1 and meta-regression-Bayesian, regularized, trimmed (MR-BRT) software were used to generate consistent disease estimates. Posterior distributions, ranging from the 25th to 975th order values across 1000 posterior draws, were used to produce UI for each metric (2).

Details of the flowcharts, definitions, input data, and modeling strategies can be accessed in Supplementary Appendix 1 of the GBD 2019 study (https://www.thelancet.com/cms/10.1016/S0140-6736(20)30925-9/attachment/7709ecbd-5dbc-4da6-93b2-3fd0bedc16cc/mmc1.pdf).

**Input data**

We selected “Cause of death or injury” from “GBD Estimate” query box, “Prevalence and Death” from “Measure” query box, and “Number and Rate” from “Metric” query box, “15-19 years, 20-24 years, 25-29 years, 30-34 years, 35-39years, 40-44 years, 45-49 years, and 15-49 years” from “Age” query box, “Female” from “Sex” query box

For attributable risks, we selected “Risk” from “Context” query box, “Death” from “Measure” query box, and “Percent” from “Metric” query box.

**Joinpoint analysis**

Joinpoint regression is also known as join point regression model. Its role is to find turning points in the trend of disease development and calculate the annual percent change (APC) in the years between turning points, as well as the overall Average annual percent change (AAPC), which is the greatest advantage. In general, the link point regression for GBD data goes through the following three steps: First, the log-linear model (ln y = *x) was adopted for piecewise regression, all possible join points were established with grid search method (GSM), and the corresponding mean squared errors (MSE) in each possible case were calculated. The grid point with the smallest MSE is selected as the connection point. Secondly, Monte Carlo permutation test was used to establish the optimal model of join point regression (i.e., the number of join points). We set the maximum number of potential join points to 5 and the minimum number of potential join points to 0. The permutation test starts from the number of join points k = 0 and k_max = 5. If k < k_max, set k = k+1 to continue the test until the model corresponding to k = k_max is selected as the optimal model. Finally, we use the annual percent change (APC) and average annual percent change (AAPC) calculated by the optimal model to quantify the annualized trend from 1990 to 2019. APC is calculated by the following formula: APC=(e-1) x100%, where is the regression coefficient of log-linear model lny=*x. AAPC is the average sum of APC weighted by the span w of each segment, which represents the general change trend of epidemic trend from 1990 to 2019. Specific details and definitions are obtained in the website (https://surveillance.cancer.gov/joinpoint/help.html).

**References**

1. Global burden of 369 diseases and injuries in 204 countries and territories, 1990-2019: a systematic analysis for the global burden of disease study 2019. *Lancet*. (2020) 396:1204-22. doi: 10.1016/S0140-6736(20)30925-9

2. Cao F, Li DP, Wu GC, He YS, Liu YC, Hou JJ, et al. Global, regional and national temporal trends in prevalence for musculoskeletal disorders in women of childbearing age, 1990-2019: an age-period-cohort analysis based on the global burden of disease study 2019. *Ann Rheum Dis*. (2024) 83:121-32. doi: 10.1136/ard-2023-224530
